# Cell Death in Neurodegenerative Diseases: Molecular Mechanisms and Therapeutic Targets

**DOI:** 10.1002/mco2.70915

**Published:** 2026-08-17

**Authors:** Tianjiao Li, Qian Zhang, Yichen Wu, Ruixue Huang

**Affiliations:** ^1^ Xiangya School of Public Health Central South University Changsha Hunan Province China; ^2^ Special Med Ctr Chinese Peoples Armed Police Force Wuhan Special Service Recuperation Center Tianjin China

**Keywords:** apoptosis, cell death, ferroptosis, necroptosis, neurodegenerative diseases

## Abstract

Neurodegenerative diseases (NDs) are marked by selective neuronal vulnerability and progressive failure of neural circuits. Increasing evidence indicates that neuronal loss is not driven by a single terminal event, but emerges from interacting regulated cell death (RCD) programs. These programs are closely coupled to mitochondrial injury, proteostatic collapse, lysosomal stress, metabolic imbalance, glial state transitions, and chronic neuroinflammation. Yet, how distinct death pathways are organized across cell types, disease stages, and disease‐specific microenvironments remains unresolved. This review examines RCD as an integrated pathogenic network in major NDs. Caspase and B‐cell lymphoma 2 (BCL2) family signaling, receptor‐interacting protein kinase 1 (RIPK1), RIPK3, and mixed lineage kinase domain‐like protein activation, NOD‐like receptor family pyrin domain containing 3 and gasdermin signaling, GPX4‐linked lipid peroxidation control, and autophagy lysosomal failure are discussed as convergent stress response modules rather than isolated pathways. Across Alzheimer's disease, Parkinson's disease, amyotrophic lateral sclerosis, and Huntington's disease, these modules shape neuronal fate through disease‐specific interactions with mitochondrial dysfunction, iron dyshomeostasis, inflammasome activation, and microglial metabolic remodeling. We further evaluate emerging therapeutic strategies that target cell death crosstalk, restore autophagy lysosomal competence, or improve delivery to the central nervous system, highlighting the importance of molecular selectivity, cellular context, disease stage, and translational feasibility.

## Graphical abstract

Regulated cell death pathways, including apoptosis, necroptosis, pyroptosis, ferroptosis, and autophagy‐dependent cell death, interact with mitochondrial dysfunction, proteostasis failure, lysosomal stress, glial remodeling, and neuroinflammation across major neurodegenerative diseases. These interconnected mechanisms contribute to neuronal vulnerability and identify therapeutic opportunities in pathway‐specific intervention, autophagy–lysosomal repair, and targeted delivery.

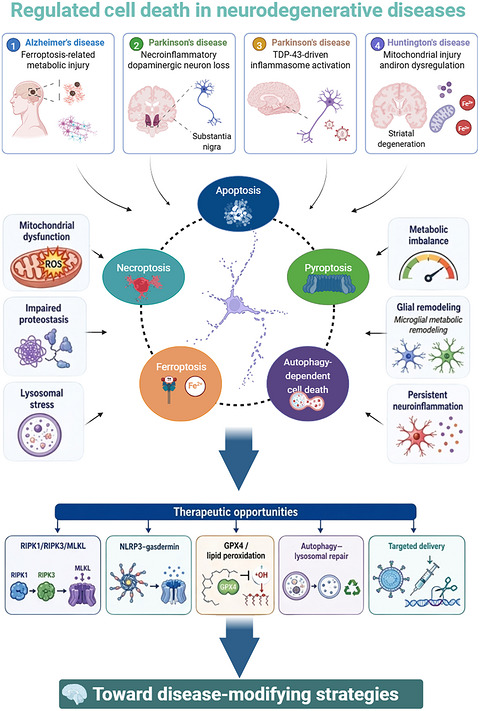

## 1 Introduction

Neurodegenerative diseases (NDs), characterized by progressive and selective neuronal loss, represent a growing global health challenge in aging societies. Major NDs, include Alzheimer's disease (AD), Parkinson's disease (PD), amyotrophic lateral sclerosis (ALS), and Huntington's disease (HD), contribute substantially to disability, mortality, and long‐term care burden worldwide [1, 2]. Globally, neurological conditions affected more than 3 billion people and caused approximately 443 million disability‐adjusted life years in 2021, making them the leading cause of disease burden [3]. Within this broad neurological burden, NDs are becoming increasingly prominent because of their progressive course, limited curative treatment options, and sustained impact on patients, caregivers, and health‐care systems [4].

Extensive research has identified protein misfolding, synaptic dysfunction, mitochondrial impairment, metabolic stress, and neuroinflammation as central pathological hallmarks of NDs. Despite these insights, available therapies are predominantly symptomatic and have limited capacity to prevent progressive neuronal loss. This limitation partly reflects the limited understanding of how regulated cell death (RCD) is initiated, regulated, and integrated during disease‐specific pathological progression. Elucidating the molecular mechanisms of neuronal death is thus critical for the discovery of disease‐modifying therapeutic targets [2, 5].

Apoptosis was initially considered the predominant form of neuronal death in NDs. However, this view has become insufficient to explain the diversity and complexity of neuronal loss observed across neurodegenerative conditions. Advances in single cell sequencing, spatial transcriptomics, in vivo imaging, and multiomics analyses have revealed substantial heterogeneity in death‐related cellular responses across different disease stages, brain regions, and cell types [6, 7]. Recent work indicates that RCD in neurodegeneration involves multiple interconnected modalities, such as apoptosis, necroptosis, pyroptosis, ferroptosis, and autophagy‐dependent cell death (ADCD) [8].

These RCD pathways are tightly linked to central pathological processes in NDs, including mitochondrial dysfunction, oxidative stress, lysosomal impairment, iron dyshomeostasis, lipid peroxidation, inflammasome activation, and glial immune responses [9, 10]. Notably, ferroptosis has attracted considerable attention due to its connection with iron accumulation, lipid peroxidation, and compromised antioxidant defense, leading to neuronal injury in AD, PD, HD, and ALS [11]. Inflammasome‐mediated pyroptotic signaling is increasingly acknowledged as a key driver of neuroinflammatory amplification in these disorders [12]. Furthermore, autophagy–lysosomal dysfunction contributes to neuronal death, especially in the context of AD‐associated autophagic stress and lysosomal injury [13]. Microglia play a central role in these processes, as microglial RCD and glial state transitions influence inflammatory damage and neuronal survival in the diseased central nervous system (CNS) [14].

Importantly, RCD pathways rarely operate in isolation. They are activated by shared upstream stressors and converge on overlapping molecular checkpoints, thereby contributing to disease progression in a manner shaped by disease stage, affected cell type, and inflammatory milieu. Recognizing this interconnected network is essential for accurately interpreting RCD markers in neurodegenerative tissues and for developing therapeutic strategies tailored to disease stage, cell type, and the inflammatory microenvironment. Accordingly, defining the molecular organization of RCD may help explain selective neuronal vulnerability and improve the precision of therapeutic intervention [2, 5, 15, 16].

This review critically examines the major forms of RCD involved in NDs, including apoptosis, necroptosis, pyroptosis, ferroptosis, and ADCD. Particular emphasis is placed on their molecular mechanisms, disease‐specific activation patterns, pathway crosstalk, and therapeutic implications in AD, PD, ALS, and HD. By integrating recent mechanistic and translational findings, this review aims to clarify the central role of RCD in neurodegeneration and highlight its potential as a target for disease‐modifying therapies.

## 2 Overview of RCD Pathways

Cell death plays a fundamental role in the development and homeostasis of the nervous system. During embryogenesis and synaptic refinement, regulated RCD eliminates redundant neurons and contributes to the formation of functional neural circuits [2]. Under pathological conditions, dysregulated activation of cell death pathways leads to irreversible neuronal injury via mechanisms including mitochondrial dysfunction, metabolic stress, impaired proteostasis, and inflammatory signaling. In NDs, including AD, PD, and ALS, these processes do not occur as isolated events. Instead, they form interconnected stress response modules that converge on mitochondrial failure, impaired protein clearance, glial immune activation, lipid peroxidation, lysosomal dysfunction, and chronic neuroinflammation [17]. This view aligns with current cell death classification, which emphasizes both morphological characteristics and molecular, functional, and disease‐specific regulatory mechanisms [18].

**FIGURE 1:**
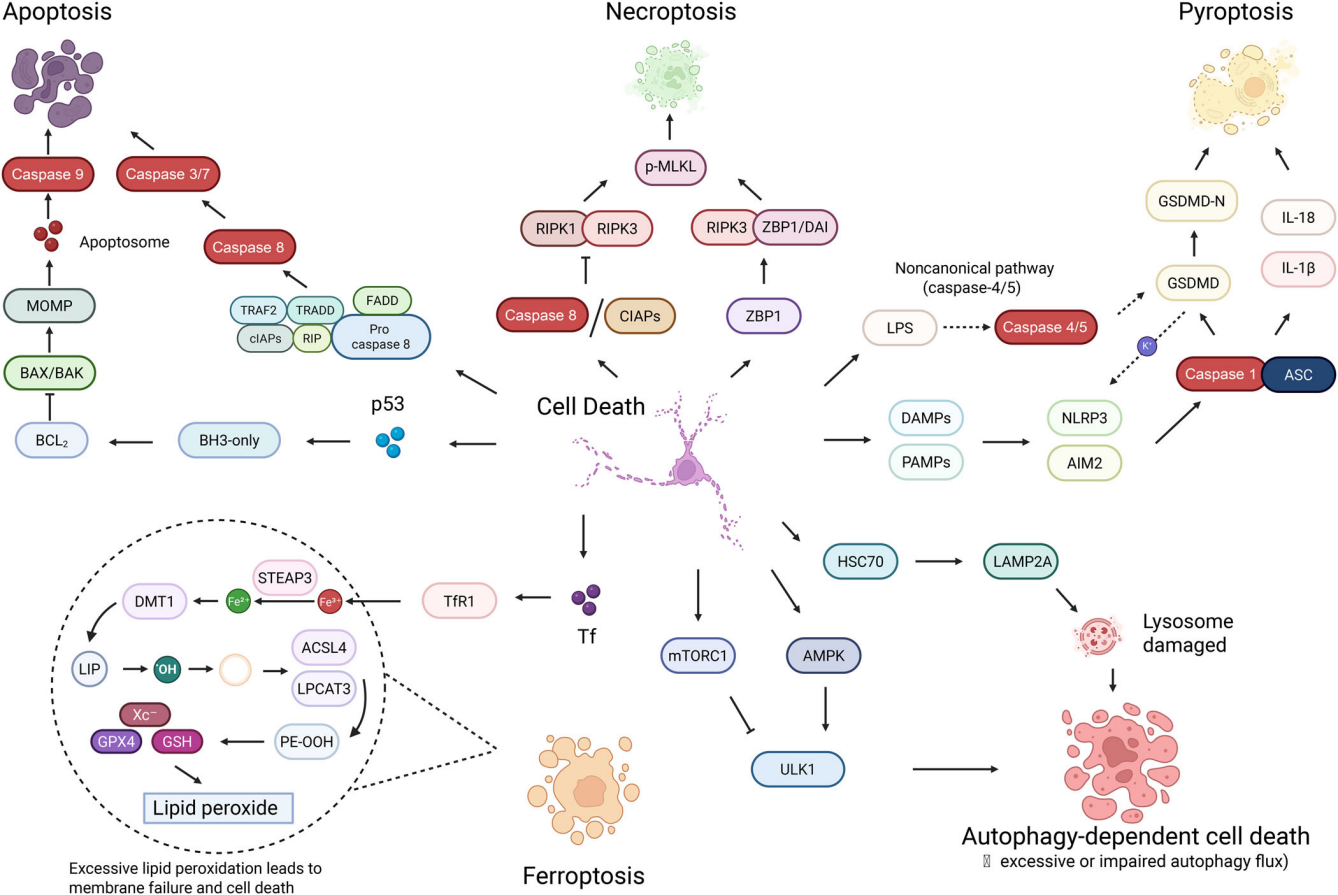
Schematic overview of RCD pathways in neurodegenerative diseases. This schematic summarizes five major RCD pathways implicated in neurodegenerative diseases: apoptosis, necroptosis, pyroptosis, ferroptosis, and autophagy‐dependent cell death. These pathways converge on shared stress nodes, including mitochondrial dysfunction, oxidative and lipid redox imbalance, proteostatic stress, lysosomal impairment, and neuroinflammatory signaling. Apoptosis involves intrinsic mitochondrial signaling through BAX/BAK‐mediated MOMP, apoptosome formation, and Caspase‐9 and Caspase‐3/7 activation, as well as extrinsic death receptor signaling through FADD and Caspase‐8. Caspase‐8 also links apoptosis to necroptosis, because reduced Caspase‐8 activity favors RIPK1/RIPK3 activation, MLKL phosphorylation, and membrane permeabilization. Pyroptosis is mediated by canonical inflammasome signaling involving NLRP3 or AIM2, ASC, Caspase‐1, gasdermin D cleavage, and IL‐1β and IL‐18 release, while Caspase‐4/5 contributes to noncanonical pyroptosis. Ferroptosis is driven by iron‐dependent lipid peroxidation caused by altered iron handling, System Xc− and GSH insufficiency, impaired GPX4 activity, and oxidized phospholipid accumulation. Autophagy‐dependent cell death is associated with impaired autophagic flux, defective cargo clearance, lysosomal dysfunction, and lysosomal membrane permeabilization. These interconnected pathways jointly shape neuronal vulnerability, inflammatory amplification, and progressive neurodegeneration. Abbreviations: RCD, regulated cell death; BAX, BCL2‐associated X protein; BAK, BCL2 antagonist/killer; MOMP, mitochondrial outer membrane permeabilization; FADD, Fas‐associated protein with death domain; RIPK1, receptor‐interacting protein kinase 1; RIPK3, receptor‐interacting protein kinase 3; MLKL, mixed lineage kinase domain‐like protein; NLRP3, NOD‐like receptor family pyrin domain containing 3; AIM2, absent in melanoma 2; ASC, apoptosis‐associated speck‐like protein containing a CARD; IL‐1β, interleukin‐1β; IL‐18, interleukin‐18; System Xc−, cystine/glutamate antiporter; GSH, glutathione; GPX4, glutathione peroxidase 4.

Distinguishing among different RCD modalities remains essential for understanding the pathogenesis of NDs. Such distinction also helps clarify how distinct death programs are triggered, coordinated, and amplified during disease progression. Apoptosis is a relatively noninflammatory form of RCD, primarily mediated by intrinsic mitochondrial or extrinsic death receptor pathways, and is characterized by cell shrinkage, chromatin condensation, and maintenance of plasma membrane integrity [2]. Necroptosis and pyroptosis are inflammatory forms of RCD. Necroptosis is chiefly controlled by receptor‐interacting protein kinase 1 (RIPK1), receptor‐interacting protein kinase 3 (RIPK3), and mixed lineage kinase domain‐like protein (MLKL), while pyroptosis is triggered by inflammasome activation, gasdermin cleavage, membrane pore formation, and the release of inflammatory cytokines [15]. Ferroptosis is an iron‐dependent process defined by lipid peroxidation and compromised antioxidant defenses, a mechanism particularly significant for neurons due to their high metabolic activity and lipid‐rich membranes. Autophagy maintains cellular homeostasis, but persistent autophagic stress, lysosomal dysfunction, or impaired autophagic flux may promote neuronal injury under pathological conditions [17]. Together, these modalities should be viewed as interacting death programs that are engaged by shared upstream stressors and that collectively contribute to neuronal loss, glial dysfunction, and disease progression. Their primary morphological, ultrastructural, and regulatory features are summarized in Table 1 and Figure 1.

**TABLE 1:** Modes of cell death and related processes: triggers, mechanisms, morphology, inflammation, and representative references.

Mode of cell death/process	Common triggers	Core molecular events and pathways	Morphological and ultrastructural features	Inflammatory consequences	Representative references
Apoptosis	DNA damage; growth factor deprivation; toxic protein aggregation; mitochondrial stress	Intrinsic pathway: BAX/BAK‐mediated MOMP, cytochrome *c* release, APAF1‐dependent apoptosome assembly, Caspase‐9 → Caspase‐3/7 activation. Extrinsic pathway: FAS or TNFR1‐induced DISC formation and Caspase‐8 activation with mitochondrial amplification	Cell shrinkage, chromatin condensation, nuclear fragmentation, membrane blebbing, apoptotic body formation; plasma membrane integrity largely preserved until late stages	Rapid phagocytic clearance prevents intracellular content release and limits secondary inflammation	[19]
Necroptosis	TNF signaling under Caspase‐8 inhibition; viral infection; PRR activation; metabolic stress	RIPK1 kinase activation and RIPK1–RIPK3 necrosome assembly; RIPK3‐mediated MLKL phosphorylation, oligomerization, and plasma membrane insertion	Cell and organelle swelling, early plasma membrane rupture, release of cytosolic contents; no apoptotic bodies	DAMP release following membrane rupture activates innate immune cells such as microglia and macrophages	[20]
Pyroptosis	Inflammasome activation (e.g., NLRP3); PAMPs; DAMPs; proteotoxic stress	Canonical Caspase‐1 or noncanonical Caspase‐4/5/11 activation; gasdermin D cleavage; pore formation coupled to IL‐1β and IL‐18 maturation	Rapid cell swelling, large membrane pores, abrupt membrane rupture with cytokine release	Gasdermin‐mediated permeabilization amplifies local and systemic inflammatory cascades	[21]
Ferroptosis	System Xc^−^ inhibition; cysteine and glutathione depletion; GPX4 inactivation; iron overload; PUFA phospholipid peroxidation	Iron‐dependent lipid hydroperoxide accumulation due to GPX4 failure; propagation of lipid radical chain reactions; parallel suppression by FSP1–CoQ10 axis	Condensed, shrunken mitochondria with increased membrane density and reduced cristae; preserved nuclear morphology; absence of caspase activation	Oxidized phospholipids and lipid‐derived danger signals propagate inflammatory and oxidative stress	[22]
ADCD	Prolonged nutrient deprivation; sustained cellular stress; excessive autophagic demand; lysosomal dysfunction	Pathological escalation of ATG‐dependent autophagic flux causally linked to cell death; autosis as a Na^+^,K^+^‐ATPase‐dependent subtype	Accumulation of autophagosomes and autolysosomes; autosis marked by perinuclear space expansion and membrane rearrangement	Inflammatory outcome depends on lysosomal and plasma membrane integrity; rupture promotes inflammatory signaling	[23]

Abbreviations: ADCD,Autophagy‐dependent cell death;APAF1, apoptotic protease activating factor 1; ATG, autophagy‐related; BAK, BCL2 antagonist/killer; BAX, BCL2‐associated X protein; CoQ10, coenzyme Q10; DAMPs, danger‐associated molecular patterns; DISC, death‐inducing signaling complex; FAS, Fas cell surface death receptor; FSP1, ferroptosis suppressor protein 1; GPX4, glutathione peroxidase 4; IL‐18, interleukin‐18; IL‐1β, interleukin‐1β; MLKL, mixed lineage kinase domain‐like protein; MOMP, mitochondrial outer membrane permeabilization; Na+,K+‐ATPase, sodium‐potassium adenosine triphosphatase; NLRP3, NOD‐like receptor family pyrin domain containing 3; PAMPs, pathogen‐associated molecular patterns; PRR, pattern recognition receptor; PUFA, polyunsaturated fatty acid; RIPK1, receptor‐interacting protein kinase 1; RIPK3, receptor‐interacting protein kinase 3; System Xc−, cystine/glutamate antiporter; TNF, tumor necrosis factor; TNFR1, tumor necrosis factor receptor 1.

### 2.1 Apoptosis

Apoptosis remains one of the most extensively studied forms of RCD, but in NDs its relevance should not be reduced to a canonical caspase cascade [24, 25]. In the diseased nervous system, apoptosis is triggered by convergent stress signals, including mitochondrial injury, ROS accumulation, Ca^2+^ imbalance, ER stress, death receptor activation, and impaired clearance [26]. These signals converge on mitochondrial outer membrane permeabilization (MOMP), primarily mediated by B‐cell lymphoma (BCL)2‐associated X protein/BCL2 antagonist/killer (BAX/BAK) activation, leading to cytochrome c release, apoptosome assembly, and downstream activation of Caspase‐9 and Caspase‐3/7 [27]. In parallel, apoptosis‐inducing factor and endonuclease G translocation may drive caspase‐independent DNA fragmentation under severe mitochondrial or oxidative stress [28, 29]. Death receptor signaling Fas, tumor necrosis factor receptor 1 (TNFR1), or death receptor 4/death receptor 5 (DR4/DR5) activates death‐inducing signaling complex (DISC) and Caspase‐8, which further amplifies mitochondrial apoptosis through BID cleavage [30]. Apoptotic cells are ultimately cleared as apoptotic bodies by phagocytes, limiting damage‐associated molecular patterns (DAMPs)release and explaining why apoptosis is generally less inflammatory than necroptosis or pyroptosis [31, 32]. Therefore, in NDs, apoptosis should be interpreted as a stress‐responsive module linking mitochondrial dysfunction, protease activation, synaptic degeneration, and clearance failure rather than an isolated terminal pathway.

In numerous NDs, Caspase‐3 activation is consistently observed during early neuronal injury, supporting the view that apoptosis is a fundamental pathological mechanism involved in neuronal loss and disease progression [27, 32]. However, Caspase‐3 activation in the nervous system requires cautious interpretation. Beyond its established role in apoptosis, Caspase‐3 is also involved in synaptic remodeling, developmental pruning, and sublethal synaptic dysfunction. Therefore, the presence of cleaved Caspase‐3 should not be regarded as definitive evidence of irreversible neuronal death [25]. This distinction is particularly important in NDs, where early caspase activation may reflect a continuum from synaptic degeneration to terminal cell death rather than a single irreversible event.

Furthermore, apoptosis should not be considered an isolated mechanism in neurodegenerative pathology. Caspase‐8 functions as a molecular checkpoint that balances apoptosis, necroptosis, and inflammatory signaling. Under physiological conditions, Caspase‐8 inhibits necroptosis by cleaving RIPK1 and restricting activation of the RIPK3 and MLKL pathway. Experimental studies show that RIPK1 cleavage by Caspase‐8 is essential for limiting both apoptosis and necroptosis. By contrast, impaired Caspase‐8 activity permits formation of RIPK1/RIPK3‐dependent necrosomes and subsequent MLKL‐mediated membrane rupture [33]. Thus, caspase inhibition does not invariably confer neuroprotection. Instead, it may redirect cell death signaling from apoptosis toward more inflammatory necroptosis or mixed cell death phenotypes. This pathway shift is particularly relevant when apoptotic execution is incomplete, delayed, or pharmacologically inhibited, as caspase suppression may reduce classical apoptotic markers while simultaneously enhancing inflammatory regulated necrosis.

### 2.2 Necroptosis

Necroptosis is a regulated inflammatory RCD pathway governed by RIPK1/RIPK3/MLKL signaling [20, 34]. In NDs, its relevance extends beyond an alternative route engaged when apoptotic execution is impaired [35]. Persistent tumor necrosis factor α (TNF‐α) exposure, interferon signaling, mitochondrial impairment, protein aggregation, oxidative stress, and reduced Caspase‐8 activity may favor RIPK1 kinase activation, RIPK3 recruitment, necrosome formation, and MLKL phosphorylation [34, 36, 37]. However, activation of this axis in neural tissue does not necessarily indicate immediate membrane rupture. RIPK1, RIPK3, and MLKL may also participate in inflammatory priming, axonal injury, glial reactivity, metabolic disturbance, vesicular trafficking, and intercellular communication before terminal lytic death occurs [38, 39]. Accordingly, phosphorylated RIPK1, RIPK3, or MLKL in neurodegenerative tissues should be interpreted as evidence of necroptosis‐associated pathway activation in a defined cellular and pathological context, rather than as standalone evidence of completed neuronal necroptosis. This distinction is particularly relevant because TNFR1 signaling in diseased neural tissue may produce mixed cellular outcomes, including survival signaling, inflammatory priming, apoptotic activation, and necroptotic execution [20, 34]

In ALS, experimental evidence indicates that RIPK1 can promote axonal degeneration through inflammatory and necroptosis‐related mechanisms. In optineurin deficient models, enhanced RIPK1 signaling drives progressive dysmyelination and axonal degeneration and activates necroptosis‐related machinery in the CNS, linking RIPK1 activation to neuronal injury and inflammatory disease amplification [40]. When necroptotic membrane disruption occurs, DAMPs, including high mobility group box 1 (HMGB1) and adenosine triphosphate(ATP), are released together with other intracellular components and are recognized by dendritic cells, macrophages, and microglia, thereby amplifying proinflammatory signaling [34]. In NDs, such release after necroptotic membrane rupture contributes to sustained neuroinflammation and chronic glial activation. This mechanism is particularly important in the CNS, where local membrane rupture may convert injury within individual cells into a broader inflammatory cascade involving microglia, astrocytes, and neighboring neurons [40].

Nevertheless, necroptosis is not uniformly supported across all ALS models. For example, genetic inactivation of RIPK1 kinase activity in superoxide dismutase 1 (SOD1) transgenic mice did not improve muscle strength, nerve function, motor neuron survival, or neuroinflammation, and phosphorylated RIPK1 was not consistently detected in spinal cords from patients with ALS [41]. These observations do not exclude a pathogenic role for RIPK1 signaling, but they suggest that necroptosis may vary according to disease genotype, glial status, axonal injury pattern, and inflammatory stage. Therefore, therapeutic strategies targeting necroptosis should be guided by direct evidence of pathway activation in defined cell types and disease stages, rather than by pathway marker detection alone.

In addition to TNFR1 signaling, Toll‐like receptor 3 (TLR3) or TLR4, together with Toll/interleukin‐1 receptor(TIR)‐domain‐containing adapter‐inducing interferon‐β can recruit and activate RIPK3, thereby initiating necroptosis without requiring RIPK1 [42]. Z‐DNA binding protein 1 (ZBP1) provides another innate immune route linking nucleic acid sensing to necroptotic and pyroptotic signaling. Under viral infection, interferon signaling, or cellular stress, ZBP1 interacts with RIPK3 through its RHIM domain and forms a ZBP1/RIPK3/MLKL signaling axis, which can directly trigger necroptosis and may also activate pyroptotic signaling [43, 44]. Notably, ZBP1 expression is markedly increased in neuroinflammatory lesions, supporting its role in immune‐associated necroptotic neuronal injury. Together, these findings indicate that necroptosis connects death receptor signaling, innate immune sensing, and inflammatory amplification, rather than functioning only as a backup route when apoptosis is blocked.

### 2.3 Pyroptosis

Pyroptosis is an inflammatory RCD program initially characterized in macrophages and monocytes by cell swelling, membrane ballooning, and rupture [45, 46]. In NDs, however, pyroptosis is more appropriately interpreted as an inflammasome‐ and gasdermin‐linked inflammatory signaling process that connects innate immune sensing, cytokine release, glial activation, and secondary neuronal injury [47, 48]. Persistent danger signals from damaged neurons, misfolded proteins, mitochondrial DNA (mtDNA), extracellular ATP, oxidized lipids, and systemic immune activation may chronically stimulate canonical NLRP3/ASC/Caspase‐1 signaling and, in specific contexts, noncanonical lipopolysaccharide (LPS) sensing through Caspase‐4/5 or Caspase‐11 [21, 49]. Gasdermin D (GSDMD) cleavage allows its N‐terminal fragment to form membrane pores, leading to ionic imbalance, cytokine release, and membrane permeabilization [46, 50, 51]. However, GSDMD pore formation does not necessarily progress directly to terminal lysis, because endosomal sorting complexes required for transport (ESCRT)‐dependent repair can limit membrane damage and delay rupture [52]. Thus, inflammasome priming, GSDMD cleavage, IL‐1β/IL‐18 release, sublytic pore formation, and completed pyroptotic death should be distinguished when interpreting pyroptosis markers in neural tissue.

Available evidence more strongly supports inflammasome activation in microglia and other innate immune cells, while direct neuronal pyroptosis remains less conclusively demonstrated. For instance, amyloid β (Aβ) oligomers activate the NLRP3/ASC/Caspase‐1 inflammasome in microglia, leading to IL‐1β release, inflammatory amplification, synaptic dysfunction, and impaired neuronal communication. In amyloid precursor protein/presenilin 1 (APP/PS1) transgenic mice, inhibition of NLRP3 or Caspase‐1 improves cognitive deficits, supporting a central role for inflammasome‐driven neuroinflammation in disease progression [53, 54]. Additional evidence shows that NLRP3 activation exacerbates amyloid pathology and cognitive decline in these models [53]. By contrast, many studies infer neuronal pyroptosis mainly from colocalization of inflammasome components with neuronal markers. Such colocalization does not prove pore formation, membrane rupture, or autonomous neuronal death. Thus, in AD, current evidence more strongly supports microglial inflammasome activation and inflammasome‐mediated neuronal dysfunction than direct neuronal lytic pyroptosis.

In PD, there is more direct experimental evidence linking inflammasome and GSDMD signaling to neurodegenerative pathology. Preformed fibrils of α‐synuclein (α‐syn) activate the NLRP3 inflammasome in microglia, leading to Caspase‐1‐dependent GSDMD cleavage and release of IL‐1β, IL‐18, and HMGB1 [55, 56, 57, 58]. These inflammatory mediators contribute to a self‐sustaining inflammatory environment that accelerates dopaminergic neuron degeneration and worsens motor deficits. Recent studies indicate that inflammasome signaling can also occur within dopaminergic neurons. Loss of parkin function facilitates neuronal NLRP3 inflammasome assembly by impairing NLRP3 ubiquitination and increasing mitochondrial ROS, whereas blocking this process preserves dopaminergic neurons in familial and sporadic models [59]. In addition, deletion of *Gsdmd* reduces dopaminergic neuronal loss, microglial activation, and 1‐methyl‐4‐phenyl‐1,2,3,6‐tetrahydropyridine (MPTP)‐induced neuropathology, while disulfiram ameliorates Parkinsonian pathology by inhibiting GSDMD pore formation [60]. These findings support a pathogenic role for inflammasome and GSDMD signaling in PD, although the relative contributions of microglial pyroptosis, neuronal inflammasome activity, and true neuronal lytic pyroptosis remain to be clarified.

Compared with PD models, findings from ALS indicate that pyroptosis pathway activation does not necessarily establish a causal role in disease pathogenesis. In symptomatic SOD1^G93A^ mice, GSDMD is activated in the spinal cord and is predominantly detected in ionized calcium‐binding adaptor molecule 1 (Iba1)‐positive microglia. However, genetic deletion of *Gsdmd* does not improve disease onset, weight loss progression, grip strength, astrogliosis, microgliosis, or motor neuron survival, and instead produces a modest but statistically significant increase in mortality [61]. These results suggest that pyroptosis‐associated molecules may be upregulated as part of a broader inflammatory response without serving as essential drivers of neuronal degeneration in every neurodegenerative disorder.

Pyroptosis‐targeted intervention requires separation of inflammasome priming, cytokine secretion, sublytic pore formation, and terminal lytic death. Excessive inflammasome activation and persistent GSDMD‐mediated membrane permeabilization may contribute to inflammatory tissue injury in selected neurodegenerative conditions [62, 63]. However, broad inhibition of NLRP3, Caspase‐1, or GSDMD may also affect host defense, debris clearance, and adaptive immune responses, which should be considered when evaluating therapeutic feasibility.

Pyroptosis also intersects with necroptosis and apoptosis through shared mediators, including Caspase‐8, ASC, RIPK3, and gasdermin family proteins. This convergence supports the view that inflammatory cell death in neurodegeneration proceeds as an interconnected process rather than a linear cascade, shaped by cellular stress, innate immune activation, and the inflammatory milieu [64]. The key issue is therefore not simply whether pyroptosis markers are present in neurodegenerative disorders, but which cell types undergo inflammasome activation, whether gasdermin‐mediated lysis actually occurs, and how these events interact with other forms of cell death, mitochondrial dysfunction, and metabolic stress.

### 2.4 Ferroptosis

Ferroptosis is an iron‐dependent form of RCD characterized by disrupted lipid redox homeostasis and lipid peroxidation, rather than by caspase activation or gasdermin/MLKL‐mediated pore formation [22, 65, 66]. In NDs, its relevance lies in the convergence of iron dyshomeostasis, mitochondrial dysfunction, peroxidation of polyunsaturated fatty acid (PUFA)‐containing phospholipids, oxidative stress, and selective neuronal vulnerability. Although ferroptotic cells may exhibit shrunken mitochondria, reduced cristae, increased mitochondrial membrane density, and relatively preserved nuclear architecture [67], these ultrastructural features are insufficient to establish ferroptosis in neurodegenerative tissues because they may overlap with other forms of neuronal injury [66]. More stringent interpretation requires coordinated evidence of iron accumulation, lipid peroxide buildup, impaired glutathione peroxidase 4 (GPX4)–GSH/System Xc^−^ defense, and rescue by ferroptosis‐targeted pharmacological or genetic intervention. Mechanistically, expansion of the labile iron pool through transferrin receptor‐mediated uptake, six‐transmembrane epithelial antigen of prostate 3‐dependent ferric iron reduction, divalent metal transporter 1 (DMT1)‐mediated iron release, and Fenton chemistry can promote lipid radical formation [66, 68]. Lipid peroxidation is further shaped by acyl‐CoA synthetase long‐chain family member 4 (ACSL4) and lysophosphatidylcholine acyltransferase 3‐dependent enrichment of PUFA‐containing phospholipids and 15‐lipoxygenase‐mediated oxidation, which is particularly relevant to neurons with high oxygen consumption, extensive membrane architecture, and lipid‐rich synaptic and axonal compartments [66, 69]. The GPX4–GSH/System Xc^−^ axis represents a major antioxidant checkpoint that restrains lipid hydroperoxide accumulation. When System Xc^−^ function is impaired, GSH availability declines, or GPX4 activity is reduced, lipid peroxide detoxification fails, and ferroptotic injury is amplified [69]. Genetic evidence further supports the importance of this checkpoint, as conditional deletion of *Gpx4* in adult neurons causes rapid neurological deterioration, paralysis, and death, indicating that neuronal GPX4 is required to prevent ferroptotic degeneration in vivo [70].

Growing evidence implicates ferroptosis in selective neuronal vulnerability. In AD, affected brain regions show increased iron deposition, enhanced lipid peroxidation, and reduced GPX4 expression. Similarly, in PD, excessive iron accumulation in the substantia nigra, together with α‐syn aggregation, accelerates oxidative damage and dopaminergic neuronal loss [69]. Ferroptosis therefore links metal dysregulation, mitochondrial dysfunction, oxidative lipid injury, and progressive neuronal loss. This pathway also interacts with neuroinflammation, because lipid peroxidation products and oxidized phospholipids can act as danger signals that activate microglia and amplify cytokine production. Recent studies further suggest that ferroptosis‐related injury is not restricted to neurons, although neuronal loss remains the major pathological outcome. For example, in a neuron, astrocyte, and microglia triculturing system, iron‐loaded microglia showed high susceptibility to ferroptosis and contributed to neurodegeneration through effects on neighboring neurons [71]. Astrocyte‐derived lactoferrin can reduce neuronal iron accumulation and increase GPX4 expression, thereby suppressing neuronal ferroptosis in animal and cellular models [72]. These findings indicate that glial cells may either exacerbate or mitigate ferroptotic neuronal death by regulating iron metabolism, antioxidant defense, and inflammatory lipid signaling, while the main therapeutic goal remains protection of vulnerable neurons.

Pharmacological modulation of ferroptosis has been explored through iron chelation, lipid peroxidation inhibition, GPX4‐centered strategies, and nuclear factor erythroid 2‐related factor 2 (NRF2) pathway activation, with neuroprotective effects reported in experimental models [73]. These interventions converge on restoration of iron‐redox balance and suppression of lipid peroxide accumulation, two key determinants of ferroptotic susceptibility in neurons. Evidence further indicates that modulation of iron handling and endogenous antioxidant capacity may attenuate ferroptosis‐associated neuronal injury, although its disease‐modifying relevance in NDs requires further validation [74].

### 2.5 Autophagy‐Dependent Cell Death

Autophagy is essential for neuronal proteostasis because long lived postmitotic neurons depend on continuous lysosomal clearance to remove damaged organelles, misfolded proteins, and aggregation prone substrates [75]. Genetic evidence supports this dependence. In mice, neuronal deletion of autophagy‐related 5 (*Atg5)* or *autophagy‐related 7 (Atg7)* causes p62/sequestosome 1 (SQSTM1) and ubiquitinated aggregate accumulation, axonal swelling, neuronal vacuolization, motor dysfunction, neurodegeneration, and premature death within weeks [73, 76]. In humans, biallelic *ATG7* mutations severely impair autophagic flux and lead to cerebellar hypoplasia and neurological deficits [77]. These findings indicate that intact autophagy is required for neuronal maintenance, but they do not imply that autophagy is uniformly protective. Under hypoxia, excitotoxicity, energy depletion, or chronic injury, excessive, incomplete, or dysregulated autophagic flux may contribute to ADCD, which is characterized by abnormal autophagic vacuole accumulation and occurs without canonical caspase activation or MLKL phosphorylation [78, 79].

In NDs, the critical issue is often not autophagy induction itself, but whether autophagic flux proceeds to effective lysosomal degradation. When lysosomal function is impaired or autophagosome lysosome fusion is blocked, accumulated autophagic vacuoles may promote lysosomal membrane permeabilization (LMP) and release cathepsins B and L into the cytosol, thereby linking defective clearance to lysosomal cell death [80, 81]. In AD patient brains and APP/PS1 mice, autophagosomes and autophagic vacuoles accumulate within axons, suggesting that impaired autophagic clearance, rather than enhanced induction alone, drives pathological buildup and provides intracellular sites for Aβ generation and accumulation [82]. Flux tracing with a neuronal monomeric red fluorescent protein/enhanced green fluorescent protein/microtubule‐associated protein 1 light chain 3 (mRFP/eGFP/LC3) reporter in five AD mouse models further showed that autophagosome formation, transport, and fusion were relatively preserved, whereas autolysosomal acidification declined before Aβ plaque formation. This defect was associated with reduced reporter; vacuolar‐type H^+^‐ATPase (v‐ATPase) activity, accumulation of Aβ and APP β‐carboxyl‐terminal fragment (APP‐βCTF) within swollen deacidified autolysosomes, and later emergence of the plaque‐associated neuronal death with highly organized structure (PANTHOS [“poisonous flower”]) phenotype, followed by LMP, cathepsin release, and lysosomal cell death. The finding that 91.7% of total 3D6‐positive β‐amyloid signal was associated with PANTHOS lesions supports the view that defective autolysosomal clearance precedes and contributes to amyloid pathology rather than merely reflecting late stage degeneration [83].

Autophagy‐related injury is also shaped by upstream initiation, axonal transport, chaperone‐mediated autophagy (CMA), and lysosomal biogenesis. The unc‐51‐like autophagy activating kinase 1 (ULK1) complex integrates mechanistic target of rapamycin Complex 1 (mTORC1) and AMP‐activated protein kinase (AMPK) signaling to regulate autophagosome initiation [84]. In PD models, pathogenic leucine‐rich repeat kinase 2 (LRRK2) mutations such as G2019S disrupt ULK1‐dependent autophagic and mitochondrial clearance, leading to defective flux, α‐syn accumulation, mitochondrial dysfunction, and dopaminergic neuronal injury [85, 86, 87]. During autophagosome maturation, LC3 lipidation supports autophagosomal membrane formation, whereas neuronal autophagosomes generated in distal axons require retrograde transport to the soma for lysosomal degradation [88]. Accumulation of LC3‐II and p62 in AD and PD brains is therefore more consistent with stalled flux and defective lysosomal clearance than with simple autophagy activation [89]. In parallel, CMA contributes to α‐syn turnover through heat shock cognate 70 and lysosome‐associated membrane protein type 2A (LAMP2A). Wild‐type α‐syn is degraded through this pathway, whereas PD linked A53T and A30P α‐syn bind LAMP2A but fail to translocate, thereby blocking CMA substrate degradation [90]. Glucocerebrosidase gene (*GBA1*) mutations further impair lysosomal acidification by reducing glucocerebrosidase activity and aggravate α‐syn aggregation [91]. Transcription factor EB (TFEB) coordinates autophagy–lysosome pathway (ALP) activity by regulating lysosomal biogenesis, autophagosome maturation, and substrate clearance [92]. Although TFEB activation enhances Aβ, tau, and α‐syn degradation and improves cognitive or motor outcomes in AD and PD models [93], excessive TFEB activity may promote lysosomal expansion, increased autophagosome formation, and ADCD, indicating that ALP enhancement requires controlled rather than indiscriminate activation [92].

These findings indicate that autophagy influences neuronal survival only when induction, flux progression, lysosomal acidification, and substrate clearance remain coordinated. Insufficient clearance allows damaged mitochondria, oxidized proteins, and aggregation‐prone substrates to accumulate, whereas incomplete autophagic progression increases vacuolar stress and lysosomal instability. Defective mitophagy further increases mitochondrial ROS and sensitizes cells to apoptosis, necroptosis, and ferroptosis, while LMP promotes cathepsin release and amplifies inflammatory and death signaling. Thus, autophagy links proteostatic stress, mitochondrial dysfunction, lysosomal injury, inflammation, and RCD crosstalk in neurodegeneration.

## 3 Crosstalk and Integration Among Cell Death Pathways

During NDs progression, RCD pathways rarely operate in isolation or follow a strictly linear sequence. Apoptosis, necroptosis, pyroptosis, ferroptosis, and ADCD are interconnected through convergent stress signaling, overlapping regulatory mechanisms, and reciprocal inflammatory amplification. This section focuses on the major regulatory nodes coordinating these pathways. Caspase‐8 regulates the balance among apoptosis, necroptosis, and inflammatory signaling. Mitochondria function as a central pathological hub connecting intrinsic apoptosis, ROS accumulation, inflammasome activation, iron‐dependent lipid peroxidation, and defective mitophagy. Inflammatory signaling links pyroptosis, necroptosis, and ferroptosis through danger‐associated molecular pattern release, cytokine production, and glial immune activation. Microglial immunometabolism further influences how inflammatory signaling is translated into cytokine secretion, membrane permeabilization, lipid peroxidation, phagocytic repair, or sustained neuroinflammatory damage.

### 3.1 Regulatory Role of Caspase‐8 in Apoptosis, Necroptosis, and Inflammatory Signaling

Caspase‐8 is traditionally regarded as the initiator protease of the extrinsic apoptotic pathway. After activation of death receptors such as Fas and TNFR1, Caspase‐8 is recruited to the DISC through fas‐associated protein with death domain (FADD)‐mediated interactions, thereby initiating downstream apoptotic signaling [94, 95]. In NDs, however, Caspase‐8 has functions beyond classical apoptosis. It acts as a molecular switch that influences whether stressed neurons or glial cells undergo relatively noninflammatory apoptosis or shift toward RIPK1, RIPK3, and MLKL‐dependent necroptosis.

This regulatory function is largely mediated by the ability of Caspase‐8 to restrain necroptotic signaling. Under physiological conditions, Caspase‐8 cleaves RIPK1 and RIPK3, thereby preventing the formation of RHIM‐dependent RIPK1 and RIPK3 complexes and subsequent MLKL activation [96, 97]. Genetic studies support this mechanism. Caspase‐8 deficiency causes embryonic lethality, whereas simultaneous deletion of RIPK3 or MLKL rescues this phenotype. This finding indicates that Caspase‐8 is required not only for apoptotic induction but also for suppression of the RIPK3 and MLKL necroptotic signaling axis [96, 98]. When Caspase‐8 activity is inhibited by pan caspase inhibitors such as zVAD‐fmk or by genetic disruption, TNF‐α stimulation does not lead to canonical apoptosis. Instead, RIPK1 and RIPK3 form the necrosome, promote MLKL oligomerization, and disrupt the plasma membrane, thereby redirecting apoptotic stress toward a more inflammatory necrotic cell death phenotype [99, 100].

Caspase‐8 also intersects with inflammasome signaling. In inflammatory settings, Caspase‐8 can colocalize with ASC and enhance NLRP3 inflammasome activation. When Caspase‐1 activity is limited or inflammasome function is altered, Caspase‐8 may contribute to the processing of proinflammatory cytokines through the NLRP3/ASC complex [101]. This dual role is particularly relevant to neurodegenerative pathology. Broad caspase inhibition does not necessarily confer neuroprotection. It may suppress apoptotic cell death while promoting necroptosis or mixed inflammatory cell death phenotypes. For example, in APP/PS1 mice, inhibition of RIPK1 kinase activity reduces microglial activation, decreases TNF‐α and IL‐1β levels, and improves cognitive performance in the Morris water maze [101]. Similarly, in primary cortical neurons, inhibition of Caspase‐8 promotes necrotic cell death dependent on RIPK1 and RIPK3, which can be alleviated by necroptosis inhibitors such as necrostatin‐1 (Nec‐1) or GSK’872 [102]. These findings indicate that therapeutic modulation of caspase pathways requires careful evaluation, because suppression of apoptosis may redirect neuronal injury toward more inflammatory forms of cell death.

### 3.2 Mitochondria as a Convergent Platform for Death Execution and Inflammatory Amplification

**FIGURE 2:**
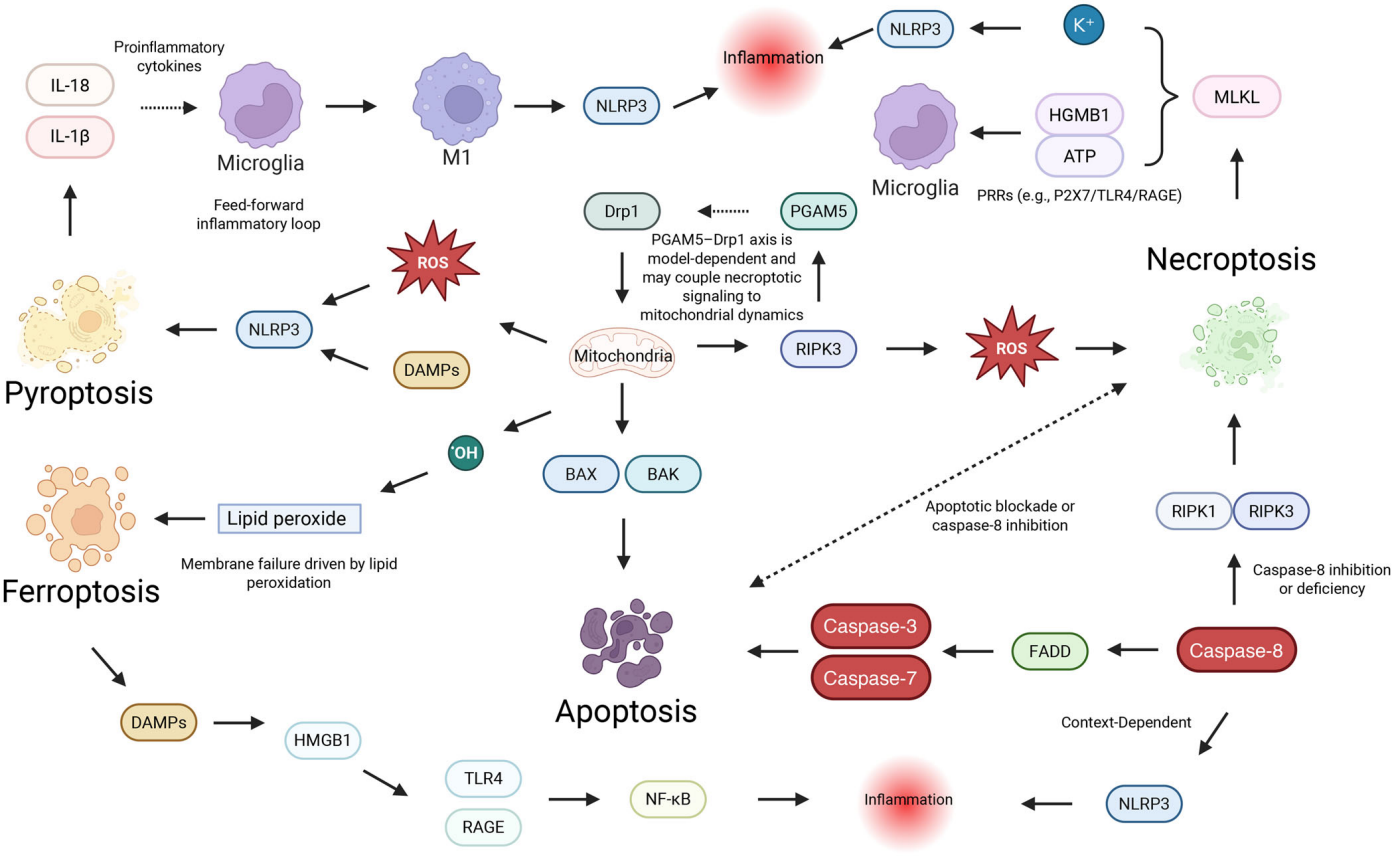
Mitochondrial integration of inflammatory signaling and RCD pathways. This schematic shows how mitochondrial dysfunction links apoptosis, necroptosis, pyroptosis, and ferroptosis in neurodegenerative diseases. Mitochondria connect redox imbalance, inflammatory signaling, lipid peroxidation, and RCD crosstalk. BAX/BAK‐mediated MOMP promotes cytochrome *c* release, apoptosome formation, and Caspase‐9 and Caspase‐3/7 activation. Reduced Caspase‐8 activity favors RIPK1/RIPK3 signaling, MLKL phosphorylation, and necroptotic membrane disruption. The PGAM5 and Drp1 pathway further links necroptosis to mitochondrial fission and ROS accumulation. Mitochondrial ROS and mitochondria‐derived DAMPs activate the NLRP3 inflammasome, leading to Caspase‐1 activation, gasdermin D cleavage, and IL‐1β and IL‐18 release. Mitochondrial redox imbalance and iron‐dependent lipid peroxidation increase ferroptotic vulnerability, while DAMPs such as HMGB1 and ATP activate TLR4, RAGE, and P2×7, sustaining microglial activation and inflammatory amplification. Abbreviations: RCD, regulated cell death; BAX, BCL2‐associated X protein; BAK, BCL2 antagonist/killer; MOMP, mitochondrial outer membrane permeabilization; RIPK1, receptor‐interacting protein kinase 1; RIPK3, receptor‐interacting protein kinase 3; MLKL, mixed lineage kinase domain‐like protein; PGAM5, phosphoglycerate mutase family member 5; Drp1, dynamin‐related protein 1; ROS, reactive oxygen species; DAMPs, danger‐associated molecular patterns; NLRP3, NOD‐like receptor family pyrin domain containing 3; IL‐1β, interleukin‐1β; IL‐18, interleukin‐18; HMGB1, high mobility group box 1; ATP, adenosine triphosphate; TLR4, toll‐like receptor 4; RAGE, receptor for advanced glycation end products; P2×7, purinergic receptor P2×7.

Mitochondria serve as a central hub for multiple RCD pathways because they coordinate oxidative phosphorylation, calcium buffering, redox balance, iron metabolism, and stress signal amplification, thereby linking major death‐related processes summarized in Figure 2 [103, 104]. In intrinsic apoptosis, MOMP leads to the release of cytochrome *c* and other intermembrane proteins, thereby committing cells to apoptotic death [105]. However, in NDs, mitochondrial injury should not be viewed only as an upstream trigger of apoptosis. Mitochondrial dysfunction also creates a biochemical state that amplifies necroptotic signaling, facilitates inflammasome activation, increases susceptibility to ferroptosis, and impairs mitophagy.

In necroptosis, mitochondrial dysfunction mainly acts as an amplifier rather than the sole execution mechanism. RIPK3 can interact with metabolic enzymes and increase ROS production, thereby disrupting mitochondrial membrane potential and reducing ATP synthesis [97]. RIPK3 may also activate phosphoglycerate mutase family member 5 (PGAM5), which promotes dynamin‐related protein 1 (Drp1)‐mediated mitochondrial fission and further propagates metabolic stress [105]. These processes intensify bioenergetic failure and oxidative injury. However, inhibition of mitochondrial fission alone does not fully prevent necroptosis, indicating that mitochondria cooperate with the RIPK1, RIPK3, and MLKL execution machinery rather than replacing it.

Mitochondria also connect ferroptosis and pyroptosis to broader neurodegenerative injury. During ferroptosis, mitochondrial iron overload and impaired iron–sulfur cluster metabolism enhance Fenton chemistry, increase hydroxyl radical generation, and accelerate lipid peroxidation, particularly in membranes enriched with PUFAs. These events damage mitochondrial membrane integrity, elevate ROS levels, and reinforce lipid peroxidation cascades. In pyroptosis, damaged mitochondria release endogenous danger‐associated molecular patterns, including mtDNA and cardiolipin, while mitochondrial ROS act as potent upstream activators of NLRP3 inflammasome assembly [106]. Thus, mitochondrial injury can simultaneously promote oxidative stress, inflammasome activation, and lipid peroxide accumulation, creating a reinforcing loop between metabolic failure and inflammatory cell death.

Mitophagy is a critical regulatory mechanism that limits the spread of mitochondrial death signals. The PTEN‐induced kinase 1 (PINK1)–Parkin pathway facilitates the removal of depolarized mitochondria and supports bioenergetic stability [107]. In addition, receptor‐mediated mitophagy involving BCL2 interacting protein 3 and NIP3‐like protein X (NIX) provides alternative routes for clearing dysfunctional mitochondria under specific cellular stress conditions [108]. Loss of these quality control mechanisms leads to ROS accumulation and increased susceptibility to ferroptotic and necroptotic injury [108]. In PD models, mutations in PINK1 or Parkin are associated with impaired mitochondrial respiration, unstable mitochondrial membrane potential, reduced ATP production, and elevated ROS levels [109, 110]. These findings suggest that restoring mitophagy may reduce the convergence of apoptosis, inflammasome activation, ferroptosis, and inflammatory amplification in NDs [111].

### 3.3 Inflammatory and Immunometabolic Coupling of RCD

Chronic inflammation links pyroptosis, necroptosis, and ferroptosis through reciprocal inflammatory amplification. When immune activation persists, DAMPs, cytokine secretion, oxidative stress, and glial state transitions promote crosstalk among these pathways [112]. Pyroptosis and necroptosis are particularly relevant because both compromise plasma membrane integrity and release inflammatory mediators. GSDMD pores facilitate extracellular release of IL‐1β and IL‐18, whereas MLKL‐mediated membrane disruption releases danger signals such as HMGB1 and ATP [113, 114]. These molecules activate microglial receptors, including TLR4 and purinergic receptor P2×7 (P2×7), stimulate nuclear factor‐κB (NF‐κB) signaling, and promote NLRP3 inflammasome assembly, thereby linking necroptotic membrane rupture to pyroptotic inflammatory signaling [114, 115].

Inflammatory signaling also increases susceptibility to ferroptosis by weakening antioxidant defenses and promoting lipid peroxidation. TNF‐α can activate NF‐κB‐dependent inflammatory signaling and downregulate solute carrier family 7 member 11 (SLC7A11), leading to reduced cystine uptake, impaired glutathione synthesis, and diminished GPX4‐mediated detoxification of lipid peroxides [53]. IFN‐γ similarly suppresses SLC7A11 through the janus kinase–signal transducer and activator of transcription 1 pathway and increases lipid peroxide accumulation by enhancing prooxidative lipid metabolism [116]. Conversely, ferroptosis can intensify inflammation because iron‐dependent lipid peroxidation generates oxidized phospholipids and lipid‐derived danger signals that activate TLR4, receptor for advanced glycation end products, HMGB1‐associated pathways, and NF‐κB signaling [117]. In animal models of AD, PD, and ALS, reduced GPX4 activity often coincides with increased lipid peroxidation and neuroinflammation, whereas interventions that limit ferroptosis can alleviate pathological changes [118, 119]. These findings position ferroptosis as both a metabolic death program and a driver of inflammatory amplification.

Microglial immunometabolism strongly influences how inflammatory and metabolic signals shape RCD progression. Microglia do not passively respond to neuronal debris. They actively adjust glycolysis, oxidative phosphorylation, lipid metabolism, and tricarboxylic acid cycle activity to determine the magnitude and direction of inflammatory cell death signaling [120, 121, 122]. Increased glycolysis supports rapid inflammatory responses and cytokine production, whereas sustained or excessive glycolysis may enhance NLRP3 inflammasome activation, IL‐1β release, and NF‐κB‐mediated inflammatory amplification [120]. By contrast, greater reliance on oxidative phosphorylation and fatty acid metabolism is generally associated with improved microglial surveillance, enhanced phagocytosis, and more efficient clearance of cellular debris, although these effects vary with disease stage and injury setting [122, 123].

These metabolic programs directly shape crosstalk among RCD pathways. Accumulation of tricarboxylic acid cycle intermediates, such as succinate, can stabilize hypoxia‐inducible factor 1α and increase IL‐1β expression, thereby linking metabolic stress to inflammasome activation and pyroptotic signaling [120]. Lipid metabolic remodeling can increase ferroptotic vulnerability by expanding the pool of oxidized phospholipids. Mitochondrial redox imbalance can also reinforce RIPK1 and RIPK3‐associated inflammatory signaling by sustaining TNF‐α production, increasing ROS accumulation, and reducing the efficiency of necrotic debris clearance [116, 118]. This metabolic dependence has important therapeutic implications. In 5xFAD mice, inhibition or deficiency of hexokinase 2 (HK2) enhances microglial clearance of Aβ, reduces plaque burden, and improves cognitive function, partly through increased lipid metabolism and ATP production [122]. Conversely, genetic ablation of HK2 in microglia reduces glycolytic capacity, impairs injury‐induced surveillance and migration, and worsens neuroinflammation and brain injury in ischemic stroke models [123]. More recent evidence suggests that partial HK2 antagonism and complete HK2 loss may have divergent effects during AD progression, with complete loss potentially exacerbating mitochondrial dysfunction and neuroinflammation [124]. Thus, metabolic intervention in microglia is likely to be highly dependent on disease stage, injury type, and the degree of pathway suppression.

### 3.4 cGAS–STING Signaling as a Cross‐Disease DNA‐Sensing Hub in Neurodegeneration

Evidence from disease models supports the involvement of cyclic GMP–AMP synthase‐stimulator of interferon genes (cGAS–STING) signaling in neurodegeneration. In AD, cellular stress caused by Aβ, tau, and apolipoprotein E (APOE) ε4 promotes cytoplasmic DNA accumulation in microglia and neurons and is associated with increased cGAS and STING expression in plaque enriched regions. STING activation interacts with inflammasome priming, Type I interferon signaling, and synaptic remodeling, contributing to neuroinflammation and memory impairment. Experimental STING inhibition reduces gliosis and amyloid burden in animal models, suggesting that this pathway connects innate immune activation with core AD pathological changes [125]. A related inflammatory pattern has been observed in α‐synucleinopathy models, where α‐syn aggregation promotes DNA damage and stimulates STING‐mediated responses, particularly in microglia. The effect of STING deficiency in these models appears to vary with disease stage, baseline inflammatory activity, and experimental paradigm, indicating that cGAS–STING signaling modifies cellular vulnerability rather than acting through a single linear mechanism [125].

Motor neuron disease and polyglutamine disease models further extend this link between mitochondrial injury, DNA release, and innate immune activation. In ALS, TAR DNA‐binding protein 43 (TDP‐43) and SOD1 driven mitochondrial injury promotes the release of mtDNA and ribonucleic acid (RNA):DNA hybrids, leading to cGAS–STING activation in motor neurons and adjacent spinal cord tissues [126, 127]. This activation can propagate inflammatory signals to neighboring cells. In HD, mutant huntingtin (mHTT)‐associated mitochondrial instability promotes mtDNA release and activates cGAS, STING, and interferon regulatory factor 3 signaling, thereby contributing to neuroinflammation. Melatonin treatment appears to reduce downstream inflammatory signaling in this pathway [128].

## 4 Dysregulation of Cell Death Mechanisms in Major NDs

Although multiple forms of RCD have been documented across NDs, their activation conditions, dominant signaling pathways, and functional consequences are not uniform. Rather, they differ according to pathogenic protein burden, selective cellular vulnerability, mitochondrial and lysosomal stress, and the local inflammatory milieu. These factors together shape the pattern of cell death dysregulation in each disorder [129].

In diseases marked mainly by Aβ or tau deposition, mitochondrial dysfunction, lipid peroxidation, and inflammasome‐associated signaling often act together to activate multiple cell death pathways [53, 130]. In this setting, the efficiency of glial clearance strongly influences the inflammatory consequences of neuronal injury. Inefficient removal of apoptotic neurons, damaged mitochondria, oxidized lipids, and protein aggregate‐associated debris allows DAMPs to persist and sustains microglial activation [131, 132]. Although defective clearance is not a distinct RCD modality, it can intensify apoptosis‐associated inflammation and promote secondary activation of inflammatory cell death pathways [133]. By contrast, in disorders dominated by synaptic dysfunction or motor neuron degeneration, excitotoxicity, impairment of the autophagy lysosomal system, and secondary inflammatory responses may have a more prominent role in cellular injury [134, 135].

The pathological significance of each death program also varies by disease stage and cell type. In some settings, RCD directly contributes to neuronal loss. In others, it promotes disease progression through sublethal stress signaling, inflammatory amplification, or impaired clearance of dying cells [136, 137]. These differences help explain why the same molecular marker may indicate terminal cell death in one disease state but inflammatory priming or cellular stress in another. They also determine whether defective clearance acts mainly as a downstream consequence of neuronal injury or as a sustained amplifier of neuroinflammation, particularly in AD [138, 139]. This heterogeneity contributes to the clinical and pathological diversity of NDs and supports the need for therapeutic strategies that account for disease stage, cell type, and inflammatory state.

**FIGURE 3:**
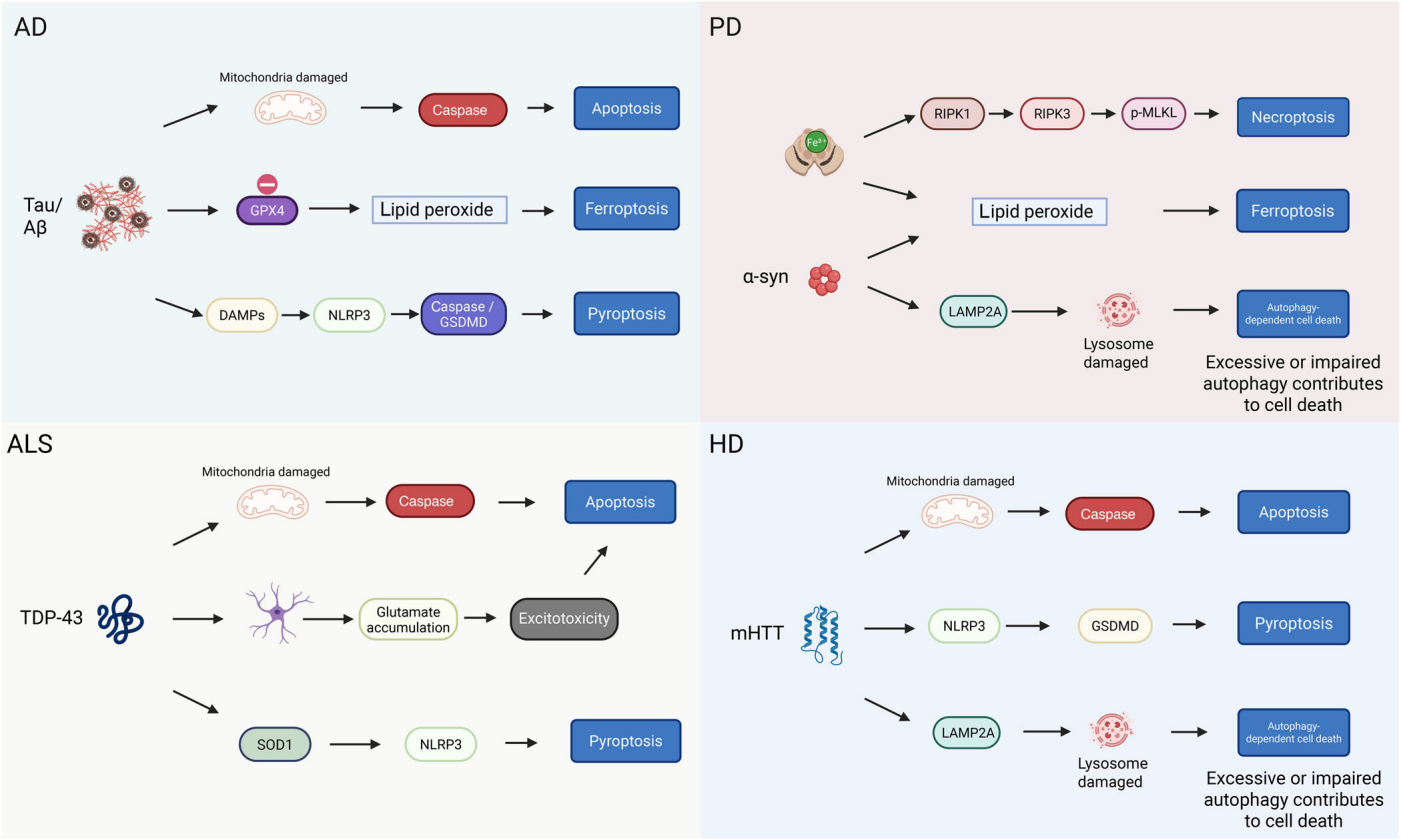
Disease‐specific activation of RCD pathways in major neurodegenerative disorders. This schematic summarizes the major RCDpathways activated in AD, PD, amyotrophic lateral sclerosis (ALS), and Huntington's disease (HD), with emphasis on disease‐related upstream stressors and downstream death signaling. In AD, Aβ deposition and tau pathology promote mitochondrial dysfunction, caspase activation, lipid peroxidation, NLRP3 inflammasome signaling, and autophagy lysosomal impairment, contributing to apoptosis, ferroptosis, pyroptotic inflammation, and proteotoxic stress. In PD, α‐syn aggregation, mitochondrial Complex I dysfunction, iron accumulation, and lysosomal impairment increase dopaminergic neuronal vulnerability through ferroptosis, RIPK1/RIPK3/MLKL‐mediated necroptosis, and autophagy‐related injury. In ALS, TDP‐43 and FUS pathology, C9orf72‐related RNA toxicity, ER stress, excitotoxicity, and astrocytic glutamate transport failure converge on motor neuron degeneration through caspase‐mediated apoptosis, inflammasome activation, pyroptotic signaling, and impaired proteostasis. In HD, mHTT disrupts mitochondrial dynamics, autophagy lysosomal function, glutamate homeostasis, and glial inflammatory signaling, thereby promoting apoptosis, pyroptosis, ferroptosis‐related oxidative injury, and autophagy‐dependent neuronal damage. Together, these disease‐specific patterns indicate that RCD activation differs across neurodegenerative disorders but commonly involves mitochondrial injury, proteostasis failure, lipid redox imbalance, lysosomal dysfunction, and neuroinflammatory amplification. Abbreviations: AD, Alzheimer's disease; PD, Parkinson's disease; ALS, amyotrophic lateral sclerosis; HD, Huntington's disease; RCD, regulated cell death; Aβ, amyloid β; α‐syn, α‐synuclein; GPX4, glutathione peroxidase 4; DAMPs, danger‐associated molecular patterns; NLRP3, NOD‐like receptor family pyrin domain containing 3; GSDMD, gasdermin D; LAMP2A, lysosome‐associated membrane protein Type 2A; RIPK1, receptor‐interacting protein kinase 1; RIPK3, receptor‐interacting protein kinase 3; MLKL, mixed lineage kinase domain‐like protein; p‐MLKL, phosphorylated MLKL; TDP‐43, TAR DNA‐binding protein 43; SOD1, superoxide dismutase 1; mHTT, mutant huntingtin; FUS, fused in sarcoma; C9orf72, Chromosome 9 open reading frame 72; RNA, ribonucleic acid; ER, endoplasmic reticulum; PANTHOS, plaque‐associated neuronal death with highly organized structure.

The following sections examine the major dysregulated RCD modalities and their principal molecular pathways in AD, PD, ALS, and HD. A comparative overview of these dysregulation patterns is shown in Figure 3.

### 4.1 Alzheimer's Disease

AD accounts for approximately 60–80% of dementia cases and is clinically characterized by progressive impairment of memory and executive function [140]. Its core pathological features include Aβ deposition and aberrant tau aggregation, which sustain cellular stress by disrupting Ca^2^^+^ homeostasis, impairing mitochondrial respiratory chain activity, and overloading the autophagy lysosomal system. Together, these changes promote coordinated dysregulation of cell death pathways, with caspase activation, mitochondrial dysfunction, and persistent inflammation acting as major pathological drivers [141, 142].

At early disease stages, impaired mitochondrial quality control and reduced lysosomal acidification facilitate the accumulation of damaged mitochondria and toxic proteins. Impaired phagocytic clearance by glial cells further amplifies these changes, creating a sustained proinflammatory environment that accelerates neuronal loss and cognitive decline [143, 144]. Inadequate removal of apoptotic neurons, synaptic debris, damaged mitochondria, and Aβ‐associated cellular remnants leads to persistent microglial activation and amplification of neuroinflammatory responses.

#### 4.1.1 Aβ‐ and Tau‐Associated Cell Death Signaling

In AD, soluble Aβ oligomers and pathological tau aggregates rarely induce synchronized apoptosis at a large scale. Instead, they promote persistent and spatially dispersed death signaling, with caspase activation serving as a major feature. Neuropathological analyses have detected activated Caspase‐3 in hippocampal and cortical neurons from AD brains, whereas such activation is rarely observed in age matched controls. Apoptotic like morphology remains relatively uncommon, suggesting that caspase activation in AD more often reflects sustained stress‐related pathology rather than a single terminal clearance event [145]. Subcellular fractionation further showed enrichment of Caspase‐3 in postsynaptic density regions, with Procaspase‐3 increased by approximately 39% in synaptosomal fractions and 84% in postsynaptic density fractions, while activated Caspase‐3 increased by about 147%. These findings indicate direct involvement of Caspase‐3 in synaptic structural and functional disruption, beyond its role as a marker of cell death [146].

Current evidence indicates that Aβ oligomers induce intrinsic mitochondrial stress and interact with endoplasmic reticulum stress, Ca^2^^+^ dyshomeostasis, and ROS imbalance to accelerate AD progression. In LAN5 neuroblastoma cells, 20 µM Aβ_42_ oligomers show greater toxicity than fibrillar aggregates at equivalent concentrations and primarily induce apoptosis through a Caspase‐9‐dependent intrinsic pathway. Cytochrome *c* is detected only in the cytosolic fraction of oligomer treated cells, indicating MOMP [147]. In APP/PS1 mice, mitochondria‐targeted Ca^2^^+^ ratio imaging using YC3.6 showed that neurons with a baseline yellow fluorescent protein/cyan fluorescent protein ratio of 1.4 or higher, corresponding to approximately 3.1 µM mitochondrial free Ca^2^^+^, disappeared within 24 h, whereas neurons with ratios of 1.4 or lower remained stable. Mitochondrial Ca^2^^+^ overload at approximately 1.6–1.7 µM was associated with reduced membrane potential, mitochondrial permeability transition pore opening, and Caspase‐3/7 activation in primary cortical neurons [148, 149]. In addition, Aβ_1__−__42_ induces an unfolded protein response. Phosphorylated protein kinase R‐like endoplasmic reticulum kinase (PERK) and eukaryotic initiation factor 2α (eIF2α) are upregulated within 6 h in SK‐N‐SH neurons, whereas inositol‐requiring enzyme 1α (IRE1α) and activating transcription factor 6 (ATF6) show no significant changes within 12 h. These data suggest that the PERK and eIF2α axis functions as an early and selective stress response pathway [150].

APP is also an important caspase substrate. Caspase‐3 and Caspase‐8 cleave APP at the C‐terminal Asp664 site, generating the cytotoxic intracellular fragment C31, which is associated with synaptic degeneration [151, 152]. In transgenic mice, mutation of this site, such as D664A, significantly reduces Aβ‐induced synaptic injury and dendritic spine loss, while improving synaptic density and cognitive performance. This evidence supports a pathogenic role for caspase‐mediated APP cleavage in AD [151].

Beyond their apoptotic functions, activated caspases can act as pathological processing enzymes that worsen AD‐related protein aggregation. Caspase‐3 cleaves tau at Asp421, generating a C‐terminal truncated isoform with accelerated aggregation kinetics and greater propensity to form paired helical filaments [153]. Other caspases, including Caspase‐6, cleave tau at D402 and D421 and generate multiple truncated tau species closely linked to AD pathology [154]. Caspase‐3 truncated tau, also known as TauC3, is significantly elevated in AD brains and nearly doubles in the hippocampus of aged mice, indicating an association with disease progression and aging [155]. These truncated tau species show increased aggregation propensity and induce mitochondrial fragmentation, dysfunction, and oxidative stress, collectively contributing to cellular senescence‐associated toxicity and cognitive decline [156].

Under physiological conditions, Caspase‐8 suppresses the RIPK1/RIPK3 axis and prevents aberrant necroptosis. In AD, however, this inhibition is reduced, resulting in activation of necroptotic signaling [157]. Analyses of AD brain tissue show marked increases in the expression and phosphorylation of RIPK1, RIPK3, and MLKL. Signal intensity rises with Braak stage and negatively correlates with cognitive performance measured by mini‐mental state examination, indicating a close association between necroptotic signaling and cognitive decline [142]. In APP/PS1 mice, pharmacological and genetic inhibition of RIPK1 alleviates neuroinflammation and neuronal injury, reduces Iba1‐positive microglia, and improves spatial learning and memory [158]. These findings implicate excessive activation of the RIPK1–RIPK3–MLKL pathway in AD‐related neuronal injury and suggest that preservation of Caspase‐8 regulatory activity may limit inflammatory cell death.

Aβ deposition and tau aggregation also enhance microglial inflammasome activation, further amplifying neuroinflammation [54]. This activation is usually accompanied by increased levels of cleaved Caspase‐1, mature IL‐1β, and GSDMD. In APP/PS1 and tauopathy models, deletion of NLRP3 or ASC significantly reduces Aβ and tau burden, attenuates inflammatory responses, and partially restores long‐term potentiation (LTP) amplitude, spatial learning, and memory. These findings support a major role for NLRP3/ASC inflammasome‐mediated chronic inflammation in synaptic dysfunction and AD progression [53, 54].

#### 4.1.2 Mitochondrial and Lysosomal Dysfunction

The combined disruption of mitochondrial and lysosomal function is a defining feature of AD progression. Brain tissues from individuals with AD and classical transgenic mouse models, including APP/PS1 and 5xFAD mice, show characteristic mitochondrial abnormalities, including reduced oxidative phosphorylation efficiency, impaired activity of respiratory chain Complexes I and IV, loss of mitochondrial ΔΨm, oxidative mtDNA damage, and abnormal ROS accumulation [141, 158, 159]. Transmission electron microscopy has revealed widespread cristae disruption, matrix rarefaction, and focal swelling in cortical and hippocampal neurons, suggesting that mitochondrial degeneration can precede overt neuronal loss.

These structural and functional alterations cause energy deficiency and promote MOMP, followed by cytochrome *c* release and activation of the Caspase‐9/3 cascade. Impaired electron transport and increased electron leakage also facilitate lipid peroxidation, thereby increasing ferroptotic vulnerability [141]. In AD, reduced cytochrome oxidase activity in the posterior cingulate cortex correlates with regional glucose hypometabolism and cognitive decline, indicating that metabolic impairment is an early disease feature. In APP/PS1 mice and Aβ‐exposed SH‐SY5Y cells, decreases in ΔΨm, oxygen consumption rate, and ATP levels occur before neuronal loss and plaque formation, supporting mitochondrial dysfunction as an early pathogenic event in AD [158].

Aβ and tau are major disruptors of mitochondrial homeostasis. Aβ/APP accumulates abnormally at mitochondrial protein import channels and forms translocation arrest complexes. In AD brain tissue, nonglycosylated full‐length APP and its C‐terminal fragments accumulate on mitochondrial translocase complexes and form stable complexes with translocase of the outer mitochondrial membrane 40 (TOM40) and TOM40/translocase of the inner mitochondrial membrane 23, with abundance increasing alongside disease severity (*p* < 0.05, BN‐PAGE, *n* = 3). This process is associated with a 60–80% reduction in the import efficiency of nuclear encoded respiratory proteins such as cytochrome *c* oxidase (COX) IV and Vb [160, 161]. As a result, Complex IV activity declines by 40–60%, and H_2_O_2_ levels increase twofold to threefold, indicating combined impairment of bioenergetic and redox homeostasis [161].

Free Aβ_1__−__40_/_1__−__42_ peptides are also actively imported into mitochondria through the TOM complex, independently of membrane potential. Preincubation with antibodies against TOM20, TOM40, or TOM70 reduces mitochondrial Aβ import by 40–70% (TOM20/40: *p* < 0.05; TOM70: *p* < 0.01), whereas voltage‐dependent anion channel inhibition or CsA treatment does not affect import, supporting specificity for the TOM40 channel [162]. Immunoelectron microscopy shows that approximately 75% of Aβ42 localizes to inner mitochondrial cristae and colocalizes with COX subunits, indicating direct interference with the electron transport chain [162]. Aβ further enhances mitochondrial toxicity through direct protein interactions. Lustbader et al. showed that Aβ binds mitochondrial alcohol dehydrogenase Aβ binding alcohol dehydrogenase (ABAD), occupies its NAD^+^ binding pocket, alters protein conformation, increases ROS production, disrupts membrane permeability, elevates Ca^2^^+^ levels, and activates apoptotic signaling [163]. Pharmacological or peptide‐mediated disruption of ABAD–Aβ interaction restores ΔΨm, reduces ROS, and improves learning and memory in AD models, identifying this complex as an important mediator of mitochondrial toxicity [164, 165].

Tau further amplifies mitochondrial injury by disturbing mitochondrial dynamics. Increased expression and phosphorylation of the mitochondrial fission protein Drp1 are consistently observed in AD brains and transgenic models, leading to excessive mitochondrial fragmentation. In frontal cortex tissue across Braak Stages I–VI, Drp1 levels are elevated from the earliest stage (Braak I/II: *p* < 0.005; III/IV: *p* < 0.02; V/VI: *p* < 0.002) [166]. In APP and APP/PS1 mice, Drp1 phosphorylation at Ser616 is markedly increased. In 3xTg‐AD mice, this change is localized to hippocampal mitochondria, increasing the proportion of p‐Drp1 (S616)‐positive mitochondria from 19.5 to 53% (*p* < 0.05, *n* = 3) [167]. These changes shorten average mitochondrial length and reduce synaptic mitochondrial density, thereby compromising synaptic energy supply.

Coimmunoprecipitation confirms abnormal interactions between Aβ and Drp1 in AD cortex, with Drp1 immunoprecipitates containing both monomeric and oligomeric Aβ and increasing in parallel with Braak stage. This suggests that Aβ and Drp1 complex formation promotes mitochondrial Drp1 recruitment and amplifies mitochondrial stress [166]. In APP/PS1 mice, abnormal Drp1 recruitment is associated with increased mitochondrial ROS, a 30–40% reduction in membrane potential, ATP depletion, cytochrome *c* release, and Caspase‐9/3 activation, consistent with mitochondria‐dependent apoptosis. Treatment with the Drp1 inhibitor Mdivi‐1 reduces mitochondrial Drp1 localization, partially restores mitochondrial morphology, mitigates oxidative stress, and improves spatial memory in the Morris water maze (*p* < 0.05), supporting the therapeutic potential of limiting excessive mitochondrial fission [168]. Reduced synaptic mitochondria and increased oxidative stress also impair Ca^2^^+^ buffering, thereby increasing neuronal vulnerability to necroptosis and ferroptosis [158].

Impaired mitophagy is another major contributor to AD‐associated cell death. Postmortem hippocampal tissues and induced pluripotent stem cell (iPSC)‐derived neurons from patients with AD show a 30–50% reduction in basal mitophagy compared with age matched controls, reduced TOMM20 and LAMP2 colocalization (*p* < 0.001), and fewer mitophagy like events under electron microscopy (*p* = 0.005). PINK1, Parkin, phosphorylated TBK1, and ULK1 levels are also decreased, indicating defective autophagy initiation [143]. Accumulated damaged mitochondria increase ROS production and release mtDNA and other DAMPs, thereby intensifying inflammation and cell death signaling [169]. Pharmacological enhancement of mitophagy restores mitochondrial morphology, reduces Aβ and hyperphosphorylated tau accumulation, and reverses cognitive deficits [143]. In APP/PS1 mice, daily oral gavage with urolithin A (200 mg/kg/day) or actinonin (30 mg/kg/day) from 6 months of age for 2 months significantly increased mitophagy like events and reduced damaged mitochondria in hippocampal neurons compared with vehicle treated APP/PS1 mice [143].

Lysosomal and autophagic impairment further promotes Aβ and APP‐βCTF accumulation within deacidified autolysosomes, producing enlarged vacuolar structures. Multimodal imaging and correlative electron microscopy across several AD mouse models show that autolysosomal acidification declines before plaque deposition, leading to the PANTHOS pathological phenotype. Quantitative analysis identifies PANTHOS neurons as a major source of amyloid plaques in APP models [82, 170, 171, 172]. This autophagic blockade disrupts energy metabolism and proteostasis, triggers ADCD, and increases Aβ and tau aggregation, thereby reinforcing neurotoxicity.

Oxidative stress and Aβ conformational changes can also induce LMP, releasing cathepsins B and D into the cytosol. These cathepsins activate Caspase‐8 and the NLRP3 inflammasome, thereby promoting pyroptotic or necroptotic signaling [173, 174, 175]. The severity of LMP influences the mode of cell death: mild permeabilization tends to favor apoptosis, whereas severe permeabilization promotes cathepsin‐dependent necroptosis and pyroptosis. Because tau clearance depends heavily on the autophagy lysosomal pathway, dysfunction of this system causes persistent tau aggregate accumulation and further increases lysosomal stress [176]. Enhancing TFEB activity or improving lysosomal function promotes tau degradation, reduces neuroinflammation, and rescues synaptic structure and cognition [177, 178].

Overall, dysfunction of the mitochondrial and lysosomal systems provides a major mechanistic link between metabolic failure, toxic protein accumulation, inflammation, and RCD in AD. Mitochondrial damage drives energy failure and oxidative stress, whereas autophagic and lysosomal defects impair protein and organelle clearance. LMP, cathepsin release, inflammasome activation, and necroptotic signaling further connect organelle dysfunction with inflammatory cell death, contributing to progressive and difficult to reverse neurodegeneration.

#### 4.1.3 Glial Clearance Dysfunction and Chronic Inflammation–Mediated Neuronal Death

Under physiological conditions, the inflammatory outcome of neuronal death depends partly on whether dying cells and cellular debris are cleared in a timely manner. Efferocytosis refers specifically to the phagocytic removal of apoptotic cells and their derivatives by microglia and other phagocytes. It is not a form of RCD, but a downstream clearance process that helps preserve the largely noninflammatory nature of apoptosis. More broadly, glial phagocytic clearance also removes synaptic debris, damaged mitochondria, oxidized lipids, extracellular protein aggregates, and other injury‐associated materials. Efficient clearance limits extracellular accumulation of mtDNA, oxidized lipids, ATP, HMGB1, and other DAMPs, thereby restricting inflammatory signal propagation and promoting tissue repair [179, 180, 181, 182]. In AD, however, this clearance capacity progressively declines. As apoptotic neurons, synaptic debris, damaged mitochondria, and Aβ‐associated cellular remnants persist in the brain microenvironment, local neuronal injury is converted into sustained microglial activation and chronic neuroinflammation.

In APP/PS1 mice, microglia surrounding Aβ deposits show reduced lysosomal acidification and decreased Rab7 and LAMP1 fusion efficiency, indicating defective maturation of the phagosome lysosome pathway [183, 184, 185, 186]. In primary microglia exposed to Aβ_1__−__42_, Aβ disrupts TFEB localization by inhibiting its nuclear translocation. As Aβ_1__−__42_ concentration increases, nuclear TFEB levels decrease in a dose‐dependent manner, with an approximately 20% reduction at 5 µM and up to 80% reduction at 10 µM. Impaired TFEB function leads to lysosomal disturbance, as shown by insufficient lysosomal acidification. Under resting conditions, lysosomal pH in microglia is approximately 5.96. After Aβ exposure, only transient acidification occurs, followed rapidly by a return to near neutral pH. In parallel, osteopetrosis‐associated transmembrane protein 1 expression is significantly reduced, whereas LAMP1 levels continue to rise, suggesting accumulation of dysfunctional lysosomes rather than effective lysosomal biogenesis. These findings indicate that AD microglia can internalize Aβ and cellular debris but fail to degrade them efficiently, prolonging phagosomal retention and inflammatory activation [187]. Consistently, AD brain tissues show reduced lysosomal numbers and accumulation of immature phagocytic vacuoles [188].

Defective microglial clearance is further aggravated by impaired LC3‐associated phagocytosis and LC3‐associated endocytosis (LANDO) activity. Under high Aβ burden, reduced expression of essential LANDO regulators, including Rubicon, Atg5, and Atg7, limits LC3 lipidation and recruitment to phagosomal membranes, disrupting maturation and degradation of Aβ containing compartments. Transcriptomic studies further suggest impairment of phagocytic, lysosomal, and LC3‐associated clearance programs in AD brain tissue, closely linked to persistent NLRP3 inflammasome activation [180, 181, 189, 190, 191]. Thus, impaired clearance does not execute neuronal death directly. Instead, it establishes a debris driven pathological circuit in which Aβ retention, lysosomal dysfunction, inflammasome activation, and cytokine release increase neuronal susceptibility to RCD.

With chronic inflammatory stimulation, microglia gradually shift from a homeostatic and clearance competent phenotype toward a proinflammatory state with reduced phagocytic capacity. Marschallinger and colleagues identified lipid droplet accumulating microglia (LDAM) during aging and inflammation, which display marked functional impairment and a proinflammatory transcriptional profile [192]. Transmission electron microscopy and Boron‐dipyrromethene (BODIPY)/coherent anti‐Stokes Raman scattering imaging showed that BODIPY‐positive microglia increase from approximately 12% in young mice to 52% in the aged hippocampus, with marked increases in droplet number and volume per cell. Similar Perilipin2‐positive LDAM were detected in the aged human hippocampus, suggesting cross species conservation. Transcriptomic profiling revealed that LDAM downregulate genes related to phagosome maturation, lysosomal function, and metabolic homeostasis, whereas genes associated with oxidative stress and inflammation remain persistently upregulated. Functionally, only 2.1% of Zymosan‐positive and BODIPY‐positive microglia show phagocytic activity, compared with 24.3% of Zymosan‐positive and BODIPY‐negative microglia. LDAM also display approximately twofold higher ROS levels and constitutive overproduction of IL‐1β, TNF‐α, IL‐6, CCL3, CCL4, and CXCL10 [192]. These findings provide a plausible mechanism by which aging‐associated microglial metabolic dysfunction may reduce clearance capacity while sustaining inflammatory priming in AD.

Astrocytes are also reprogrammed during this process. Under normal conditions, astrocytes regulate synaptic homeostasis, provide metabolic support, clear extracellular glutamate, and participate in Aβ metabolism through APOE‐dependent lipoprotein transport. In the inflammatory milieu of AD, activated microglia release IL‐1α, TNF‐α, and C1q, driving astrocytic transformation toward a neurotoxic A1 reactive phenotype [193, 194]. A1 astrocytes upregulate complement C3 and Serping1, lose neurotrophic and axon supportive functions, disrupt oligodendrocyte‐mediated myelin maintenance, and directly induce neuronal death. C3‐positive A1 astrocytes are enriched in regions of Aβ deposition and neuronal loss in AD brains, implicating them in disease progression [193, 195]. Through excessive complement activation, reactive astrocytes also contribute to aberrant synaptic elimination. A1 astrocyte‐derived C3 enhances synaptic complement tagging and promotes inappropriate engulfment of structurally intact synapses by CR3‐positive microglia [196, 197]. In APP/PS1 mice, C3 decorated synapses strongly colocalize with CR3‐positive microglial processes and are accompanied by reduced PSD‐95 and synaptophysin levels. Deletion of C3 (APP/PS1; C3−/−) or CR3 (ITGAM/CD11b−/−) partially restores synaptic density, improves LTP, and enhances spatial learning, confirming complement driven aberrant synaptic pruning as a major mechanism of early synaptic pathology [196, 198].

Astrocytic clearance mechanisms are also impaired in AD and may contribute to persistence of extracellular protein aggregates and synaptic debris. Aβ and inflammatory stimuli disrupt lysosomal activity and reduce cathepsin D activity, compromising astrocytic degradation of extracellular proteins and synaptic debris [199]. Moreover, MerTK and MEGF10, key mediators of astrocytic phagocytosis, are downregulated in Aβ treated primary astrocytes and in APP/PS1 and 5xFAD models. This impairment reduces clearance of synaptic remnants and extracellular tau, thereby facilitating tau propagation and neurofibrillary tangle spread [200, 201].

Inflammation and metabolic stress may further weaken neural repair capacity. In intracerebroventricular streptozotocin‐induced sporadic AD rat models, both the number and average diameter of neurospheres formed by neural stem cells are significantly reduced, accompanied by disorganized morphology and decreased Seladin‐1 expression. Triiodothyronine treatment restores neurosphere formation and Seladin‐1 levels (*p* < 0.05), suggesting impaired neural stem cell renewal and proliferation [202]. In chronic or intermittent hypoxia models, hippocampal neurons show reduced neuronal density, disrupted Nissl bodies, and abnormal neuronal morphology, particularly in CA1, with partial resistance in CA3 [203]. NeuN immunoreactivity also shows significant loss of mature neurons under chronic hypoxic or hypoperfusion conditions, accompanied by sustained microglial and astrocytic activation [204, 205, 206]. These findings indicate that inflammatory and metabolic stress can impair neuronal maintenance and repair, although they should be interpreted as supportive evidence rather than direct evidence of glial clearance failure.

As AD progresses, inadequate clearance of apoptotic and necrotic neurons as well as synaptic debris leads to chronic accumulation of mtDNA and oxidized lipids, which serve as endogenous stimuli for inflammatory signaling. Persistent activation of cGAS–STING and NLRP3 inflammasome pathways in microglia can transform transient inflammatory responses into chronic neuroinflammation [207, 208]. Activation of the mtDNA and cGAS–STING axis is associated with sustained upregulation of Type I interferon and proinflammatory cytokines, which are linked to neuronal dysfunction and cognitive decline [209]. Concurrently, NLRP3 inflammasome‐dependent immunometabolic reprogramming further impairs microglial phagocytosis while maintaining chronic IL‐1β release [210, 211]. Thus, glial clearance dysfunction in AD should be understood not as an independent cell death pathway, but as a debris driven amplifier of RCD‐associated inflammation that increases neuronal vulnerability to apoptosis, necroptosis, pyroptosis, and ferroptosis.

### 4.2 Parkinson's Disease

PD is a progressive neurodegenerative disorder characterized by selective loss of dopaminergic neurons in the substantia nigra pars compacta and abnormal aggregation of α‐syn within Lewy bodies [212, 213]. Genetic studies have shown that synuclein alpha duplications and point mutations, including A53T, E46K, and A30P, are strongly associated with familial PD. These mutations alter the conformational stability of α‐syn and promote its conversion from soluble monomers into cytotoxic oligomers and fibrillar aggregates, which can spread through neural circuits in a prion like manner [214].

Misfolded α‐syn and environmental toxins such as MPTP and rotenone inhibit mitochondrial Complex I activity, leading to reduced mitochondrial membrane potential, impaired ATP production, and excessive ROS accumulation. These changes increase metabolic and oxidative stress in dopaminergic neurons, which are intrinsically vulnerable because of their high energy demand and autonomous pacemaking activity [212, 215]. Degeneration of nigral dopaminergic neurons reduces striatal dopamine and disrupts basal ganglia circuitry, producing the cardinal motor manifestations of PD, including bradykinesia, resting tremor, and rigidity.

Nonmotor symptoms, including hyposmia, gastrointestinal dysmotility, and rapid eye movement sleep behavior disorder, often precede motor symptoms by several years. This temporal pattern suggests that α‐syn pathology may begin outside the substantia nigra and spread through interconnected central and peripheral neural pathways [212]. Beyond mitochondrial dysfunction, PD brains show abnormal iron accumulation, increased lipid peroxidation, and disruption of the GPX4 and glutathione antioxidant system, supporting the involvement of ferroptosis‐related injury [215, 216]. Aggregated α‐syn can also act as an endogenous inflammatory trigger, activating the NLRP3 inflammasome in microglia and promoting pyroptotic signaling [217, 218].

Overall, PD pathogenesis reflects the convergence of α‐syn aggregation, mitochondrial dysfunction, iron dyshomeostasis, ferroptosis‐related lipid injury, and chronic neuroinflammation, rather than a single initiating event. These interacting processes jointly contribute to progressive dopaminergic neuron loss and clinical deterioration.

#### 4.2.1 Mitochondrial Complex I Inhibition, α‐Syn Toxicity, and Defects in Mitochondrial Quality Control

Mitochondrial Complex I dysfunction is recognized as an early driver of dopaminergic neurodegeneration in PD. Neuropathological studies have shown substantially reduced Complex I enzymatic activity in the substantia nigra pars compacta of affected individuals, indicating that impaired oxidative phosphorylation and energy metabolism can occur from early disease stages [219]. In the classical MPTP model, the active metabolite MPP^+^ selectively enters dopaminergic neurons and directly inhibits Complex I, rapidly causing loss of mitochondrial membrane potential, restricted ATP synthesis, and persistent ROS overproduction. These events increase metabolic and oxidative stress in vulnerable neurons [220]. Under these conditions, the intrinsic mitochondrial apoptotic pathway is activated, as shown by cytochrome *c* release, Apaf‐1 apoptosome formation, and sequential activation of Caspase‐9 and Caspase‐3.

Complex I inhibition also increases neuronal sensitivity to necroptosis and ferroptosis. In cybrid cell models containing mtDNA from patients with PD, Complex I activity is reduced by approximately 20% compared with controls, accompanied by elevated ROS production and greater vulnerability to MPP^+^‐induced RCD [72]. In subacute MPTP‐treated C57BL/6 mice, RIPK1, RIPK3, and phosphorylated MLKL protein levels are significantly increased in both the substantia nigra and striatum, indicating early necroptotic activation. MPTP administration alone causes approximately 60% loss of TH‐positive dopaminergic neurons in the substantia nigra, whereas intracerebroventricular delivery of necrostatin‐1 reduces neuronal loss to about 27% and improves striatal TH‐positive fiber density. These findings show that necroptosis inhibition can markedly attenuate MPTP‐induced neurodegeneration and motor deficits [222, 223].

MPP^+^ exposure also markedly elevates mitochondrial ROS levels and activates the intrinsic apoptotic cascade, as reflected by Caspase‐9 and Caspase‐3 cleavage. Restoration of mitochondrial electron transport through overexpression of yeast NADH‐quinone oxidoreductase normalizes ROS levels to near control values and largely suppresses caspase‐dependent apoptotic signaling, highlighting the central role of mitochondrial redox imbalance in MPP^+^‐induced dopaminergic neurodegeneration [224, 225, 226]. Together, these data indicate that mitochondrial dysfunction initiates classical apoptosis while also increasing neuronal vulnerability to necroptosis and iron‐dependent oxidative injury.

Pathological α‐syn aggregation further exacerbates mitochondrial damage. Aggregated α‐syn associates directly with mitochondria, disrupts respiratory chain function, impairs ATP synthesis, and increases oxidative stress and apoptotic signaling. In mouse models induced by α‐syn preformed fibrils, pronounced mitochondrial impairment is observed, and interventions targeting oxidative stress pathways partially alleviate α‐syn‐associated pathology and motor deficits, supporting the pathogenic role of α‐syn‐mediated mitochondrial dysfunction [227, 228].

Study demonstrated that α‐syn directly binds the mitochondrial Complex I subunit NADH:ubiquinone oxidoreductase core subunit S3, thereby inhibiting Complex I activity and reducing oxidative phosphorylation efficiency. Further studies confirmed a strong correlation among α‐syn accumulation, reduced Complex I activity, and ATP deficiency [229]. Consistently, α‐syn‐induced mitochondrial dysfunction is accompanied by excessive ROS production, severe energy imbalance, and oxidative stress [230]. Moreover, α‐syn fibrils can physically interact with mitochondria, induce marked mitochondrial stress responses, and accelerate neuronal death, providing direct evidence of mitochondrial toxicity [231].

Genetic mutations associated with familial PD further increase mitochondrial vulnerability and compromise quality control mechanisms. Loss of function mutations in PINK1 or Parkin impair mitophagy, prevent timely elimination of damaged mitochondria, and lead to persistent ROS accumulation, mtDNA leakage, and Bax translocation. These events converge on apoptotic, necroptotic, and ferroptotic signaling cascades [232, 233]. Parkinsonism‐associated deglycase (DJ‐1) mutations also weaken mitochondrial antioxidant defenses by inhibiting Nrf2 nuclear translocation and downregulating downstream effectors such as heme oxygenase‐1 (HO‐1) and NAD(P)H quinone dehydrogenase 1 (NQO1). In DJ‐1 deficient models, including mice, Drosophila, and iPSC‐derived neurons, reduced mitochondrial membrane potential, increased oxidative damage, and elevated Caspase‐3‐mediated apoptosis are consistently observed [234, 235].

Collectively, defects in mitochondrial quality control genes lower the antioxidant threshold of dopaminergic neurons, making them more susceptible to Complex I inhibition and α‐syn aggregation‐associated stress. This vulnerability promotes progressive neuronal death and PD progression.

#### 4.2.2 Ferroptosis and the Selective Vulnerability of Dopaminergic Neurons

Evidence from imaging, postmortem tissue analyses, and experimental models supports the involvement of ferroptosis in PD. Multimodal iron‐sensitive MRI studies indicate that iron deposition in the substantia nigra pars compacta is not merely a late disease byproduct, but can emerge early and progress with disease development. In cohorts including de novo and advanced PD patients as well as healthy controls, R2* values in the substantia nigra pars compacta are significantly elevated in PD. In the clinically more affected hemisphere, R2* values are negatively correlated with dopaminergic synthesis capacity measured by 18F‐DOPA positron emission tomography (PET) (Spearman *ρ* ≈ −0.30, *p* < 0.05), indicating a spatial and functional association between local iron accumulation and nigrostriatal dysfunction in vivo [236].

Quantitative susceptibility mapping (QSM) analyses further show that individuals with idiopathic REM sleep behavior disorder, a prodromal PD phenotype, have significantly higher magnetic susceptibility in the substantia nigra pars compacta than controls. QSM‐based multiregional signatures from the substantia nigra, external globus pallidus, and ventral tegmental area discriminate prodromal and manifest PD from controls with over 80% accuracy, suggesting that midbrain iron dyshomeostasis can be detected before motor onset [237]. Postmortem biochemical studies also confirm markedly increased nonheme iron levels in the substantia nigra of PD patients, accompanied by accumulation of lipid peroxidation products, including malondialdehyde, 4‐hydroxynonenal, and PTGS2, together with significantly reduced GPX4 expression. This molecular profile is consistent with ferroptosis‐related pathology [238, 239, 240].

Experimental models using MPP^+^, 6‐hydroxydopamine, SH‐SY5Y cells, primary midbrain neurons, and zebrafish show ferroptosis like changes, including iron accumulation, increased lipid peroxidation, altered mitochondrial morphology, and GPX4 downregulation. Ferroptosis inhibitors, including Ferrostatin‐1, Liproxstatin‐1, deferoxamine, and Trolox, markedly reduce cell death, supporting the functional contribution of ferroptotic mechanisms [241].

Beyond neuronal antioxidant imbalance, disturbed iron handling also reshapes the inflammatory environment of dopaminergic regions. Evidence from PD models indicates that aberrant iron accumulation and lipid peroxidation are closely associated with microglial activation and proinflammatory signaling. Iron‐dependent lipid peroxidation increases neuronal susceptibility to injury and can promote microglial inflammatory polarization through mechanisms involving NF‐κB signaling. However, current evidence suggests that lipid peroxidation in microglia mainly modulates inflammatory signaling rather than directly inducing canonical ferroptotic death [242, 243].

Astrocytic iron buffering is also impaired in PD, further aggravating regional iron dysregulation. Single cell and spatial transcriptomic analyses show marked downregulation of the NRF2, SLC7A11, and GPX4 antioxidant axis in astrocytes from the substantia nigra, indicating reduced capacity to neutralize free iron and lipid peroxides. This impairment may increase dopaminergic neuronal exposure to iron‐dependent oxidative stress [239]. These findings are consistent with previous evidence linking astrocytic dysfunction to cerebral iron accumulation and susceptibility to lipid peroxidation [242].

Clinical intervention studies further support the pathological relevance of iron dyshomeostasis in PD. Deferiprone, an orally active iron chelator capable of crossing the blood–brain barrier (BBB), reduces nigral iron levels, attenuates oxidative stress, and partially improves motor phenotypes in MPTP animal models. In a randomized, double blind, placebo controlled Phase II trial in early PD, deferiprone decreased basal ganglia iron accumulation [244]. Subsequent studies confirmed that deferiprone reduces nigrostriatal iron deposition, although consistent improvement in movement disorder society‐unified PD rating scale (MDS‐UPDRS) motor scores has not been observed. These findings suggest that iron‐dependent lipid peroxidation contributes to PD pathology, but iron removal alone may be insufficient to produce sustained clinical benefit [245].

In summary, the substantia nigra microenvironment in PD is characterized by elevated iron burden, increased lipid peroxidation, and impaired detoxification of lipid peroxides. These conditions create a redox state that favors ferroptosis and contributes to the selective vulnerability of dopaminergic neurons.

### 4.3 Amyotrophic Lateral Sclerosis

ALS is a fatal neurodegenerative disorder characterized by progressive degeneration of upper and lower motor neurons [246]. In ALS, RNA binding proteins such as TDP‐43 and fused in sarcoma (FUS) undergo abnormal nucleocytoplasmic redistribution and form insoluble aggregates, disrupting RNA splicing, transport, and translation while imposing sustained stress on proteostasis mechanisms [246, 247]. This proteostatic burden activates endoplasmic reticulum stress and the unfolded protein response. As disease stress persists, PERK and eIF2α pathway and IRE1 pathway may shift from adaptive signaling toward proapoptotic responses, accompanied by calcium imbalance and mitochondrial dysfunction [248, 249]. Astrocytes also shift from a supportive phenotype toward a neurotoxic state, with downregulation of the glutamate transporter excitatory amino acid transporter 2 (EAAT2) and altered metabolic and calcium signaling. These changes impair synaptic glutamate clearance and amplify excitotoxic injury [250]. Together, neuron intrinsic proteostatic stress and glial dysfunction contribute to the non‐cell autonomous motor neuron degeneration that characterizes ALS [246].

#### 4.3.1 RNA Metabolism and ER Stress–Related Death Pathways

Mutations in TAR DNA‐binding protein (*TARDBP*), *FUS*, and Chromosome 9 open reading frame 72 (*C9orf72*) are major genetic contributors to ALS, and many of their pathogenic effects converge on RNA metabolic disruption. These mutations alter the nucleocytoplasmic distribution and dynamic assembly of RNA binding proteins, leading to persistent defects in RNA splicing, transcriptional regulation, and RNA stability [251].

TDP‐43 and FUS are normally localized in the nucleus, where they participate in pre mRNA splicing, transcriptional regulation, and maintenance of RNA homeostasis. During acute stress, these proteins can undergo reversible nucleocytoplasmic translocation and transiently associate with stress granules, supporting translational repression and adaptive cellular responses [251, 252].

ALS‐associated mutations cause prolonged mislocalization of TDP‐43 and FUS, alter their liquid liquid phase separation behavior, and shift stress granules from low viscosity, reversible liquid assemblies toward more viscous or gel like states that resist disassembly after stress resolution [251]. Studies using human iPSC‐derived motor neurons show that *TARDBP* gene mutations significantly reduce TDP‐43 mobility within stress granules, as reflected by delayed fluorescence recovery after photobleaching. This finding indicates altered phase separation dynamics and reduced internal exchange within these condensates [253]. Mutant TDP‐43 also persists within stress granules after stress withdrawal and is accompanied by increased insoluble TDP‐43 species, suggesting that reversible ribonucleoprotein assemblies can evolve into stable pathological condensates [253].

A similar mechanism has been described for FUS. ALS linked mutations such as P525L rapidly promote transition from liquid assemblies to solid like states, aberrantly trap core stress granule components including TIA1 and hnRNPA1, and trigger Caspase‐3‐dependent apoptotic signaling. These findings connect dysregulated phase separation with neuronal death signaling in ALS [254].

In *C9orf72*‐related ALS, disruption of RNA metabolism is an early pathogenic event. Expanded G4C2 repeat sequences form sense and antisense RNA foci in the nucleus, aberrantly sequestering RNA binding proteins and nuclear speckle‐associated factors. This sequestration causes widespread post transcriptional splicing defects and impairs nucleocytoplasmic transport [255]. Increasing evidence indicates that RNA foci are linked to nuclear speckle dysfunction, together producing global splicing abnormalities and RNA metabolic stress in C9‐ALS/FTD pathology [256].

Beyond RNA‐mediated toxicity, repeat‐associated non‐AUG translation generates dipeptide repeat proteins (DPRs), most notably poly‐GA, poly‐GR, and poly‐PR, which readily aggregate in C9‐ALS. These DPRs disrupt the organization of membraneless organelles through abnormal phase separation. Arginine‐rich poly‐GR and poly‐PR interfere with nucleocytoplasmic transport machinery by perturbing the Ran/RanGAP axis, promoting nuclear clearance and cytoplasmic mislocalization of TDP‐43, and accelerating its conversion into insoluble, toxic species [257]. Under stress, poly‐PR also induces the formation of low‐fluidity TDP‐43 nuclear condensates, a process dependent on the long noncoding RNA nuclear paraspeckle assembly transcript 1 and modulated by heat shock protein 70 activity, highlighting a mechanism by which DPRs drive pathological phase transitions of TDP‐43 [255].

Studies further show that poly‐GA aggregation colocalizes with TDP‐43 pathology and exacerbates proteostasis impairment. Poly‐GA aggregates impede proteasomal activity, disrupt protein quality control, and promote aberrant phosphorylation and accumulation of endogenous TDP‐43, leading to progressive motor dysfunction and neuronal loss in vivo [258, 259]. In parallel, DPR mediated abnormalities in stress granule dynamics link RNA and protein toxicity. Poly‐PR and poly‐GR facilitate stress granule assembly and delay their resolution, resulting in persistent translational repression and amplification of cellular stress responses [260].

As dysregulated RNA metabolism and misfolded proteins accumulate, ER homeostasis becomes increasingly disrupted. Persistent activation of the PERK, IRE1α, and activating ATF6 branches of the unfolded protein response has been observed across multiple ALS models [261, 262]. In early stages, the unfolded protein response can be adaptive by attenuating translation and promoting ER‐associated degradation to reduce protein burden. With persistent ER stress, the unfolded protein response may lose its adaptive function and favor proapoptotic signaling. Sustained activation of the PERK–eIF2α–ATF4–C/EBP homologous protein (CHOP) axis increases CHOP expression, suppresses Bcl‐2 family proteins, and strengthens mitochondrial apoptotic signaling, thereby promoting motor neuron death [263]. In the SOD1^G93A^ mouse model, inhibition of eIF2α dephosphorylation alleviates ER stress, improves motor neuron survival, and delays disease progression [263]. Similarly, in TDP‐43^A315T^ overexpression models, persistent PERK/eIF2α phosphorylation and upregulation of ATF4 and CHOP correlate with mitochondrial impairment and motor dysfunction, suggesting that aberrant activation of the unfolded protein response alone can precipitate neuronal death [252, 264].

Disruption of proteostasis also manifests as dysfunction of the ubiquitin–proteasome system. C terminal fragments of TDP‐43 are usually degraded by the proteasome. Proteasomal inhibition, however, rapidly induces their cytoplasmic translocation and formation of phosphorylated, ubiquitinated insoluble inclusions, significantly increasing neuronal apoptosis [265, 266]. In primary neurons, treatment with the proteasome inhibitor MG‐132 for 4 h induces nuclear export of TDP‐43, stable inclusion formation, Caspase‐3 activation, and increased TUNEL positivity [267]. In addition, poly‐GA directly inhibits both 20S and 26S proteasome activity, resulting in accumulation of proteasome substrates, reduced mitochondrial membrane potential, and increased ROS production [268]. Combined impairment of proteasomal degradation and autophagy lysosome clearance promotes misfolded protein accumulation, reinforcing ER stress, mitochondrial dysfunction, and oxidative damage. This progression can push motor neurons beyond reversible stress adaptation toward irreversible RCD.

#### 4.3.2 Astrocytic Toxicity and Glutamate‐Mediated Excitotoxicity

In ALS, astrocytic dysfunction is a major contributor to glutamate‐mediated excitotoxicity. Healthy astrocytes maintain synaptic glutamate homeostasis mainly through high affinity transporters such as glutamate transporter 1 (GLT‐1)/EAAT2, which clear extracellular glutamate and prevent excessive activation of N‐methyl‐D‐aspartate and α‐amino‐3‐hydroxy‐5‐methyl‐4‐isoxazolepropionic acid receptors [269]. In the motor cortex and spinal cord of individuals with ALS, however, GLT‐1 protein levels are reduced by approximately 71 ± 12%, with some cases showing declines greater than 90%. This reduction closely correlates with impaired glutamate uptake capacity [250].

EAAT2/GLT‐1 expression is significantly reduced in ALS spinal cord and motor cortex tissue and is accompanied by abnormal EAAT2 mRNA splicing and transcriptional dysregulation. These changes further impair glutamate clearance and aggravate excitotoxic injury, thereby promoting motor neuron degeneration [270, 271]. In *C9orf72* mouse models, pharmacological or genetic enhancement of EAAT2 expression reduces extracellular glutamate levels and mitigates excitotoxic motor neuron injury. These findings support EAAT2 restoration as a potential strategy for reducing excitotoxic stress in ALS [272].

Excitotoxicity not only induces neuronal death through Ca^2^^+^ influx and mitochondrial dysfunction, but also worsens mislocalization of TDP‐43 and FUS. In cortical and spinal motor neurons, pathological glutamate exposure induces Ca^2^^+^‐dependent translocation of FUS from the nucleus to the cytoplasm and is associated with stress granule formation [273]. Similarly, glutamate‐mediated excitotoxicity promotes cytoplasmic mislocalization and aggregation of TDP‐43, linking excitatory stress to RNA binding protein pathology. Small molecule modulators such as Sephin1 reduce cytoplasmic accumulation and aggregation of TDP‐43, decrease mitochondrial ROS generation, alleviate ER stress, and improve motor neuron survival in ALS models [274].

Thus, astrocyte‐associated glutamate excitotoxicity contributes to motor neuron death through both Ca^2^^+^ overload with mitochondrial dysfunction and pathological redistribution of RNA binding proteins. This mechanism helps explain how astrocytic dysfunction amplifies non‐cell autonomous motor neuron degeneration in ALS.

### 4.4 Huntington's Disease

HD is an autosomal dominant neurodegenerative disorder caused by abnormal expansion of cytosine–adenine–guanine trinucleotide repeat trinucleotide repeats in exon 1 of the *HTT* gene on Chromosome 4 [275, 276]. This mutation produces an expanded polyglutamine tract in the N terminus of huntingtin and generates conformationally unstable mHTT. Persistent mHTT expression disrupts neuronal homeostasis, with mitochondrial dysfunction emerging as an early and sustained pathological event [277].

mHTT interacts abnormally with regulators of mitochondrial fission and fusion, promoting excessive fission and limiting fusion. This imbalance fragments mitochondrial networks and is accompanied by reduced membrane potential and impaired ATP production [277, 278]. As mitochondrial injury progresses, activities of respiratory chain Complexes II and III decline, ROS accumulates, and mitochondrial Ca^2^^+^ buffering becomes impaired, leading to disrupted energy metabolism and redox imbalance. PINK1/Parkin‐mediated mitochondrial quality control and mitophagy are also reduced, allowing dysfunctional mitochondria to accumulate and amplify oxidative stress and death signaling [278]. Such mitochondrial stress is particularly harmful to striatal medium spiny neurons, which are highly vulnerable to glutamate‐mediated Ca^2^^+^ influx and metabolic stress [278].

mHTT expression in astrocytes also impairs the expression and function of the GLT‐1 /EAAT2, reducing extracellular glutamate clearance and increasing excitatory stress in the local microenvironment [279]. Simultaneously, microglial activation and reactive astrocyte transcriptional remodeling sustain proinflammatory signaling and complement cascade activation. These glial changes expose neurons to reduced metabolic support, increased excitotoxic pressure, and persistent inflammatory stress [280, 281]. mHTT driven mitochondrial dysfunction, impaired mitophagy, excitotoxic stress, and glial inflammatory remodeling together promote progressive striatal neuronal dysfunction and increase vulnerability to RCD [275].

#### 4.4.1 Autophagy–Lysosomal Dysfunction Induced by mHTT

mHTT contains an expanded polyglutamine tract, shows reduced conformational stability, and readily forms soluble oligomers and insoluble inclusions. Persistent intracellular accumulation of mHTT places sustained pressure on the autophagy lysosomal system. In HD models, autophagic dysfunction is characterized mainly by impaired flux rather than global suppression of autophagy initiation, indicating inefficient degradation after autophagosome formation. In neurons derived from patients with HD, BECN1 levels are decreased, whereas LC3B‐II levels are elevated and the LC3B‐II/I ratio is reduced, suggesting imbalance between autophagosome formation and maturation. In addition, LC3B, p62, and LAMP1‐positive structures accumulate as abnormal clusters within dendrites and axons, indicating retention of autophagic components in neurites without effective clearance [282].

Further analysis shows that retrograde transport of autophagosomes from neurites to the soma is markedly impaired, limiting their access to lysosomes. Accordingly, rapamycin treatment increases the number of LC3‐positive structures but does not improve degradation efficiency. Instead, it exacerbates autophagosome accumulation. These findings indicate that the primary defect in HD neurons lies in autophagosome maturation, trafficking, and terminal degradation, rather than in autophagy induction [282].

A similar “empty autophagosome” phenotype has been observed in striatal tissue from HD mice and fibroblasts derived from patients with HD. In these models, autophagosome numbers are markedly increased, but their lumens contain few recognizable substrates, while lysosomal fusion appears preserved. This pattern suggests defective substrate recognition and cargo loading, which prevents macroautophagy from effectively reducing intracellular proteotoxic burden [283]. Wild‐type huntingtin acts as a scaffold for selective autophagy by binding LC3, p62, and ULK1, thereby facilitating cargo delivery to autophagosomes. Expression of mHTT or loss of functional huntingtin disrupts this process, reduces p62 and LC3‐dependent cargo recruitment, and particularly impairs selective clearance of damaged mitochondria. This defect weakens mitophagy and mitochondrial quality control [284, 285].

mHTT further alters transcriptional programs required for autophagy and lysosomal homeostasis, leading to progressive impairment of autophagic flux. TFEB, a transcription factor that controls lysosomal biogenesis and autophagy‐related gene expression, contains a prion like domain that readily coaggregates with mHTT, resulting in sequestration of TFEB into insoluble fractions. This process is associated with reduced nuclear localization of TFEB and downregulation of CLEAR network genes, progressively impairing lysosome formation and replenishment [286, 287]. Neurons expressing mHTT also show increased lysosomal membrane damage, reflected by persistent Galectin‐3 recruitment to compromised membranes. Suppression of the TFEB/TFE3 axis limits activation of ESCRT‐mediated membrane repair, lysophagy, and de novo lysosome biogenesis, resulting in widespread impairment of lysosomal quality control [286].

HTT and Huntingtin‐associated protein 1 normally facilitate dynein‐dependent retrograde transport of autophagosomes along axons. In primary neurons and HD knock in mouse cells, however, polyQ expanded huntingtin or loss of wild‐type huntingtin markedly weakens this transport process. As a result, autophagosomes containing damaged mitochondria or aggregated proteins fail to efficiently reach and fuse with lysosomes [288].

Impaired substrate recognition, vesicular trafficking, lysosomal repair, and lysosome regeneration gradually reduce the renewal capacity of the autophagy lysosomal system. Accumulation of toxic protein aggregates and damaged organelles then promotes chronic inflammatory signaling and cell death pathway activation, contributing to progressive neurodegeneration in HD.

#### 4.4.2 Non‐Cell‐Autonomous Neurotoxicity Mediated by mHTT

Proteostatic imbalance caused by mHTT is not restricted to neurons. Astrocytes also express and accumulate mHTT, and nuclear inclusions are closely associated with downregulation of the GLT‐1 /EAAT2. In primary astrocyte cultures and HD mouse brain tissue, mHTT suppresses GLT‐1 transcription and reduces glutamate uptake, leading to impaired extracellular glutamate clearance and increased neuronal vulnerability to excitotoxic stimulation [279]. In astrocyte‐specific mHTT transgenic mice, even relatively low mHTT expression causes age‐dependent weight loss, motor dysfunction, and premature death, accompanied by reduced GLT‐1 expression and impaired glutamate uptake. This observation suggests that astrocytic metabolic and homeostatic dysfunction alone is sufficient to impair neural function, even in the absence of substantial neuronal mHTT accumulation [289]. Similarly, in models with viral vector‐mediated striatal mHTT expression, astrocyte activation and GLT‐1 downregulation progressively worsen over the course of disease and correlate closely with striatal dysfunction severity [290].

Beyond impaired metabolic support, inflammasome‐mediated pyroptotic signaling contributes to glial and vascular pathology in HD. In R6/2 mice, NLRP3, activated Caspase‐1, and N‐terminal GSDMD‐N progressively increase in the striatum, accompanied by elevated IL‐1β and IL‐18 secretion and neuronal nuclear condensation, consistent with pyroptotic signaling. Pharmacological inhibition of poly(ADP‐ribose) polymerase 1 (PARP‐1) attenuates NLRP3, Caspase‐1, and GSDMD activation, reduces neuroinflammation and neuronal loss, and improves motor phenotypes [291, 292].

Cerebrovascular endothelial cells are also affected by mHTT‐associated inflammasome activation. mHTT can activate the NLRP3 inflammasome in endothelial cells, triggering GSDMD‐dependent pyroptosis and BBB disruption, thereby indirectly compromising neuronal survival [115]. Endothelial pyroptosis induced by mHTT is associated with marked downregulation of tight junction proteins zonula occludens‐1, claudin‐5, and occludin, together with increased Evans blue extravasation and vascular permeability. shRNA‐mediated knockdown or pharmacological inhibition of NLRP3, as well as blockade of GSDMD cleavage, significantly attenuates BBB leakage, reduces striatal neuronal apoptosis, and partially restores motor performance. Endothelial pyroptosis therefore represents a vascular pathway through which mHTT‐associated proteostatic stress may exacerbate neuronal degeneration in HD [115].

mHTT therefore promotes non‐cell autonomous neurotoxicity through astrocytic metabolic dysfunction, inflammatory pyroptotic signaling, and endothelial barrier disruption. These processes expose striatal neurons to impaired glutamate clearance, inflammatory mediators, and BBB leakage, accelerating functional decline and RCD in HD.

#### 4.4.3 Oxidative Stress and Mitochondrial Fragmentation

mHTT‐induced disruption of mitochondrial dynamics and oxidative stress contributes to neuronal structural and functional impairment. mHTT abnormally interacts with the mitochondrial fission protein Drp1 and enhances its GTPase activity, increasing Drp1 recruitment to the mitochondrial outer membrane and destabilizing the balance between fission and fusion [293]. In STHdh^Q111/Q111^  cells and patient‐derived fibroblasts, mitochondrial Drp1 enrichment increases by approximately threefold to fourfold compared with controls. In R6/2 mouse striatal neurons, normally elongated mitochondria become highly fragmented, with mean mitochondrial length reduced by 40–50% and cristae density decreased by approximately 50%. These observations suggest that excessive mitochondrial fission is accompanied by marked intramitochondrial structural damage [293].

Mitochondrial fragmentation is accompanied by clear bioenergetic failure in HD neurons, including a marked reduction in mitochondrial membrane potential, a 30–40% decrease in ATP levels, and an approximately 1.5–2‐fold increase in mitochondrial ROS production, which promotes oxidative mtDNA damage [293, 294]. In STHdh^Q111/Q111^ cells, both maximal respiration and spare respiratory capacity are significantly diminished. Inhibition of the base excision repair enzyme apurinic/apyrimidinic endodeoxyribonuclease 1 aggravates mtDNA damage and further compromises electron transport chain activity, thereby worsening ATP deficiency [295]. mtDNA oxidation may therefore actively contribute to mitochondrial failure rather than merely reflect secondary oxidative injury. Elevated 8‐hydroxy‐2′‐deoxyguanosine levels have also been detected in the striatum and peripheral blood of patients with HD, and these increases correlate positively with ATP depletion and apoptotic neuronal morphology [296, 297].

Energy metabolic failure further drives synaptic degeneration. During mitochondrial fragmentation and ATP reduction, anterograde mitochondrial transport along axons slows, compromising energy supply at presynaptic terminals. In the R6/2 mouse striatum, synaptic proteins such as synaptophysin are significantly reduced in parallel with decreased neuronal viability, supporting the role of mitochondrial dynamic disruption and oxidative injury in early synaptic degeneration [294].

Genetic or pharmacological inhibition of Drp1 hyperactivation can partially reverse these pathological changes. In R6/2 mice, expression of dominant negative Drp1K38A or administration of the Drp1 inhibitory peptide P110‐TAT reduces mitochondrial fragmentation, improves cristae structure, enhances ATP production and respiratory chain efficiency, and increases rotarod performance by 30–40%. These interventions also reduce striatal neuronal loss and extend lifespan, supporting a pathogenic role for dysregulated mitochondrial dynamics in HD progression [293].

Further evidence comes from astrocyte studies. In R6/1 mice and primary striatal astrocyte cultures, mitochondrial density increases while individual organelle volume decreases. These changes are accompanied by elevated basal respiration, ATP coupled respiration, maximal respiratory capacity, mitochondrial superoxide production, and lactate release. This profile suggests that HD astrocytes adopt a hypermetabolic state with increased oxidative stress susceptibility [298]. Coculture experiments show that HD astrocytes transfer mitochondria with intact membrane potential to adjacent neurons. However, these transferred mitochondria increase neuronal ROS levels and reduce dendritic branching complexity and spine density. Removing mitochondrial particles from conditioned medium abolishes these effects, indicating that astrocytic mitochondrial transfer can directly disturb neuronal redox homeostasis [298]. This observation is consistent with multiomics analyses showing marked metabolic reprogramming in astrocytes from HD vulnerable brain regions, with the extent of reprogramming closely associated with regional neurodegeneration [299].

Oxidative stress also promotes lipid peroxidation by stabilizing 5‐lipoxygenase (ALOX5) and its activating partner 5‐lipoxygenase‐activating protein, increasing lipid ROS accumulation, downregulating GPX4, and inducing a ferroptosis like phenotype. Genetic deletion of Alox5 or pharmacological inhibition with Ferrostatin‐1 improves motor performance, reduces striatal atrophy, and prolongs survival in R6/2 mice, indicating that ALOX5‐mediated ferroptosis contributes to HD progression [300].

Despite sustained mitochondrial damage during HD progression, neurons retain compensatory antioxidant responses. Under mitochondrial stress, HdhQ111 cells and HD patient iPSC‐derived neurons show robust upregulation of coiled‐coil‐helix‐coiled‐coil‐helix domain containing 2 (CHCHD2), a mitochondrial protein that activates the Nrf2–antioxidant response element pathway and induces antioxidant genes such as HO‐1 and NQO1. CHCHD2 overexpression partially restores mitochondrial membrane potential and suppresses Caspase‐3 activation, suggesting a protective response against oxidative injury [301]. Treatment with the mitochondria‐targeted antioxidant XJB‐5‐131 significantly reduces mtDNA oxidative damage and lipid peroxidation in the striatum and cortex of R6/2 mice, improves mitochondrial respiration, and extends lifespan, supporting mitochondrial redox balance as a therapeutic target in HD [302].

By disrupting mitochondrial dynamics and redox balance, mHTT creates persistent bioenergetic instability in neurons and glia. Together with ferroptotic and pyroptotic signaling, this disturbance contributes to progressive striatal neurodegeneration in HD.

In conclusion, dysregulated RCD involves overlapping disturbances in proteostasis, mitochondrial integrity, excitotoxicity, lipid redox balance, and glial function. Although AD, PD, ALS, and HD differ in their initiating pathogenic proteins and vulnerable neuronal populations, these processes converge on interconnected death and inflammatory signaling pathways.

## 5 Therapeutic Strategies Targeting Cell Death Pathways and Clinical Translational Perspectives

In recent years, research aimed at targeting RCD pathways has advanced considerably, with growing evidence that pharmacological inhibitors or gene‐based interventions directed at key molecular regulators of cell death can alleviate neuronal injury and improve functional outcomes in multiple ND models, provided that sufficient CNS exposure or effective delivery systems are ensured [303, 304]. These advances have established a crucial experimental foundation for translational research (Table 2).

**TABLE 2:** Representative therapeutic strategies targeting RCD pathways in NDs.

RCD pathway	Disease context	Target/pathway	Intervention or experimental tool	Evidence stage	Model or subjects	Key mechanistic or translational interpretation	Representative references
Apoptosis and apoptosis‐associated degeneration	ALS	Astrocyte‐derived TRAIL–DR5 signaling activates Caspase‐8‐dependent extrinsic apoptosis in motor neurons	DR5‐neutralizing antibody	Preclinical	SOD1^G93A^ mice; NSC34‐G93A motor neuron models	Reactive astrocytes promote non‐cell‐autonomous motor neuron apoptosis through TRAIL–DR5 signaling, supporting DR5 blockade as a potential strategy to reduce selective motor neuron vulnerability.	[305]
Apoptosis and oxidative stress‐associated degeneration	AD, toxin‐induced experimental model	Aluminum‐induced oxidative and nitrosative stress, glutathione depletion, lipid peroxidation, Caspase‐8 activation, and tau hyperphosphorylation	Benfotiamine; donepezil comparator	Preclinical	Aluminum chloride‐induced AD rat model	Benfotiamine attenuates oxidative stress and Caspase‐8‐associated apoptotic signaling in an AD‐like neurotoxicity model. This model should be described as toxin‐induced rather than genetic or sporadic AD.	[306]
Apoptosis‐associated systemic stress response	ALS	Redox imbalance, immune dysregulation, hypoxia‐related stress, and apoptosis‐associated vulnerability	Bu‐Shen‐Jian‐Pi formula	Early clinical	Randomized, multicenter, double‐blind controlled exploratory study in ALS patients, *n* = 127	The intervention may modulate redox and immune‐inflammatory stress responses associated with apoptosis‐related vulnerability, although it does not directly target a single executioner apoptosis pathway.	[307]
Apoptosis‐to‐necrotic shift and secondary phagoptosis	PD	Loss of Caspase‐3‐dependent apoptotic execution shifts dopaminergic neurons toward necrotic‐like degeneration; Galectin‐3 promotes microglial engulfment	Dopaminergic neuron‐specific Caspase‐3 deletion	Preclinical mechanistic evidence	MPTP‐induced PD mouse model; primary midbrain neuron–BV2 microglia coculture	Suppression of apoptotic execution can redirect neuronal injury toward necrotic degeneration and microglia‐mediated phagoptosis, illustrating cell‐death plasticity in dopaminergic injury.	[308]
Pyroptosis and inflammasome‐associated inflammatory cell death	HD	Inflammasome activation and pyroptotic cell death in mutant huntingtin‐associated neurodegeneration	Pathway‐level inflammasome analysis	Preclinical	R6/2 mouse model of HD	R6/2 mice show inflammasome activation and pyroptosis‐associated molecular changes, supporting pyroptotic inflammatory cell death as a disease‐relevant mechanism in HD.	[291]
Pyroptosis and inflammasome‐associated inflammatory cell death	HD	PARP‐1‐linked modulation of inflammasome activation and pyroptosis‐related responses	Olaparib, PARP‐1 inhibitor	Preclinical	R6/2 mouse model of HD	PARP‐1 inhibition modulates inflammasome and pyroptosis‐related signaling in HD models, providing pharmacological evidence that pyroptotic inflammatory responses may be therapeutically adjustable.	[292]
Pyroptosis and inflammasome‐associated inflammatory cell death	HD	Mutant huntingtin activates endothelial NLRP3 inflammasome signaling, Caspase‐1 activation, IL‐1β maturation, tight‐junction disruption, and BBB damage	NLRP3 siRNA knockdown	Preclinical	HD‐related endothelial cell models; brain tissue analyses	Endothelial NLRP3‐mediated pyroptosis promotes BBB disruption and secondary neuronal injury, supporting vascular pyroptosis as a contributor to HD pathology.	[115]
Pyroptosis and inflammasome‐associated inflammatory cell death	PD	NLRP3 inflammasome activation, Caspase‐1/11 signaling, GSDMD cleavage, and IL‐1β/IL‐18 release	Gsdmd knockout; disulfiram	Preclinical	MPTP and α‐synuclein A53T PD mouse models; LPS‐stimulated primary microglia; MPP^+−^treated neurons	GSDMD‐mediated pore formation sustains microglial inflammatory activation and contributes to dopaminergic neuronal degeneration in PD models.	[60]
Pyroptosis and inflammasome‐associated inflammatory cell death	PD	Parkin deficiency releases inhibitory control over NLRP3 inflammasome activation, leading to Caspase‐1 activation and GSDMD cleavage	MCC950; AAV‐mediated Parkin overexpression	Preclinical	Rotenone‐induced PD mouse model; SN4741 dopaminergic neuronal cultures	Impaired mitochondrial quality control promotes pyroptotic signaling, whereas restoration of Parkin activity constrains NLRP3 activation and reduces neuronal injury.	[309]
Pyroptosis marker activation	ALS	Microglial GSDMD activation downstream of inflammasome signaling	Genetic deletion of Gsdmd	Preclinical	SOD1^G93A^ mice with or without Gsdmd deletion	GSDMD activation is detectable in microglia, but GSDMD loss does not rescue disease progression, indicating that pyroptosis markers may not always represent an executionally required pathway.	[61]
Pyroptosis pathway druggability	Neurodegeneration‐relevant translational evidence	NLRP3 inflammasome activation and IL‐1β release	DFV890, oral NLRP3 inhibitor	Translational enabling evidence, Phase I	Healthy adult volunteers	NLRP3 inhibition suppresses inflammasome‐dependent inflammatory output in humans. Because this is not a neurodegenerative disease cohort, it should be interpreted as target‐engagement evidence rather than disease‐specific efficacy evidence.	[310]
Ferroptosis and iron‐dependent lipid peroxidation	AD	GPX4 functional enhancement and AMPK/Nrf2/GPX4 antioxidant signaling activation	Thonningianin A	Preclinical	AD cellular models; C. elegans AD models	Thonningianin A suppresses neuronal ferroptosis by activating GPX4 and reinforcing antioxidant signaling, thereby reducing iron accumulation, lipid peroxidation, and ferroptotic stress in AD‐related models.	[311]
Ferroptosis and iron‐dependent lipid peroxidation	AD	Aβ‐induced iron overload, lipid peroxidation, glutathione depletion, GPX4 and SLC7A11 dysregulation	Tetrahedral framework nucleic acids	Preclinical	Aβ‐treated N2a cells; APP/PS1 mice	tFNAs reduce Aβ‐induced ferroptotic stress by restoring glutathione homeostasis, reducing iron and lipid peroxidation, and normalizing ferroptosis‐related gene expression.	[312]
Ferroptosis and iron‐dependent lipid peroxidation	AD	Gut microbiota‐derived lysophosphatidylcholine, neuronal lipid metabolism, GPR119‐associated stress signaling, and ACSL4‐linked lipid peroxidation	Microbiota‐derived LPC; Liproxstatin‐1 comparator	Preclinical	5×FAD and 3×Tg‐AD mice; ferroptosis‐induced neuronal cultures	Microbiota‐derived LPC links gut microbial lipid metabolism to neuronal ferroptotic vulnerability, amyloid pathology, and inflammatory stress in AD models.	[313]
Ferroptosis and iron‐dependent lipid peroxidation	PD	α‐Synuclein‐associated iron dyshomeostasis, reduced ferritin and ferroportin, lipid peroxidation, and GPX4 vulnerability	Ferrostatin‐1; deferoxamine comparator	Preclinical and human tissue‐supported mechanistic evidence	Postmortem substantia nigra from PD patients; LUHMES dopaminergic neurons; primary mouse neurons; α‐synuclein‐expressing C. elegans	α‐Synuclein accumulation disrupts neuronal iron handling and increases susceptibility to lipid peroxidation‐driven ferroptotic injury.	[314]
Ferroptosis and iron‐dependent lipid peroxidation	PD	PI3K–AKT‐mediated suppression of NCOA4‐dependent ferritinophagy, stabilization of ferritin stores, and reduced labile iron release	Formononetin; Parabacteroides merdae supplementation; pathway perturbation	Preclinical	MPTP‐induced PD mouse model	Microbiota‐derived formononetin regulates iron mobilization through PI3K–AKT and ferritinophagy control, reducing dopaminergic ferroptotic susceptibility.	[315]
Ferroptosis and iron‐dependent lipid peroxidation	PD	TRPV4‐mediated Ca^2+^ influx, DMT1‐dependent iron uptake, reduced ferritin buffering, and GPX4 suppression	GSK2193874; TRPV4 siRNA; AAV‐mediated TRPV4 knockdown or overexpression	Preclinical	MPP^+−^treated PC12 cells; MPTP‐induced PD mouse model	TRPV4 couples Ca^2+^ dysregulation to iron overload and lipid peroxidation, identifying ion‐channel‐mediated ferroptosis as a PD‐relevant mechanism.	[316]
Ferroptosis and iron‐dependent lipid peroxidation	HD	Mitochondrial Complex II inhibition, ROS accumulation, NOX2 activation, iron‐dependent lipid peroxidation, and caspase‐independent cell death	Ferrostatin‐1; deferoxamine	Preclinical	STHdh^Q111^ and STHdh^Q7^ striatal cell lines	Mutant huntingtin increases ferroptotic vulnerability through mitochondrial dysfunction, ROS accumulation, and iron‐dependent lipid peroxidation.	[317]
Ferroptosis pathway druggability	Neurodegeneration‐relevant translational evidence	Lipid radical scavenging and blood–brain barrier permeability	Ferfluor‐1	Preclinical enabling evidence	Canonical ferroptosis models, including HT‐1080 and HEK293T cells exposed to erastin or RSL3	Ferfluor‐1 supports the feasibility of developing chemically stable and brain‐accessible ferroptosis inhibitors. Because the models are not neurodegenerative disease models, this row should be interpreted as enabling evidence.	[318]
Necroptosis and necroinflammation	AD	RIPK1, RIPK3, and MLKL activation in granulovacuolar degeneration structures; tau‐associated necrosome activation	Ponatinib; dabrafenib; Nec‐1s; tau‐seed stimulation	Preclinical	APP23×TAU58 mice; TAU22 and TAU58 tau models; primary neurons exposed to human AD‐derived tau seeds	Aggregated phosphorylated tau is sufficient to activate necroptosis‐associated signaling within GVD compartments, whereas Aβ alone does not robustly engage this pathway.	[319]
Necroptosis and necroinflammation	AD	Aβ‐induced mitochondrial oxidative stress, mtDNA fragmentation, Z‐conformation nucleic‐acid sensing by ZBP1, and RIPK1 kinase activation	Zbp1 deletion; Ripk1^D138N^ kinase‐inactive mutation; Nec‐1s; ponatinib	Preclinical	5×FAD mice; Zbp1^−/−^ mice; Ripk1^D138N/D138N^ mice; primary microglia	Aβ‐associated mitochondrial injury generates structurally altered mtDNA species that activate ZBP1–RIPK1‐dependent microglial inflammatory programs rather than immediate necrotic cell death.	[320]
Necroptosis‐associated glial injury	ALS	RIPK1 kinase activation, RIPK3 and MLKL signaling in astrocytes and microglia	Genetic and pharmacological inhibition of RIPK1 kinase activity	Preclinical and human tissue‐supported evidence	Postmortem ALS spinal cord tissue; SOD1^G93A^ mice; human iPSC‐derived motor neuron–astrocyte–microglia triculture	RIPK1 activation in glial cells amplifies necroinflammatory programs and contributes to non‐cell‐autonomous motor neuron toxicity.	[321]
Necroptosis and necroinflammation	PD	MLKL as terminal executioner downstream of RIPK3	Mlkl genetic deletion	Preclinical	α‐Synuclein A53T mice crossed with Mlkl^−/−^ mice	MLKL deletion attenuates neuroinflammation and neuronal degeneration, supporting necroptotic execution as a contributor to α‐synuclein‐associated pathology.	[322]
RIPK1‐dependent necroinflammation	Neurodegenerative disease translational studies, AD and ALS	RIPK1 kinase function and downstream RIPK3/MLKL‐associated inflammatory signaling	SAR443060, also known as DNL747	Early clinical, Phase I/Ib	Healthy volunteers; AD and ALS patients	CNS‐penetrant RIPK1 inhibition suppresses phosphorylated RIPK1 signaling and demonstrates target engagement in humans, providing clinical translation support for RIPK1‐directed strategies.	[323]
RIPK1‐dependent necroinflammation	Inflammatory and degenerative disease translational evidence	Selective RIPK1 kinase inhibition with preserved scaffolding function	SIR9900	Early clinical, Phase I	Healthy adult and elderly volunteers	Systemic RIPK1 inhibition achieves sustained kinase suppression with measurable CNS exposure and peripheral target engagement.	[324]
RIPK1 pathway druggability	Neurodegeneration‐relevant translational enabling evidence	RIPK1 kinase activity assessed by pSer166‐RIPK1 suppression	SAR443820, also known as DNL788	Early clinical, Phase I	Healthy adult participants	RIPK1 kinase inhibition produces robust pathway suppression in humans, supporting target druggability for neuroinflammation‐associated neurodegenerative disease.	[303]
RIPK1 pathway druggability	Neurodegeneration‐relevant translational enabling evidence	RIPK1‐dependent inflammatory signaling and cytokine output	GDC‐8264	Early clinical, Phase I randomized placebo‐controlled	Healthy adult volunteers	RIPK1 inhibition produces dose‐dependent suppression of inflammatory outputs in whole‐blood assays, but this remains target‐engagement rather than disease‐efficacy evidence.	[325]
RIPK1 pathway druggability	Translational target‐engagement evidence	RIPK1 kinase‐driven cytokine output	GFH312	Early clinical, Phase I randomized placebo‐controlled	Healthy adult volunteers	GFH312 shows predictable pharmacodynamic suppression of RIPK1‐mediated inflammatory readouts in humans.	[326]
Necroptosis execution	Mechanistic enabling evidence relevant to neurodegeneration	MLKL activation and oligomerization downstream of RIPK3	Apomorphine; oxidized apomorphine	Preclinical enabling evidence	In vitro necroptosis assays; murine DSS colitis and acetaminophen liver injury models	Apomorphine interferes with MLKL activation and oligomerization. Because the in vivo models are not neurodegenerative, this row should be interpreted as MLKL druggability evidence rather than disease‐specific therapeutic evidence.	[327]
Autophagy, mitophagy, and autophagy–lysosome dysfunction	AD	PINK1/Parkin‐dependent mitophagy, mitochondrial–lysosomal quality control, and cathepsin Z‐mediated degradative capacity	Urolithin A	Preclinical	APP/PS1, 3×Tg‐AD, and 3×Tg‐AD/Polβ^+/−^ mice	Restoring mitophagy flux and lysosomal proteolysis improves mitochondrial and proteostatic homeostasis and limits amyloid and tau pathology.	[328]
Autophagy, mitophagy, and autophagy–lysosome dysfunction	AD	ULK1, PINK1, and Parkin‐mediated mitochondrial clearance	Deoxytrillenoside CA and epitrillenoside CA	Preclinical	HT‐22 and PC12 AD‐related cellular models; C. elegans AD models; APP/PS1 mice	DTCA and ETCA promote mitophagic clearance, reduce Aβ and phosphorylated tau accumulation, and distinguish adaptive mitophagy from maladaptive autophagy‐associated cell death.	[329]
Autophagy and cellular stress adaptation	Early AD	Sigma‐1 receptor‐dependent endoplasmic reticulum–mitochondrial communication and proteostatic clearance	Blarcamesine, ANAVEX2‐73	Clinical, randomized double‐blind placebo‐controlled Phase IIb/III	Early AD patients, *n* = 508 enrolled, ITT population *n* = 462; 48‐week treatment	SIGMAR1 activation may modulate autophagy and cellular stress‐adaptation pathways upstream of overt neuronal loss. This row should be interpreted as clinical modulation of stress adaptation rather than direct proof of autophagy‐dependent neuroprotection.	[330]
Microglial autophagy and inflammatory modulation	PD	TGF‐β1–ALK‐dependent PI3K/Akt/mTOR signaling in microglia	Hypoxia‐preconditioned human olfactory mucosa‐derived MSCs; recombinant TGF‐β1; shRNA‐TGF‐β1; siRNA‐ALK	Preclinical and early clinical	α‐Syn‐induced SH‐SY5Y/BV2 coculture; MPTP‐treated mice; severe PD patients, *n* = 5	hOM‐MSC‐derived TGF‐β1 restores microglial autophagy homeostasis and reduces inflammatory activation, indirectly supporting neuronal survival.	[331]
Autophagy network modulation	ALS	RNA metabolism, iron handling, COX‐2‐dependent and independent inflammatory pathways, exosomal TDP‐43 and LC3 markers	PrimeC, fixed‐dose ciprofloxacin and celecoxib	Early clinical, Phase IIa open‐label	ALS patients with familial or sporadic disease, *n* = 15	PrimeC modifies autophagy‐ and RNA‐associated biomarkers, but the evidence does not directly demonstrate altered neuronal survival through an execution‐level RCD pathway.	[332]
mTOR‐dependent autophagy modulation	ALS	mTOR inhibition, autophagy activation, immune and metabolic signaling	Rapamycin, 1 or 2 mg/m^2^/day	Early clinical, randomized double‐blind placebo‐controlled exploratory trial	ALS patients, *n* = 63	Rapamycin is well tolerated and modulates immune or metabolic markers, but it does not clearly alter disease trajectory, indicating the limitations of broad autophagy induction as a stand‐alone therapeutic strategy.	[333]
Autophagy and innate immune homeostasis	ALS	TBK1 deficiency, impaired autophagy–lysosome flux, altered OPTN and p62 adaptor signaling, and inflammatory stress	Conditional cell‐type‐specific Tbk1 deletion	Preclinical	ALS‐relevant conditional Tbk1 knockout mice; neuron–glia interaction analyses; iPSC‐derived neural models	TBK1 loss disrupts selective autophagy and innate immune regulation, increasing motor neuron vulnerability through nonexecutional disease‐modifying mechanisms.	[334]
Autophagy–lysosome dysfunction	ALS	Defective clearance of misfolded proteins and altered inflammatory stress signaling	Genetic and molecular manipulation of autophagy–lysosome pathways	Preclinical	ALS‐associated cellular and molecular models	Autophagy–lysosome imbalance sensitizes motor neurons to proteotoxic and inflammatory stress, acting as an upstream vulnerability mechanism rather than a direct executioner cell‐death pathway.	[335]
mTOR‐independent autophagy enhancement	ALS	Autophagy and lysosomal activation independent of mTOR signaling	SLS‐005, intravenous trehalose	Early clinical, Phase II/III platform trial	HEALEY ALS Platform Trial, Regimen E	Trehalose was well tolerated but did not significantly modify ALS disease progression, highlighting the translational limitations of broad autophagy enhancement.	[336]

Abbreviations: AAV, adeno‐associated virus; ACSL4, acyl‐CoA synthetase long‐chain family member 4; AD, Alzheimer's disease; AKT, protein kinase B; ALK, activin receptor‐like kinase; ALS, amyotrophic lateral sclerosis; AMPK, AMP‐activated protein kinase; APP/PS1, amyloid precursor protein/presenilin 1; Aβ, amyloid β; BBB, blood–brain barrier; CNS, central nervous system; COX‐2, cyclooxygenase 2; DMT1, divalent metal transporter 1; DR5, death receptor 5; DTCA, deoxytrillenoside CA; ETCA, epitrillenoside CA; GPR119, G protein‐coupled receptor 119; GPX4, glutathione peroxidase 4; GSDMD, gasdermin D; GVD, granulovacuolar degeneration; HD, Huntington's disease; HEALEY, Healey ALS Platform Trial; IL‐18, interleukin‐18; IL‐1β, interleukin‐1β; iPSC, induced pluripotent stem cell; ITT, intention‐to‐treat; LC3, microtubule‐associated protein 1 light chain 3; LPC, lysophosphatidylcholine; LPS, lipopolysaccharide; MLKL, mixed lineage kinase domain‐like protein; MPP+, 1‐methyl‐4‐phenylpyridinium; MPTP, 1‐methyl‐4‐phenyl‐1,2,3,6‐tetrahydropyridine; MSCs, mesenchymal stem cells; mtDNA, mitochondrial DNA; mTOR, mechanistic target of rapamycin; NCOA4, nuclear receptor coactivator 4; NDs, neurodegenerative diseases; NLRP3, NOD‐like receptor family pyrin domain containing 3; NOX2, NADPH oxidase 2; NRF2, nuclear factor erythroid 2‐related factor 2; OPTN, optineurin; PARP‐1, poly(ADP‐ribose) polymerase 1; PD, Parkinson's disease; PI3K, phosphoinositide 3‐kinase; PINK1, PTEN‐induced kinase 1; RCD, regulated cell death; RIPK1, receptor‐interacting protein kinase 1; RIPK3, receptor‐interacting protein kinase 3; ROS, reactive oxygen species; shRNA, short hairpin RNA; SIGMAR1, sigma nonopioid intracellular receptor 1; siRNA, small interfering RNA; SLS‐005, intravenous trehalose; SOD1, superoxide dismutase 1; TBK1, TANK‐binding kinase 1; TDP‐43, TAR DNA‐binding protein 43; tFNAs, tetrahedral framework nucleic acids; TGF‐β1, transforming growth factor β1; TRAIL, tumor necrosis factor‐related apoptosis‐inducing ligand; TRPV4, transient receptor potential vanilloid 4; ULK1, unc‐51‐like autophagy activating kinase 1; ZBP1, Z‐DNA binding protein 1.

Within necroptosis research, the most compelling clinical translational evidence is derived from early trials of RIPK1 inhibitors capable of crossing the BBB. In a randomized, double‐blind, placebo‐controlled Phase I trial, the RIPK1 inhibitor SAR443060 demonstrated a favorable safety profile, with no dose‐limiting toxicities observed after either single or multiple daily administrations in healthy volunteers [323]. At steady state, SAR443060 was detectable in cerebrospinal fluid from both healthy participants and patients with AD or ALS. Peripheral blood mononuclear cells exhibited dose‐dependent suppression of phospho‐RIPK1 (Ser166) upon ex vivo TNF stimulation, confirming robust in vivo target engagement and sustained RIPK1 inhibition [323]. Building on this, an optimized analog, SAR443820 (DNL788), has been developed with improved CNS exposure and pharmacokinetic properties. After repeated daily oral administration, the cerebrospinal fluid to free plasma concentration ratio remained stable, and peripheral blood mononuclear cell analysis showed up to 90% inhibition of p‐S166–RIPK1 at trough levels. No treatment‐related serious adverse events were reported, supporting the feasibility of sustained central RIPK1 inhibition in humans [303]. However, these trials also show the gap between biochemical pathway inhibition and disease modification. SAR443060 achieved CNS exposure and measurable RIPK1 inhibition, but further development was limited by long‐term nonclinical toxicology concerns, indicating that compound‐specific safety liabilities can interrupt biologically plausible programs before efficacy is tested in ND populations [323]. Because RIPK1 participates in both inflammatory signaling and cell death responses, future trials will require stronger patient enrichment, brain relevant pharmacodynamic biomarkers, and evidence of pathway suppression in disease relevant cellular compartments, rather than reliance on peripheral blood markers alone [303].

Parallel studies have demonstrated that targeting ferroptosis can reduce neuroinflammation and improve motor phenotypes in ND models [304]. In mice with LPS‐induced neuroinflammation and cognitive impairment, Liproxstatin‐1 reduced hippocampal iron‐dependent lipid peroxidation and inflammatory responses, restored iron homeostasis, and improved behavioral performance [337]. In STHdh^Q7/Q111^ striatal cells, Ferrostatin‐1 and deferoxamine alleviated 3‐nitropropionic acid (3‐NP)‐induced cytotoxicity, increasing cell viability and reducing cell death [317]. In ALS models, GPX4 overexpression delayed disease progression, improved motor function, and prolonged survival [338]. In APP/PS1 mice, intranasal deferoxamine reduced cortical and hippocampal iron accumulation, decreased amyloid plaque and tau pathology, and improved cognitive performance [339].

However, clinical translation of ferroptosis‐directed therapy is more complex than simple suppression of iron accumulation or lipid peroxidation. n a multicenter randomized trial involving 372 patients with newly diagnosed PD, deferiprone reduced nigrostriatal iron content but was associated with worse clinical outcomes. The MDS‐UPDRS total score increased by 15.6 points in the deferiprone group and by 6.3 points in the placebo group, with a between group difference of 9.3 points, 95% confidence interval (CI) 6.3–12.2, *p* < 0.001. Dopaminergic therapy was initiated because of symptom progression in 22.0% of deferiprone treated participants compared with 2.7% of placebo treated participants [245]. This result suggests that nonselective iron chelation may disturb physiologically required iron pools or fail to reach the specific labile iron and lipid radical compartments that drive ferroptotic injury. Future ferroptosis‐directed strategies should therefore prioritize BBB permeable radical trapping agents, approaches that support GPX4 activity, and pharmacodynamic biomarkers capable of distinguishing pathological lipid peroxidation from physiological redox signaling.

Inflammatory RCD poses a major translational challenge because reduced inflammatory marker expression does not necessarily translate into durable neuronal protection. In MPTP‐induced PD mice, the selective NLRP3 inhibitor MCC950 attenuates inflammasome upregulation in the nigrostriatal pathway, improves motor performance, reduces dopaminergic neuronal loss, and suppresses microglial activation. MCC950 also blocks NLRP3 complex assembly, reduces IL‐1β maturation and release, and mitigates inflammation‐mediated neurotoxicity [340]. In ALS, mutant SOD1^G93A^ activates the microglial NLRP3 inflammasome, leading to Caspase‐1 cleavage and IL‐1β secretion. Genetic deletion of Nlrp3 or pretreatment with MCC950 suppresses this response, confirming the NLRP3 dependence of inflammasome‐mediated neuroinflammation [341]. Although these findings support inflammasome inhibition as a rational anti‐inflammatory strategy, future studies must determine whether inhibition of inflammasome signaling directly preserves vulnerable neuronal populations or mainly reduces secondary glial activation. Lower IL‐1β levels, reduced Caspase‐1 activation, or improvement in related inflammatory readouts may not be sufficient to predict long‐term functional benefit in heterogeneous ND populations.

Clinical experience with apoptosis‐directed therapy further illustrates why downstream cell death inhibition may fail when upstream pathogenic processes remain active. In NDs, apoptotic markers often arise within a broader pathological environment shaped by proteotoxic stress, mitochondrial dysfunction, axonal degeneration, and chronic glial activation. The key translational question is therefore not simply whether apoptosis contributes to neuronal loss, but whether selective suppression of apoptotic execution can alter disease progression after upstream stress programs have become self‐sustaining. This limitation is evident in prior clinical failures. TCH346, a potent anti apoptotic compound that protected dopaminergic neurons in experimental models, failed to show neuroprotective efficacy in a randomized controlled trial involving 301 patients with PD [342]. Minocycline, which has anti apoptotic and anti‐inflammatory properties in vitro, worsened clinical decline in a Phase III ALS trial, with treated patients deteriorating approximately 25% faster than placebo treated patients on ALS functional rating scale‐revised (ALSFRS‐R)‐based assessment [343]. These outcomes suggest that apoptosis inhibition alone is unlikely to modify disease trajectory when mitochondrial, proteostatic, and inflammatory injuries continue to drive neuronal vulnerability. In some settings, blocking apoptotic execution may also redirect injured cells toward more inflammatory or poorly cleared forms of degeneration. Apoptosis‐directed therapy should therefore be considered only when pathway activation is validated across disease stage and affected tissue, and preferably combined with interventions that reduce upstream mitochondrial, proteostatic, or inflammatory stress.

A similar principle applies to autophagy‐directed interventions, where the main translational issue is restoration of effective degradative flux rather than pathway activation alone. Autophagy can support neuronal survival by clearing damaged organelles and aggregation prone proteins, but therapeutic induction becomes problematic when lysosomal capacity, substrate recognition, or cellular energy status is already compromised. Clinical experience in ALS illustrates this distinction. Lithium, initially supported by pilot and preclinical evidence as an autophagy modulating agent, failed to slow ALS progression in a randomized, double blind, placebo controlled trial. The study was stopped for futility, with 22 of 40 lithium treated patients and 20 of 44 placebo treated patients reaching the primary event endpoint, hazard ratio 1.13, 95%CI 0.61–2.07 [344]. In the RAP‐ALS trial, rapamycin was well tolerated in 63 patients with ALS, but the primary endpoint of a greater than 30% increase in regulatory T cells was not achieved, and no clear clinical modification of disease trajectory was demonstrated [333]. Intravenous trehalose in the HEALEY ALS Platform Trial was also well tolerated but did not slow ALS progression or improve secondary clinical and biomarker outcomes [336]. Static markers such as LC3 or p62 are therefore insufficient as stand‐alone translational biomarkers, and future trials should incorporate dynamic measures of autophagic flux and lysosomal function.

These limitations do not reduce the importance of upstream disease modifying strategies. Rather, they indicate that cell death modulation should be combined with interventions that lower the initiating pathogenic burden. Beyond small molecule approaches, gene editing provides a strategy to reduce pathogenic protein load and attenuate downstream cell death. In R6/2 HD mice, dCas9–sgRNA targeting mHTT significantly extended rotarod performance and improved spontaneous activity, without adverse effects in wild‐type controls. Both dCas9–sgRNA and Cas9–sgRNA reduced striatal mHTT transcription, whereas dCas9 treated animals retained higher expression of functional markers and showed reduced neuronal apoptosis [345]. In SOD1^G93A^ ALS mice, delivery of SOD1 targeting sgRNA through AAV‐PHP.B or AAV‐PHP.eB achieved long‐term suppression of mutant protein in the CNS and spinal cord. A single neonatal intracerebroventricular injection extended survival by more than 110 days, whereas presymptomatic dosing extended survival by at least 170 days and reduced motor neuron loss and muscle atrophy [346]. Another studies have verified the long‐term safety and low off‐target rate of CRISPR/Cas9‐mediated editing in vivo, supporting its translational promise [347].

Gene editing studies also show why strong preclinical effects do not automatically ensure clinical feasibility [348]. Rodent models often involve early or presymptomatic intervention, high CNS transduction efficiency, genetically homogeneous disease backgrounds, and controlled delivery routes [349]. Human NDs, by contrast, are often diagnosed after substantial neuronal loss and are shaped by broader genetic, inflammatory, vascular, and environmental heterogeneity [350]. For cell death‐directed therapy, downstream RCD blockade may therefore be introduced too late if irreversible synaptic, axonal, or neuronal loss has already occurred [351].

Achieving sufficient drug exposure within disease relevant CNS regions remains a major challenge for RCD‐targeted therapy. Biomaterial‐based delivery systems, including nanocarriers and engineered exosomes, are increasingly being used to improve CNS delivery efficiency and lesion selective accumulation of therapeutic agents. Engineering BBB permeable nanocarriers or exosomes can increase CNS exposure and lesion selective accumulation of therapeutics. Functionalization of nanoparticles with transferrin or insulin receptor ligands has improved endothelial binding and brain uptake of anti Aβ agents [352, 353, 354]. Engineered exosomes are biocompatible, naturally derived vectors that can cross the BBB. Alvarez‐Erviti et al. generated neuron‐targeted exosomes loaded with beta‐site APP cleaving enzyme 1 (BACE1) siRNA and achieved an approximately 60% reduction in brain BACE1 mRNA and protein levels after intravenous administration, without peripheral toxicity [355]. This study showed that engineered exosomes can deliver nucleic acid therapeutics to neurons in vivo, supporting subsequent siRNA and miRNA‐based interventions in ND.

Cellular strategies may address another limitation of RCD‐directed therapy: persistent inflammatory amplification and defective clearance. Microglial replacement and engineering strategies have been developed to restore efficient clearance in the CNS. Short‐term administration of colony‐stimulating factor 1 receptor (CSF1R) inhibitors depletes endogenous microglia, and newly generated microglia repopulate the brain after drug withdrawal. In aged mice, this process reverses hippocampal spine loss, restores synaptic plasticity, and improves spatial memory [356, 357]. Transplantation of human hematopoietic stem cells expressing a CSF1R inhibitor resistant variant enables widespread human microglial engraftment in mouse brains, where the transplanted microglia maintain homeostatic characteristics and controlled inflammatory responses [358].

Peripherally or in vitro‐induced microglia like cells also show therapeutic potential. Transplantation of bone marrow‐derived‐induced microglia into the spinal cord of SOD1^G93A^ ALS mice results in long‐term survival of transplanted cells, improved motor function, prolonged survival, and reduced glial hyperactivation. Similarly, iPSC‐derived microglia migrate efficiently to injury sites, phagocytose neuronal debris, and acquire disease‐associated phenotypes [359, 360]. Compared with conventional drug‐based approaches, engineered microglial replacement may restore clearance capacity more persistently within diseased neural tissue and reduce chronic inflammation at the cellular source of neurodegenerative injury [359].

Clinical translation of RCD‐directed therapy will require a shift from broad pathway inhibition to disease stage and cell type informed intervention [361, 362]. Repeated failures of promising preclinical targets probably reflect several convergent barriers, including disease stage mismatch, insufficient evidence of target engagement in the human brain, weak linkage between pharmacodynamic biomarkers and neuronal survival, overreliance on acute toxin or highly penetrant transgenic models, lack of cell type‐specific biomarkers, and inadequate stratification of heterogeneous patient populations [349, 363, 364]. Future trials should incorporate modality‐specific pharmacodynamic readouts, including lipid peroxidation and labile iron markers for ferroptosis, p‐RIPK1 or MLKL‐related biomarkers for necroptosis, caspase activation signatures for apoptosis, inflammasome or gasdermin‐related readouts for pyroptosis, and dynamic autophagic flux instead of static LC3 or p62 measurements for autophagy [365, 366, 367, 368]. In advanced disease, RCD‐directed therapy is unlikely to succeed as uniform monotherapy. A more feasible translational strategy should combine early stage patient enrichment, CNS validated drug exposure, cell type aware biomarker monitoring, and multimodal interventions that reduce upstream proteotoxic, mitochondrial, inflammatory, and clearance‐related stress [361, 362, 369].

## 6 Current Limitations in Research

Although RCD has become important for understanding NDs, translating mechanistic findings into effective therapies remains difficult. Progress is limited by uncertainty in pathway attribution, disease heterogeneity, imperfect experimental models, insufficient biomarkers, and unresolved therapeutic stratification. These issues continue to complicate distinction between RCD pathways that actively participate in neuronal degeneration and those that arise as secondary features of chronic tissue injury.

### 6.1 Pathway‐Specific Mechanistic Uncertainty and Interpathway Crosstalk

A major limitation is that most RCD pathways are defined more clearly in experimental systems than in human neurodegenerative tissues. Apoptosis, necroptosis, pyroptosis, ferroptosis, ADCD, and other RCD modalities are classified by distinct molecular effectors, but their boundaries often become blurred in chronic neurodegeneration. Oxidative stress, mitochondrial dysfunction, proteostasis failure, and innate immune activation can coexist for long periods, making it difficult to assign neuronal injury to a single death pathway [18].

Ferroptosis illustrates this uncertainty. It is defined by iron‐dependent lipid peroxidation, depletion of cystine and glutathione antioxidant defenses, and impaired GPX4‐mediated detoxification of lipid peroxides [370]. However, the disease‐related triggers that initiate ferroptotic degeneration in NDs remain incompletely defined. Although iron chelation or lipid peroxidation inhibition improves neuronal survival in several models, these interventions may target only one component of a wider injury process involving mitochondrial dysfunction, neuroinflammation, protein aggregation, and metabolic stress [371]. It therefore remains difficult to determine whether ferroptosis is a primary driver, a downstream amplifier of oxidative injury, or a terminal phenotype shared by multiple pathological processes.

Crosstalk among RCD pathways further complicates therapeutic design. Inhibition of one pathway can redirect injured cells toward alternative death programs or reshape inflammatory signaling. For example, suppressing caspase‐dependent apoptosis may preserve plasma membrane integrity in some settings, but delayed clearance of damaged cells could intensify inflammation. Similarly, inhibition of necroptotic or pyroptotic signaling may reduce acute inflammatory injury, but excessive suppression could impair immune surveillance and debris clearance. These disease stage and cell type‐dependent effects suggest that targeting a single molecular node is unlikely to be sufficient unless the dominant death program and timing of intervention are clearly established.

### 6.2 Disease Stage, Brain‐Region, and Cell‐Type Heterogeneity

A second major limitation is the marked spatial and temporal heterogeneity of cell death signaling in NDs. Neurodegeneration does not follow a uniform death program across the brain. Different brain regions, neuronal subtypes, and glial populations show distinct vulnerabilities and may engage different RCD‐related pathways as disease progresses.

For instance, dopaminergic neurons in the substantia nigra pars compacta are particularly vulnerable in PD, and local iron accumulation supports a role for ferroptosis in selective nigrostriatal degeneration. AD, by contrast, involves broader iron dyshomeostasis, oxidative damage, astrocytic and microglial activation, tau pathology, and synaptic dysfunction across cortical and hippocampal regions. This complexity makes it difficult to attribute neurodegeneration to a single death pathway. Single nucleus transcriptomic analysis of 80,660 nuclei from the prefrontal cortex of 48 individuals with different degrees of AD pathology showed that the strongest disease‐associated transcriptional changes occur early and are highly cell type specific, whereas later changes are more globally associated with stress responses [189]. RCD‐related mechanisms should therefore be interpreted as stage dependent and cell type specific rather than static or homogeneous.

Besides, basal autophagy and stress adaptive proteostasis initially support neuronal survival by clearing damaged organelles and misfolded proteins. However, sustained or dysregulated autophagic flux may become maladaptive when lysosomal degradation is impaired and damaged mitochondria accumulate [372]. The biological meaning of pathway activation therefore differs across early compensation, active degeneration, and terminal tissue failure. This temporal shift helps explain why interventions effective in early experimental models may fail in patients with established neurodegeneration.

### 6.3 Limitations of Preclinical Models and Cross‐Species Extrapolation

Most mechanistic insights into RCD in NDs come from cell cultures, transgenic mice, toxin‐induced models, and zebrafish systems. These models are essential for defining molecular mechanisms, but they do not fully reproduce the cellular complexity, long disease latency, aging‐related changes, immune microenvironment, and metabolic regulation of the human nervous system.

Species differences are particularly important for brain organization and neuroimmune regulation. The human brain cannot be treated as a scaled up rodent brain. Differences in neuronal number, cortical organization, lifespan, and glial function influence how injury responses emerge and evolve [373]. Microglial biology also differs substantially between mice and humans. Single cell studies show that microglial states vary according to species, age, brain region, and disease environment, indicating that inflammatory and cell death‐related responses observed in mouse models may not directly predict human disease mechanisms [374]. Especially many AD mouse models rely on familial mutations and transgene overexpression, whereas most human AD cases are sporadic, late onset, and influenced by aging, vascular injury, metabolic dysfunction, *APOE* ε4 genotype, and immune status. This mismatch reduces the predictive value of interventions that are effective in animal models but do not address the molecular heterogeneity of human disease [375]. Positive preclinical results should therefore be interpreted as evidence of biological plausibility rather than definitive evidence of clinical efficacy.

### 6.4 Biomarker and Pharmacodynamic Monitoring Gaps

A major barrier is the absence of validated biomarkers that can dynamically distinguish RCD pathways in living patients. Current biomarkers are useful for detecting neurodegeneration, glial activation, or disease pathology, but they rarely identify which cell death program is active. Neurofilament light chain is a sensitive marker of neuroaxonal injury across neurological disorders, but it mainly reflects downstream structural damage rather than the upstream mechanisms leading to neuronal death [376]. GFAP provides diagnostic and prognostic information on astrocytic activation in AD, but it cannot determine whether glial reactivity is associated with apoptosis, ferroptosis, necroptosis, pyroptosis, or overlapping inflammatory injury [377].

Caspase activity, lipid peroxidation products, inflammatory cytokines, and oxidative stress markers can provide partial mechanistic information, but their clinical use is limited by tissue specificity, temporal instability, assay variability, and pathway overlap [378, 379]. At present, no validated clinical biomarker panel can reliably identify the dominant RCD mechanism in a given patient or disease stage [363, 380]. Neuroimaging also remains insufficient for real time pathway‐specific pharmacodynamic assessment. QSM can estimate regional iron burden, and PET can assess amyloid, tau, glucose metabolism, or neuroinflammation, but these methods do not directly measure active cell death [381, 382]. This gap limits early therapeutic evaluation and biologically informed patient stratification in clinical trials [361, 363].

### 6.5 Barriers to Clinical Translation and Therapeutic Stratification

Clinical translation is further limited by small sample sizes, short follow‐up, heterogeneous inclusion criteria, and insufficient biological stratification. The discrepancy between positive preclinical findings and unsuccessful clinical trials reflects a broader translational gap in ND research rather than the failure of individual therapeutic concepts. This gap is particularly evident in AD drug development. Across 413 AD clinical trials conducted between 2002 and 2012, the overall success rate was only 0.4%, corresponding to a 99.6% failure rate [383]. A subsequent analysis of Phase II and III AD trials identified 98 failed compounds from 2004 to 2021 and estimated that the Phase II/III success rate since the last novel approval in 2003 was approximately 2.0% [384]. These findings indicate that biological plausibility in experimental systems does not necessarily translate into clinical efficacy in chronic NDs.

Multiple factors contribute to this translational failure. Preclinical models frequently rely on acute toxic injury, young animals, or highly penetrant genetic manipulations, whereas human NDs evolve over decades under the influence of aging, vascular injury, metabolic dysfunction, genetic background, and chronic neuroimmune remodeling. The divergence between experimental models and human pathology has been widely recognized as a fundamental limitation in neurodegeneration research [385]. In addition, therapeutic intervention in animal studies is often initiated before or near the onset of pathology, whereas clinical trials usually enroll patients after substantial synaptic loss, neuronal depletion, glial reprogramming, and network‐level compensation have already occurred.

Uncertain CNS target engagement further weakens clinical translation. Biomarker changes detected in peripheral blood, cerebrospinal fluid, or neuroimaging do not necessarily indicate sufficient pharmacological activity in disease‐relevant brain regions or specific cell populations. This limitation is compounded by the BBB, which restricts brain entry for nearly all large‐molecule therapeutics and more than 98% of small‐molecule compounds [386]. Moreover, pharmacodynamic modulation of disease‐associated markers, including iron deposition, inflammatory mediators, or autophagy‐related proteins, may not correlate directly with neuronal survival or functional outcomes. The limitations of surrogate endpoints are well recognized in clinical research, especially when their causal relationship with true clinical outcomes has not been validated [387].

Iron‐targeted therapy provides a representative but not exclusive illustration of these barriers. In a 24‐month single‐blind study of 48 patients with probable AD, intramuscular desferrioxamine significantly slowed functional decline compared with controls, with group mean analysis yielding *p* = 0.03 [388]. However, this trial predated biomarker‐defined stratification and did not account for amyloid or tau status, limiting mechanistic interpretation. More recent deferiprone trials further show that target engagement does not necessarily translate into clinical benefit. In FAIRPARK‐II, deferiprone reduced nigrostriatal iron in newly diagnosed PD, but the Movement Disorder Society Unified Parkinson's Disease Rating Scale total score worsened more in the deferiprone group than in the placebo group, with a between‐group difference of 9.3 points, 95%CI 6.3–12.2, and *p* < 0.001 [245]. In amyloid‐confirmed mild cognitive impairment or early AD, deferiprone at 15 mg/kg twice daily reduced hippocampal QSM signals but accelerated cognitive decline [389]. These findings suggest that modulation of disease‐related imaging or biochemical markers does not necessarily indicate modification of disease‐driving mechanisms, particularly when intervention occurs outside the critical pathogenic window.

These translational barriers are broadly present across RCD‐targeted strategies and are particularly evident when surrogate molecular or imaging readouts are interpreted as proxies for true disease‐modifying effects. In NDs, this issue is further complicated by extensive crosstalk among RCD pathways. Inhibition of a single pathway may be counterbalanced by apoptosis, necroptosis, pyroptosis, ferroptosis, or autophagy–lysosomal dysfunction, thereby attenuating therapeutic efficacy in vivo. RCD‐targeted therapy therefore requires more precise patient stratification and mechanism‐based therapeutic design. For ferroptosis‐directed interventions, regional iron burden, lipid peroxidation status, GPX4‐dependent antioxidant capacity, *APOE* ε4 genotype, and inflammatory signatures may help identify responsive subpopulations. Similar principles apply to apoptosis, necroptosis, pyroptosis, and autophagy–lysosomal modulation strategies. Future clinical trial design should incorporate pathway‐specific pharmacodynamic biomarkers, validated CNS target engagement measures, disease‐stage stratification, and cell‐type‐relevant readouts. Rather than treating RCD pathways as universal therapeutic targets, future approaches should define which pathways are active, in which cellular compartments, and at which disease stages, and whether their modulation can meaningfully preserve neuronal structure and function.

### 6.6 Future Methodological Directions

Future studies need to move beyond isolated pathway analysis and adopt longitudinal, multimodal, and cell type‐specific strategies [361, 369]. Integration of multiomics, spatial transcriptomics, single cell and single nucleus sequencing, proteomics, metabolomics, and advanced imaging could map when and where specific RCD‐related programs emerge during disease progression [390, 391]. Such approaches may help distinguish early adaptive stress responses from irreversible cell death and identify the cell populations most relevant for intervention, including neurons, astrocytes, microglia, oligodendrocytes, and endothelial cells [369, 392].

Humanized model systems will also be important. Patient‐derived iPSCs, brain organoids, organ‐on‐chip systems, and coculture platforms incorporating neurons, glia, vascular elements, and immune components can improve mechanistic relevance [393, 394]. These systems still require validation against human postmortem tissue and longitudinal biomarker data [393]. Clinical trials should also incorporate biomarker enriched recruitment, pathway‐specific pharmacodynamic endpoints, longer follow up, and adaptive designs [361, 395]. Rather than applying RCD modulators broadly as disease modifying therapies, future studies should focus on molecularly stratified subgroups in which a specific cell death pathway is active and therapeutically accessible [363, 369].

Current limitations in RCD research reflect the biological complexity of chronic brain degeneration [369, 380]. Progress will require clearer interpretation of pathway crosstalk, regional and cellular heterogeneity, model limitations, biomarker insufficiency, and the gap between target engagement and clinical efficacy [363, 396]. Integrating mechanistic biology with patient level molecular profiling may help move RCD research from pathway description toward precision therapeutic development [361, 390].

## 7 Conclusion

This review shows that RCD is deeply involved in the pathogenesis and progression of NDs. Rather than acting as a single terminal event, RCD in neurodegeneration includes interconnected processes such as apoptosis, necroptosis, pyroptosis, ferroptosis, and ADCD. These pathways arise within a shared pathological environment marked by mitochondrial dysfunction, redox imbalance, proteostatic stress, lysosomal impairment, and persistent neuroinflammatory signaling. Because their activation may precede extensive neuronal loss, RCD should be viewed not only as a mechanism of terminal neuronal elimination, but also as a contributor to early neuronal vulnerability, synaptic dysfunction, glial activation, and disease progression.

The evidence discussed in this review further indicates that RCD pathways rarely operate independently. Apoptosis, necroptosis, pyroptosis, ferroptosis, and ADCD intersect through shared molecular checkpoints and convergent stress signals. Caspase‐8 influences the balance between apoptosis and necroptosis; mitochondrial injury promotes apoptotic signaling, inflammasome activation, oxidative stress, and defective mitophagy; inflammatory signaling amplifies pyroptotic, necroptotic, and ferroptotic injury; and impaired autophagy lysosomal function aggravates proteotoxic and mitochondrial stress. This biological overlap helps explain why inhibition of a single pathway is often insufficient to halt disease progression and why mixed death‐related phenotypes are commonly detected in neurodegenerative tissues.

Several RCD‐related mechanisms have translational potential. Targeting RIPK1 signaling, inflammasome activation, lipid peroxidation, iron dyshomeostasis, and autophagy lysosomal dysfunction has produced neuroprotective effects in experimental models. Some candidates have entered early clinical evaluation and have shown CNS exposure or pharmacodynamic pathway engagement. However, current clinical evidence remains insufficient to support RCD‐targeted therapy as a broadly effective disease modifying strategy. The gap between preclinical benefit and clinical outcome probably reflects disease heterogeneity, late intervention, inadequate patient stratification, incomplete target engagement in the human brain, and lack of pharmacodynamic biomarkers that directly link pathway modulation to neuronal preservation.

Future research needs to address the temporal and cellular specificity of RCD. The relative contribution of each pathway varies by disease type, brain region, disease stage, and affected cell population. Suppressing inflammatory or oxidative death‐related signaling may be more useful during early or active pathological phases, whereas benefit may decline after extensive neuronal and circuit loss. Because many RCD‐related molecules also participate in immune regulation, cellular quality control, synaptic remodeling, and tissue homeostasis, broad or prolonged inhibition may disrupt adaptive responses. Therapeutic strategies should therefore be guided by disease stage, affected cell type, and pathway activity.

Progress will require closer integration of mechanistic studies with clinically relevant biomarkers and longitudinal disease monitoring. Fluid biomarkers, molecular imaging, single cell profiling, spatial transcriptomics, and multiomics approaches may help define when and where specific RCD pathways are active during disease progression. These strategies could improve patient stratification, guide target selection, and support rational combination therapies targeting mitochondrial dysfunction, neuroinflammation, lipid peroxidation, proteostasis failure, and impaired debris clearance. RCD should therefore be understood as a dynamic pathological program embedded within broader neurodegenerative processes, rather than as a collection of isolated death pathways. A more precise understanding of this program may support the development of disease modifying therapies for AD, PD, ALS, HD, and related neurodegenerative disorders.

## Author Contributions

Tianjiao Li: Writing – original draft. Qian Zhang: figure preparation and manuscript proofreading. Yichen Wu: data curation. Ruixue Huang: study design and supervision, and Writing – review and editing. All authors have read and approved the final version of the manuscript.

## Funding

This work was supported by the The second batch of scientific experiment project of the Space Application System for Space Station Application and Development Engineering, General Department of Space Science and Application, Chinese Academy of Sciences(KJZ‐YY‐NSM0506).

## Conflicts of Interest

The authors declare no conflicts of interest.

## Ethics Statement

The authors have nothing to report.

## Data Availability

The authors have nothing to report.

## References

[1] A. M. Gorman , “Neuronal Cell Death in Neurodegenerative Diseases: Recurring Themes Around Protein Handling,” Journal of Cellular and Molecular Medicine 12, no. 6a (2008): 2263–2280. 18624755 10.1111/j.1582-4934.2008.00402.x PMC4514105

[2] D. Moujalled , A. Strasser , and J. R. Liddell , “Molecular Mechanisms of Cell Death in Neurological Diseases,” Cell Death and Differentiation 28, no. 7 (2021): 2029–2044. 34099897 10.1038/s41418-021-00814-y PMC8257776

[3] Global, Regional, and National Burden of Disorders Affecting the Nervous System, 1990–2021: A Systematic Analysis for the Global Burden of Disease Study 2021. *Lancet Neurology* 2024, 23(4): 344–381. 10.1016/S1474-4422(24)00038-3 PMC10949203 38493795

[4] Global, Regional, and National Burden of Parkinson's Disease, 1990–2016: A Systematic Analysis for the Global Burden of Disease Study 2016. *Lancet Neurology* 2018, 17(11): 939–953. 10.1016/S1474-4422(18)30295-3 PMC6191528 30287051

[5] D. G. Gadhave , V. V. Sugandhi , and S. K. Jha , “Neurodegenerative Disorders: Mechanisms of Degeneration and Therapeutic Approaches With Their Clinical Relevance,” Ageing Research Reviews 99 (2024): 102357. 38830548 10.1016/j.arr.2024.102357

[6] N. S. Erekat , “Programmed Cell Death in Diabetic Nephropathy: A Review of Apoptosis, Autophagy, and Necroptosis,” Medical Science Monitor 28 (2022): e937766. 35989481 10.12659/MSM.937766 PMC9462523

[7] R. M. Friedlander , “Apoptosis and Caspases in Neurodegenerative Diseases,” New England Journal of Medicine 348, no. 14 (2003): 1365–1375. 12672865 10.1056/NEJMra022366

[8] D. R. Thal , K. Gawor , and S. Moonen , “Regulated Cell Death and Its Role in Alzheimer's Disease and Amyotrophic Lateral Sclerosis,” Acta Neuropathologica 147, no. 1 (2024): 69. 38583129 10.1007/s00401-024-02722-0

[9] J. Li , F. Cao , H. L. Yin , et al., “Ferroptosis: Past, Present and Future,” Cell Death & Disease 11, no. 2 (2020): 88. 32015325 10.1038/s41419-020-2298-2 PMC6997353

[10] D. Tang , X. Chen , R. Kang , et al., “Ferroptosis: Molecular Mechanisms and Health Implications,” Cell Research 31, no. 2 (2021): 107–125. 33268902 10.1038/s41422-020-00441-1 PMC8026611

[11] Y. Fei and Y. Ding , “The Role of Ferroptosis in Neurodegenerative Diseases,” Front Cell Neurosci 18 (2024): 1475934. 39473490 10.3389/fncel.2024.1475934 PMC11518764

[12] Q. Wang , S. Yang , X. Zhang , et al., “Inflammasomes in Neurodegenerative Diseases,” Transl Neurodegener 13, no. 1 (2024): 65. 39710713 10.1186/s40035-024-00459-0 PMC11665095

[13] R. A. Nixon , “Autophagy‐lysosomal‐associated Neuronal Death in Neurodegenerative Disease,” Acta Neuropathologica 148, no. 1 (2024): 42. 39259382 10.1007/s00401-024-02799-7 PMC11418399

[14] L. Cai , Q. Fan , R. Pang , et al., “Microglia Programmed Cell Death in Neurodegenerative Diseases and CNS Injury,” Apoptosis 30, no. 1‐2 (2025): 446–465. 39656359 10.1007/s10495-024-02041-5 PMC11799081

[15] L. Tang , S. Liu , S. Li , et al., “Induction Mechanism of Ferroptosis, Necroptosis, and Pyroptosis: A Novel Therapeutic Target in Nervous System Diseases,” International Journal of Molecular Sciences 24, no. 12 (2023): 10127. 37373274 10.3390/ijms241210127 PMC10299044

[16] G. Eskander , S. G. Abdelhamid , S. A. Wahdan , et al., “Insights on the Crosstalk Among Different Cell Death Mechanisms,” Cell Death Discov 11, no. 1 (2025): 56. 39929794 10.1038/s41420-025-02328-9 PMC11811070

[17] J. Cui , S. Zhao , Y. Li , et al., “Regulated Cell Death: Discovery, Features and Implications for Neurodegenerative Diseases,” Cell Communication and Signaling 19, no. 1 (2021): 120. 34922574 10.1186/s12964-021-00799-8 PMC8684172

[18] L. Galluzzi , I. Vitale , S. A. Aaronson , et al., “Molecular Mechanisms of Cell Death: Recommendations of the Nomenclature Committee on Cell Death 2018,” Cell Death and Differentiation 25, no. 3 (2018): 486–541. 29362479 10.1038/s41418-017-0012-4 PMC5864239

[19] J. F. Kerr , A. H. Wyllie , and A. R. Currie , “Apoptosis: A Basic Biological Phenomenon With Wide‐ranging Implications in Tissue Kinetics,” British Journal of Cancer 26, no. 4 (1972): 239–257. 4561027 10.1038/bjc.1972.33 PMC2008650

[20] P. Vandenabeele , L. Galluzzi , B. T. Vanden , et al., “Molecular Mechanisms of Necroptosis: An Ordered Cellular Explosion,” Nature Reviews Molecular Cell Biology 11, no. 10 (2010): 700–714. 20823910 10.1038/nrm2970

[21] N. Kayagaki , I. B. Stowe , B. L. LEE , et al., “Caspase‐11 Cleaves Gasdermin D for Non‐canonical Inflammasome Signalling,” Nature 526, no. 7575 (2015): 666–671. 26375259 10.1038/nature15541

[22] S. J. Dixon , K. M. Lemberg , M. R. Lamprecht , et al., “Ferroptosis: An Iron‐dependent Form of Nonapoptotic Cell Death,” Cell 149, no. 5 (2012): 1060–1072. 22632970 10.1016/j.cell.2012.03.042 PMC3367386

[23] Y. Liu , S. Shoji‐Kawata , R. M. Sumpter JR , et al., “Autosis Is a Na+,K+‐ATPase‐regulated Form of Cell Death Triggered by Autophagy‐inducing Peptides, Starvation, and Hypoxia‐ischemia,” PNAS 110, no. 51 (2013): 20364–20371. 24277826 10.1073/pnas.1319661110 PMC3870705

[24] M. Mustafa , R. Ahmad , I. Q. Tantry , et al., “Apoptosis: A Comprehensive Overview of Signaling Pathways, Morphological Changes, and Physiological Significance and Therapeutic Implications,” Cells 13, no. 22 (2024): 1838. 39594587 10.3390/cells13221838 PMC11592877

[25] M. D'amelio , M. Sheng , and F. Cecconi , “Caspase‐3 in the central Nervous System: Beyond Apoptosis,” Trends in Neuroscience 35, no. 11 (2012): 700–709. 10.1016/j.tins.2012.06.004 22796265

[26] J. L. Franklin , “Redox Regulation of the Intrinsic Pathway in Neuronal Apoptosis,” Antioxid Redox Signaling 14, no. 8 (2011): 1437–1448. 10.1089/ars.2010.3596 PMC3061193 20812874

[27] P. Goel , S. Chakrabarti , K. Goel , et al., “Neuronal Cell Death Mechanisms in Alzheimer's Disease: An Insight,” Front Mol Neurosci 15 (2022): 937133. 36090249 10.3389/fnmol.2022.937133 PMC9454331

[28] S. A. Susin , H. K. Lorenzo , N. Zamzami , et al., “Molecular Characterization of Mitochondrial Apoptosis‐inducing Factor,” Nature 397, no. 6718 (1999): 441–446. 9989411 10.1038/17135

[29] X. Wang , C. Yang , J. Chai , et al., “Mechanisms of AIF‐mediated Apoptotic DNA Degradation in Caenorhabditis elegans,” Science 298, no. 5598 (2002): 1587–1592. 12446902 10.1126/science.1076194

[30] P. Wójcik , M. K. Jastrzębski , and A. Zięba , “Caspases in Alzheimer's Disease: Mechanism of Activation, Role, and Potential Treatment,” Molecular Neurobiology 61, no. 7 (2024): 4834–4853. 38135855 10.1007/s12035-023-03847-1 PMC11236938

[31] M. D'amelio , V. Cavallucci , and F. Cecconi , “Neuronal Caspase‐3 Signaling: Not Only Cell Death,” Cell Death and Differentiation 17, no. 7 (2010): 1104–1114. 19960023 10.1038/cdd.2009.180

[32] E. Radi , P. Formichi , C. Battisti , et al., “Apoptosis and Oxidative Stress in Neurodegenerative Diseases,” Journal of Alzheimer's Disease 42, no. 3 (2014): S125–52. 10.3233/JAD-132738 25056458

[33] K. Newton , K. E. Wickliffe , D. L. Dugger , et al., “Cleavage of RIPK1 by Caspase‐8 Is Crucial for Limiting Apoptosis and Necroptosis,” Nature 574, no. 7778 (2019): 428–431. 31511692 10.1038/s41586-019-1548-x

[34] M. Pasparakis and P. Vandenabeele , “Necroptosis and Its Role in Inflammation,” Nature 517, no. 7534 (2015): 311–320. 25592536 10.1038/nature14191

[35] K. Newton , “RIPK1 and RIPK3: Critical Regulators of Inflammation and Cell Death,” Trends in Cell Biology 25, no. 6 (2015): 347–353. 25662614 10.1016/j.tcb.2015.01.001

[36] L. Sun , H. Wang , Z. Wang , et al., “Mixed Lineage Kinase Domain‐Like Protein Mediates Necrosis Signaling Downstream of RIP3 Kinase,” Cell 148, no. 1‐2 (2012): 213–227. 22265413 10.1016/j.cell.2011.11.031

[37] J. M. Murphy , P. E. Czabotar , J. M. Hildebrand , et al., “The Pseudokinase MLKL Mediates Necroptosis via a Molecular Switch Mechanism,” Immunity 39, no. 3 (2013): 443–453. 24012422 10.1016/j.immuni.2013.06.018

[38] S. Yoon , A. Kovalenko , K. Bogdanov , et al., “MLKL, the Protein That Mediates Necroptosis, Also Regulates Endosomal Trafficking and Extracellular Vesicle Generation,” Immunity 47, no. 1 (2017): 51–65.e7. 28666573 10.1016/j.immuni.2017.06.001

[39] X. Wu , L. E. Nagy , and J. Gautheron , “Mediators of Necroptosis: From Cell Death to Metabolic Regulation,” EMBO Molecular Medicine 16, no. 2 (2024): 219–237. 38195700 10.1038/s44321-023-00011-z PMC10897313

[40] Y. Ito , D. Ofengeim , A. Najafov , et al., “RIPK1 mediates Axonal Degeneration by Promoting Inflammation and Necroptosis in ALS,” Science 353, no. 6299 (2016): 603–608. 27493188 10.1126/science.aaf6803 PMC5444917

[41] S. Dominguez , E. Varfolomeev , R. Brendza , et al., “Genetic Inactivation of RIP1 Kinase Does Not Ameliorate Disease in a Mouse Model of ALS,” Cell Death and Differentiation 28, no. 3 (2021): 915–931. 32994544 10.1038/s41418-020-00625-7 PMC7937687

[42] Y. Chen , J. Lin , Y. Zhao , et al., “Toll‐Like Receptor 3 (TLR3) Regulation Mechanisms and Roles in Antiviral Innate Immune Responses,” J Zhejiang Univ Sci B 22, no. 8 (2021): 609–632. 34414698 10.1631/jzus.B2000808 PMC8377577

[43] T. Kuriakose , S. M. Man , and R. K. Malireddi , “ZBP1/DAI Is an Innate Sensor of Influenza Virus Triggering the NLRP3 Inflammasome and Programmed Cell Death Pathways,” Science Immunology 1, no. 2 (2016): aag2045. 27917412 10.1126/sciimmunol.aag2045 PMC5131924

[44] K. Thapa , H. Khan , T. G. Singh , et al., “Traumatic Brain Injury: Mechanistic Insight on Pathophysiology and Potential Therapeutic Targets,” Journal of Molecular Neuroscience 71, no. 9 (2021): 1725–1742. 33956297 10.1007/s12031-021-01841-7

[45] B. T. Cookson and M. A. Brennan , “Pro‐inflammatory Programmed Cell Death,” Trends in Microbiology 9, no. 3 (2001): 113–114. 11303500 10.1016/s0966-842x(00)01936-3

[46] T. Bergsbaken , S. L. Fink , and B. T. Cookson , “Pyroptosis: Host Cell Death and Inflammation,” Nature Reviews Microbiology 7, no. 2 (2009): 99–109. 19148178 10.1038/nrmicro2070 PMC2910423

[47] H. Guo , J. B. Callaway , and J. P. Ting , “Inflammasomes: Mechanism of Action, Role in Disease, and Therapeutics,” Nature Medicine 21, no. 7 (2015): 677–687. 10.1038/nm.3893 PMC4519035 26121197

[48] P. Broz and V. M. Dixit , “Inflammasomes: Mechanism of Assembly, Regulation and Signalling,” Nature Reviews Immunology 16, no. 7 (2016): 407–420. 10.1038/nri.2016.58 27291964

[49] J. Shi , Y. Zhao , Y. Wang , et al., “Inflammatory Caspases Are Innate Immune Receptors for Intracellular LPS,” Nature 514, no. 7521 (2014): 187–192. 25119034 10.1038/nature13683

[50] J. Ding , K. Wang , W. Liu , et al., “Pore‐forming Activity and Structural Autoinhibition of the gasdermin family,” Nature 535, no. 7610 (2016): 111–116. 27281216 10.1038/nature18590

[51] S. XIA , Z. ZHANG , and V. G. MAGUPALLI , “Gasdermin D Pore Structure Reveals Preferential Release of Mature Interleukin‐1,” Nature 593, no. 7860 (2021): 607–611. 33883744 10.1038/s41586-021-03478-3 PMC8588876

[52] S. Rühl , K. Shkarina , B. Demarco , et al., “ESCRT‐dependent Membrane Repair Negatively Regulates Pyroptosis Downstream of GSDMD Activation,” Science 362, no. 6417 (2018): 956–960. 30467171 10.1126/science.aar7607

[53] M. T. Heneka , M. P. Kummer , A. Stutz , et al., “NLRP3 is Activated in Alzheimer's Disease and Contributes to Pathology in APP/PS1 Mice,” Nature 493, no. 7434 (2013): 674–678. 23254930 10.1038/nature11729 PMC3812809

[54] C. Ising , C. Venegas , S. Zhang , et al., “NLRP3 inflammasome Activation Drives Tau Pathology,” Nature 575, no. 7784 (2019): 669–673. 31748742 10.1038/s41586-019-1769-z PMC7324015

[55] W. Rui , S. Li , H. Xiao , et al., “Baicalein Attenuates Neuroinflammation by Inhibiting NLRP3/Caspase‐1/GSDMD Pathway in MPTP Induced Mice Model of Parkinson's Disease,” The International Journal of Neuropsychopharmacology 23, no. 11 (2020): 762–773. 32761175 10.1093/ijnp/pyaa060 PMC7745250

[56] R. Zheng , Y. Yan , S. Dai , et al., “ASC Specks Exacerbate α‑Synuclein Pathology via Amplifying NLRP3 Inflammasome Activities,” J Neuroinflammation 20, no. 1 (2023): 26. 36740674 10.1186/s12974-023-02709-w PMC9899382

[57] P. Huang , Z. Zhang , P. Zhang , et al., “TREM2 Deficiency Aggravates NLRP3 Inflammasome Activation and Pyroptosis in MPTP‐Induced Parkinson's Disease Mice and LPS‐Induced BV2 Cells,” Molecular Neurobiology 61, no. 5 (2024): 2590–2605. 37917301 10.1007/s12035-023-03713-0 PMC11043123

[58] F. Moradi and T. Mokhtari , “Role of NLRP3 Inflammasome in Chronic Pain and Alzheimer's Disease—A Review,” Journal of Biochemical and Molecular Toxicology 39, no. 2 (2025): e70071. 39853846 10.1002/jbt.70071 PMC11798427

[59] N. Panicker , T. I. Kam , H. Wang , et al., “Neuronal NLRP3 Is a Parkin Substrate That Drives Neurodegeneration in Parkinson's Disease,” Neuron 110, no. 15 (2022): 2422–2437.e9. 35654037 10.1016/j.neuron.2022.05.009 PMC9357148

[60] X. Zhang , Y. Zhang , B. Wang , et al., “Pyroptosis‐mediator GSDMD Promotes Parkinson's Disease Pathology via Microglial Activation and Dopaminergic Neuronal Death,” Brain, Behavior, and Immunity 119 (2024): 129–145. 38552923 10.1016/j.bbi.2024.03.038

[61] G. Gunner , H. Basu , Y. Lu , et al., “Gasdermin D Is Activated but Does Not Drive Neurodegeneration in SOD1(G93A) Model of ALS: Implications for Targeting Pyroptosis,” Neurobiology of Disease 214 (2025): 107048. 40759286 10.1016/j.nbd.2025.107048 PMC12412056

[62] F. L. Anderson , K. E. Biggs , B. E. Rankin , et al., “NLRP3 inflammasome in Neurodegenerative Disease,” Transl Res 252 (2023): 21–33. 35952982 10.1016/j.trsl.2022.08.006 PMC10614656

[63] X. Wu , T. Wan , X. Gao , et al., “Microglia Pyroptosis: A Candidate Target for Neurological Diseases Treatment,” Frontiers in neuroscience 16 (2022): 922331. 35937897 10.3389/fnins.2022.922331 PMC9354884

[64] Y. Wang and T. D. Kanneganti , “From Pyroptosis, Apoptosis and Necroptosis to PANoptosis: A Mechanistic Compendium of Programmed Cell Death Pathways,” Computational and Structural Biotechnology Journal 19 (2021): 4641–4657. 34504660 10.1016/j.csbj.2021.07.038 PMC8405902

[65] B. R. Stockwell , “Ferroptosis Turns 10: Emerging Mechanisms, Physiological Functions, and Therapeutic Applications,” Cell 185, no. 14 (2022): 2401–2421. 35803244 10.1016/j.cell.2022.06.003 PMC9273022

[66] X. Jiang , B. R. Stockwell , and M. Conrad , “Ferroptosis: Mechanisms, Biology and Role in Disease,” Nature Reviews Molecular Cell Biology 22, no. 4 (2021): 266–282. 33495651 10.1038/s41580-020-00324-8 PMC8142022

[67] Y. Xie , W. Hou , X. Song , et al., “Ferroptosis: Process and Function,” Cell Death and Differentiation 23, no. 3 (2016): 369–379. 26794443 10.1038/cdd.2015.158 PMC5072448

[68] C. O. Reichert , F. A. De Freitas , J. Sampaio‐Silva , et al., “Ferroptosis Mechanisms Involved in Neurodegenerative Diseases,” International Journal of Molecular Sciences 21, no. 22 (2020): 8765. 33233496 10.3390/ijms21228765 PMC7699575

[69] M. Ou , Y. Jiang , Y. Ji , et al., “Role and Mechanism of Ferroptosis in Neurological Diseases,” Mol Metab 61 (2022): 101502. 35447365 10.1016/j.molmet.2022.101502 PMC9170779

[70] L. Chen , W. S. Hambright , R. Na , et al., “Ablation of the Ferroptosis Inhibitor Glutathione Peroxidase 4 in Neurons Results in Rapid Motor Neuron Degeneration and Paralysis,” Journal of Biological Chemistry 290, no. 47 (2015): 28097–28106. 26400084 10.1074/jbc.M115.680090 PMC4653669

[71] S. K. Ryan , M. Zelic , Y. Han , et al., “Microglia Ferroptosis Is Regulated by SEC24B and Contributes to Neurodegeneration,” Nature neuroscience 26, no. 1 (2023): 12–26. 36536241 10.1038/s41593-022-01221-3 PMC9829540

[72] Y. G. Fan , R. L. Ge , H. Ren , et al., “Astrocyte‐derived Lactoferrin Inhibits Neuronal Ferroptosis by Reducing Iron Content and GPX4 Degradation in APP/PS1 Transgenic Mice,” Pharmacological Research 209 (2024): 107404. 39306020 10.1016/j.phrs.2024.107404

[73] T. Hara , K. Nakamura , M. Matsui , et al., “Suppression of Basal Autophagy in Neural Cells Causes Neurodegenerative Disease in Mice,” Nature 441, no. 7095 (2006): 885–889. 16625204 10.1038/nature04724

[74] S. Tang , J. Zhang , J. Chen , et al., “Ferroptosis in Neurodegenerative Diseases: Potential Mechanisms of Exercise Intervention,” Frontiers in Cell and Developmental Biology 13 (2025): 1622544. 40661149 10.3389/fcell.2025.1622544 PMC12256474

[75] A. K. H. Stavoe and E. L. F. Holzbaur , “Autophagy in Neurons,” Annual Review of Cell and Developmental Biology 35 (2019): 477–500. 10.1146/annurev-cellbio-100818-125242 PMC6996145 31340124

[76] M. Komatsu , S. Waguri , T. Chiba , et al., “Loss of Autophagy in the central Nervous System Causes Neurodegeneration in Mice,” Nature 441, no. 7095 (2006): 880–884. 16625205 10.1038/nature04723

[77] J. J. Collier , F. Suomi , M. Oláhová , et al., “Emerging Roles of ATG7 in human Health and Disease,” EMBO Molecular Medicine 13, no. 12 (2021): e14824. 34725936 10.15252/emmm.202114824 PMC8649875

[78] Y. Liu and B. Levine , “Autosis and Autophagic Cell Death: The Dark Side of Autophagy,” Cell Death and Differentiation 22, no. 3 (2015): 367–376. 25257169 10.1038/cdd.2014.143 PMC4326571

[79] D. Denton and S. Kumar , “Autophagy‐dependent Cell Death,” Cell Death and Differentiation 26, no. 4 (2019): 605–616. 30568239 10.1038/s41418-018-0252-y PMC6460387

[80] S. Aits and M. Jäättelä , “Lysosomal Cell Death at a Glance,” Journal of Cell Science 126, no. Pt 9 (2013): 1905–1912. 23720375 10.1242/jcs.091181

[81] T. Yoshida , T. Yoshida , H. Noma , et al., “Diagnostic Accuracy of Point‐of‐care Ultrasound for Shock: A Systematic Review and Meta‐analysis,” Critical Care 27, no. 1 (2023): 200. 37231510 10.1186/s13054-023-04495-6 PMC10214599

[82] R. A. Nixon , “Autophagy, Amyloidogenesis and Alzheimer Disease,” Journal of Cell Science 120, no. Pt 23 (2007): 4081–4091. 18032783 10.1242/jcs.019265

[83] J. H. Lee , D. S. Yang , C. N. Goulbourne , et al., “Faulty Autolysosome Acidification in Alzheimer's disease Mouse Models Induces Autophagic Build‐up of Aβ in Neurons, Yielding Senile Plaques,” Nature Neuroscience 25, no. 6 (2022): 688–701. 35654956 10.1038/s41593-022-01084-8 PMC9174056

[84] J. Kim and K. L. Guan , “mTOR as a central Hub of Nutrient Signalling and Cell Growth,” Nature Cell Biology 21, no. 1 (2019): 63–71. 30602761 10.1038/s41556-018-0205-1

[85] Y. Zhu , C. Wang , M. Yu , et al., “ULK1 and JNK Are Involved in Mitophagy Incurred by LRRK2 G2019S Expression,” Protein Cell 4, no. 9 (2013): 711–721. 27023913 10.1007/s13238-013-3910-3 PMC4875534

[86] J. Obergasteiger , G. Frapporti , G. Lamonaca , et al., “Kinase Inhibition of G2019S‐LRRK2 Enhances Autolysosome Formation and Function to Reduce Endogenous Alpha‐synuclein Intracellular Inclusions,” Cell Death Discov 6 (2020): 45. 32550012 10.1038/s41420-020-0279-y PMC7280235

[87] Buck S A and L. H. Sanders , “LRRK2‐mediated Mitochondrial Dysfunction in Parkinson's Disease,” Biochemical Journal 482, no. 11 (2025): 721–739. 40440058 10.1042/BCJ20253062 PMC12181902

[88] B. Boland , A. Kumar , S. Lee , et al., “Autophagy Induction and Autophagosome Clearance in Neurons: Relationship to Autophagic Pathology in Alzheimer's Disease,” Journal of Neuroscience 28, no. 27 (2008): 6926–6937. 18596167 10.1523/JNEUROSCI.0800-08.2008 PMC2676733

[89] M. E. Ahmed , S. Iyer , R. Thangavel , et al., “Co‐Localization of Glia Maturation Factor With NLRP3 Inflammasome and Autophagosome Markers in Human Alzheimer's Disease Brain,” Journal of Alzheimer's Disease 60, no. 3 (2017): 1143–1160. 10.3233/JAD-170634 PMC5770146 28984607

[90] A. M. Cuervo , L. Stefanis , R. Fredenburg , et al., “Impaired Degradation of Mutant Alpha‐synuclein by Chaperone‐mediated Autophagy,” Science 305, no. 5688 (2004): 1292–1295. 15333840 10.1126/science.1101738

[91] A. Navarro‐Romero , I. Fernandez‐Gonzalez , J. Riera , et al., “Lysosomal Lipid Alterations Caused by Glucocerebrosidase Deficiency Promote Lysosomal Dysfunction, Chaperone‐mediated‐autophagy Deficiency, and Alpha‐synuclein Pathology,” NPJ Parkinsons Dis 8, no. 1 (2022): 126. 36202848 10.1038/s41531-022-00397-6 PMC9537323

[92] C. Settembre , C. R. De , G. Mansueto , et al., “TFEB Controls Cellular Lipid Metabolism Through a Starvation‐induced Autoregulatory Loop,” Nature Cell Biology 15, no. 6 (2013): 647–658. 23604321 10.1038/ncb2718 PMC3699877

[93] V. A. Polito , H. Li , H. Martini‐Stoica , et al., “Selective Clearance of Aberrant Tau Proteins and Rescue of Neurotoxicity by Transcription Factor EB,” EMBO Molecular Medicine 6, no. 9 (2014): 1142–1160. 25069841 10.15252/emmm.201303671 PMC4197862

[94] L. Neumann , C. Pforr , J. Beaudouin , et al., “Dynamics Within the CD95 Death‐inducing Signaling Complex Decide Life and Death of Cells,” Molecular Systems Biology 6 (2010): 352. 20212524 10.1038/msb.2010.6 PMC2858442

[95] M. Muzio , A. M. Chinnaiyan , F. C. Kischkel , et al., “FLICE, a Novel FADD‐homologous ICE/CED‐3‐Like Protease, Is Recruited to the CD95 (Fas/APO‐1) Death–inducing Signaling Complex,” Cell 85, no. 6 (1996): 817–827. 8681377 10.1016/s0092-8674(00)81266-0

[96] A. Oberst , C. P. Dillon , R. Weinlich , et al., “Catalytic Activity of the Caspase‐8‐FLIP(L) Complex Inhibits RIPK3‐dependent Necrosis,” Nature 471, no. 7338 (2011): 363–367. 21368763 10.1038/nature09852 PMC3077893

[97] D. W. Zhang , J. Shao , J. Lin , et al., “RIP3, an Energy Metabolism Regulator That Switches TNF‐induced Cell Death From Apoptosis to Necrosis,” Science 325, no. 5938 (2009): 332–336. 19498109 10.1126/science.1172308

[98] W. J. Kaiser , J. W. Upton , A. B. Long , et al., “RIP3 mediates the Embryonic Lethality of Caspase‐8‐deficient Mice,” Nature 471, no. 7338 (2011): 368–372. 21368762 10.1038/nature09857 PMC3060292

[99] Cho Y S , S. Challa , D. Moquin , et al., “Phosphorylation‐driven Assembly of the RIP1‐RIP3 Complex Regulates Programmed Necrosis and Virus‐induced Inflammation,” Cell 137, no. 6 (2009): 1112–1123. 19524513 10.1016/j.cell.2009.05.037 PMC2727676

[100] E. Deroo , M. Khoury , T. Zhou , et al., “Investigating the Role of Receptor Interacting Protein Kinase 3 in Venous Thrombosis,” JVS Vasc Sci 3 (2022): 365–378. 36568281 10.1016/j.jvssci.2022.09.002 PMC9772854

[101] Y. Q. Gan , Y. W. Cai , and X. W. Liang , “Impaired NLRP3 Inflammasome Signaling Diverts Pyroptotic to Apoptotic Caspase Activation in Macrophages,” Frontiers in immunology 16 (2025): 1631152. 41208996 10.3389/fimmu.2025.1631152 PMC12591955

[102] C. Picon , A. Jayaraman , R. James , et al., “Neuron‐specific Activation of Necroptosis Signaling in Multiple Sclerosis Cortical Grey Matter,” Acta Neuropathologica 141, no. 4 (2021): 585–604. 33569629 10.1007/s00401-021-02274-7 PMC7952371

[103] R. J. Youle and A. Strasser , “The BCL‐2 Protein family: Opposing Activities That Mediate Cell Death,” Nature Reviews Molecular Cell Biology 9, no. 1 (2008): 47–59. 18097445 10.1038/nrm2308

[104] S. W. Tait and D. R. Green , “Mitochondria and Cell Death: Outer Membrane Permeabilization and Beyond,” Nature Reviews Molecular Cell Biology 11, no. 9 (2010): 621–632. 20683470 10.1038/nrm2952

[105] M. C. Wei , W. X. Zong , E. H. Cheng , et al., “Proapoptotic BAX and BAK: A Requisite Gateway to Mitochondrial Dysfunction and Death,” Science 292, no. 5517 (2001): 727–730. 11326099 10.1126/science.1059108 PMC3049805

[106] R. Zhou , A. S. Yazdi , P. Menu , et al., “A Role for Mitochondria in NLRP3 Inflammasome Activation,” Nature 469, no. 7329 (2011): 221–225. 21124315 10.1038/nature09663

[107] K. Senkevich and Z. Gan‐Or , “Autophagy Lysosomal Pathway Dysfunction in Parkinson's disease; Evidence From human Genetics,” Parkinsonism & related disorders 73 (2020): 60–71. 31761667 10.1016/j.parkreldis.2019.11.015

[108] H. Sandoval , P. Thiagarajan , S. K. Dasgupta , et al., “Essential Role for Nix in Autophagic Maturation of Erythroid Cells,” Nature 454, no. 7201 (2008): 232–235. 18454133 10.1038/nature07006 PMC2570948

[109] A. M. Pickrell and J. Youle R , “The Roles of PINK1, Parkin, and Mitochondrial Fidelity in Parkinson's Disease,” Neuron 85, no. 2 (2015): 257–273. 25611507 10.1016/j.neuron.2014.12.007 PMC4764997

[110] J. Obergasteiger , A.‐M. Castonguay , S. Pizzi , et al., “The Small GTPase Rit2 Modulates LRRK2 Kinase Activity, Is Required for Lysosomal Function and Protects Against Alpha‐synuclein Neuropathology,” npj Parkinson's Disease 9, no. 1 (2023): 44. 10.1038/s41531-023-00484-2 PMC10042831 36973269

[111] J. Li , D. Yang , Z. Li , et al., “PINK1/Parkin‐mediated Mitophagy in Neurodegenerative Diseases,” Ageing research reviews 84 (2023): 101817. 36503124 10.1016/j.arr.2022.101817

[112] M. T. HENEKA , M. J. CARSON , K. J. EL , et al., “Neuroinflammation in Alzheimer's Disease,” The Lancet Neurology 14, no. 4 (2015): 388–405. 25792098 10.1016/S1474-4422(15)70016-5 PMC5909703

[113] J. Shi , Y. Zhao , K. Wang , et al., “Cleavage of GSDMD by Inflammatory Caspases Determines Pyroptotic Cell Death,” Nature 526, no. 7575 (2015): 660–665. 26375003 10.1038/nature15514

[114] S. A. Conos , K. W. Chen , N. D. De , et al., “Active MLKL Triggers the NLRP3 Inflammasome in a Cell‐intrinsic Manner,” Proceedings of the National Academy of Sciences 114, no. 6 (2017): E961–E9. 10.1073/pnas.1613305114 PMC5307433 28096356

[115] J. Cai , W. Ji , P. Liu , et al., “Endothelial NLRP3‐mediated Pyroptosis Induces Blood‐brain Barrier and Neuronal Damage in Huntington's disease Models,” Journal of Pharmacological Sciences 159, no. 4 (2025): 256–267. 41241436 10.1016/j.jphs.2025.09.003

[116] A. J. P. Friedmann , M. Schneider , B. Proneth , et al., “Inactivation of the Ferroptosis Regulator Gpx4 Triggers Acute Renal Failure in Mice,” Nature Cell Biology 16, no. 12 (2014): 1180–1191. 25402683 10.1038/ncb3064 PMC4894846

[117] L. Lv , Y. Wang , X. Lv , et al., “Involvement of HMGB1‐mediated Ferroptosis in Systemic Diseases,” Frontiers in Cell and Developmental Biology 13 (2025): 1676941. 41113461 10.3389/fcell.2025.1676941 PMC12531243

[118] T. Wang , D. Tomas , N. D. Perera , et al., “Ferroptosis Mediates Selective Motor Neuron Death in Amyotrophic Lateral sclerosis,” Cell Death and Differentiation 29, no. 6 (2022): 1187–1198. 34857917 10.1038/s41418-021-00910-z PMC9177596

[119] K. Chen , X. Jiang , M. Wu , et al., “Ferroptosis, a Potential Therapeutic Target in Alzheimer's Disease,” Frontiers in cell and developmental biology 9 (2021): 704298. 34422824 10.3389/fcell.2021.704298 PMC8374166

[120] G. Á. Tannahill , A. Curtis , J. Adamik , et al., “Succinate Is an Inflammatory Signal That Induces IL‐1β Through HIF‐1α,” Nature 496, no. 7444 (2013): 238–242. 23535595 10.1038/nature11986 PMC4031686

[121] P.‐S. Liu , H. Wang , X. Li , et al., “α‐ketoglutarate Orchestrates Macrophage Activation Through Metabolic and Epigenetic Reprogramming,” Nature immunology 18, no. 9 (2017): 985–994. 28714978 10.1038/ni.3796

[122] L. Leng , Z. Yuan , R. Pan , et al., “Microglial Hexokinase 2 Deficiency Increases ATP Generation Through Lipid Metabolism Leading to β‐amyloid Clearance,” Nature metabolism 4, no. 10 (2022): 1287–1305. 10.1038/s42255-022-00643-4 36203054

[123] Y. Hu , K. Cao , F. Wang , et al., “Dual Roles of Hexokinase 2 in Shaping Microglial Function by Gating Glycolytic Flux and Mitochondrial Activity,” Nature Metabolism 4, no. 12 (2022): 1756–1774. 10.1038/s42255-022-00707-5 36536134

[124] J. F. Codocedo , C. Mera‐Reina , P. B.‐C. Lin , et al., “Therapeutic Targeting of Immunometabolism Reveals a Critical Reliance on Hexokinase 2 Dosage for Microglial Activation and Alzheimer's Progression,” Cell reports 43, no. 7 (2024): 114488. 39002124 10.1016/j.celrep.2024.114488 PMC11398604

[125] S. Chung , J.‐H. Jeong , and J.‐C. Park , “Blockade of STING Activation Alleviates Microglial Dysfunction and a Broad Spectrum of Alzheimer's Disease Pathologies,” Experimental & Molecular Medicine 56, no. 9 (2024): 1936–1951. 39218977 10.1038/s12276-024-01295-y PMC11447230

[126] C.‐H. Yu , S. Davidson , C. R. Harapas , et al., “TDP‐43 Triggers Mitochondrial DNA Release via mPTP to Activate cGAS/STING in ALS,” Cell 183, no. 3 (2020): 636–649, e18. 33031745 10.1016/j.cell.2020.09.020 PMC7599077

[127] H. Y. Tan , Y. K. Yong , Y. C. Xue , et al., “cGAS and DDX41‐STING Mediated Intrinsic Immunity Spreads Intercellularly to Promote Neuroinflammation in SOD1 ALS Model,” Iscience 25, no. 6 (2022): 104404. 35712074 10.1016/j.isci.2022.104404 PMC9194172

[128] A. Jauhari , S. V. Baranov , Y. Suofu , et al., “Melatonin Inhibits Cytosolic Mitochondrial DNA–induced Neuroinflammatory Signaling in Accelerated Aging and Neurodegeneration,” The Journal of clinical investigation 130, no. 6 (2020): 3124–3136. 32182222 10.1172/JCI135026 PMC7260019

[129] B. N. Dugger and D. W. Dickson , “Pathology of Neurodegenerative Diseases,” Cold Spring Harbor perspectives in biology 9, no. 7 (2017): a028035. 28062563 10.1101/cshperspect.a028035 PMC5495060

[130] B. R. Stockwell , A. J. P. Friedmann , and H. Bayir , “Ferroptosis: A Regulated Cell Death Nexus Linking Metabolism, Redox Biology, and Disease,” Cell 171, no. 2 (2017): 273–285. 28985560 10.1016/j.cell.2017.09.021 PMC5685180

[131] S. Krasemann , C. Madore , R. Cialic , et al., “The TREM2‐APOE Pathway Drives the Transcriptional Phenotype of Dysfunctional Microglia in Neurodegenerative Diseases,” Immunity 47, no. 3 (2017): 566–581.e9. 28930663 10.1016/j.immuni.2017.08.008 PMC5719893

[132] S. Hickman , S. Izzy , P. Sen , et al., “Microglia in Neurodegeneration,” Nature Neuroscience 21, no. 10 (2018): 1359–1369. 30258234 10.1038/s41593-018-0242-x PMC6817969

[133] M. R. Elliott and K. S. Ravichandran , “The Dynamics of Apoptotic Cell Clearance,” Developmental Cell 38, no. 2 (2016): 147–160. 27459067 10.1016/j.devcel.2016.06.029 PMC4966906

[134] A. Lau and M. Tymianski , “Glutamate Receptors, Neurotoxicity and Neurodegeneration,” Pflugers Archiv: European journal of physiology 460, no. 2 (2010): 525–542. 20229265 10.1007/s00424-010-0809-1

[135] F. M. Menzies , A. Fleming , and D. C. Rubinsztein , “Compromised Autophagy and Neurodegenerative Diseases,” Nature Reviews Neuroscience 16, no. 6 (2015): 345–357. 25991442 10.1038/nrn3961

[136] M. Fricker , A. M. Tolkovsky , and V. Borutaite , Neuronal Cell Death Physiol Rev 98, no. 2 (2018): 813–880. 29488822 10.1152/physrev.00011.2017 PMC5966715

[137] J. Yuan , P. Amin , and D. Ofengeim , “Necroptosis and RIPK1‐mediated Neuroinflammation in CNS Diseases,” Nature Reviews Neuroscience 20, no. 1 (2019): 19–33. 30467385 10.1038/s41583-018-0093-1 PMC6342007

[138] S. B. De and E. Karran , “The Cellular Phase of Alzheimer's Disease,” Cell 164, no. 4 (2016): 603–615. 26871627 10.1016/j.cell.2015.12.056

[139] R. C. Paolicelli , A. Sierra , B. Stevens , et al., “Microglia States and Nomenclature: A Field at Its Crossroads,” Neuron 110, no. 21 (2022): 3458–3483. 36327895 10.1016/j.neuron.2022.10.020 PMC9999291

[140] P. Scheltens , S. B. De , M. Kivipelto , et al., “Alzheimer's Disease,” Lancet 397, no. 10284 (2021): 1577–1590. 33667416 10.1016/S0140-6736(20)32205-4 PMC8354300

[141] P. R. Angelova and A. Y. Abramov , “Role of Mitochondrial ROS in the Brain: From Physiology to Neurodegeneration,” Febs Letters 592, no. 5 (2018): 692–702. 29292494 10.1002/1873-3468.12964

[142] A. Caccamo , C. Branca , I. S. Piras , et al., “Necroptosis Activation in Alzheimer's Disease,” Nature Neuroscience 20, no. 9 (2017): 1236–1246. 28758999 10.1038/nn.4608

[143] E. F. Fang , Y. Hou , K. Palikaras , et al., “Mitophagy Inhibits Amyloid‐β and Tau Pathology and Reverses Cognitive Deficits in Models of Alzheimer's Disease,” Nature Neuroscience 22, no. 3 (2019): 401–412. 30742114 10.1038/s41593-018-0332-9 PMC6693625

[144] M. T. Heneka , M. J. Carson , K. J. El , et al., “Neuroinflammation in Alzheimer's Disease,” Lancet Neurology 14, no. 4 (2015): 388–405. 25792098 10.1016/S1474-4422(15)70016-5 PMC5909703

[145] C. Stadelmann , T. L. Deckwerth , A. Srinivasan , et al., “Activation of Caspase‐3 in Single Neurons and Autophagic Granules of Granulovacuolar Degeneration in Alzheimer's Disease: Evidence for Apoptotic Cell Death,” The American Journal of Pathology 155, no. 5 (1999): 1459–1466. 10550301 10.1016/S0002-9440(10)65460-0 PMC1866960

[146] N. Louneva , J. W. Cohen , L. Y. Han , et al., “Caspase‐3 Is Enriched in Postsynaptic Densities and Increased in Alzheimer's Disease,” American Journal of Pathology 173, no. 5 (2008): 1488–1495. 18818379 10.2353/ajpath.2008.080434 PMC2570138

[147] P. Picone , M. L. Bondi , and G. Montana , “Ferulic Acid Inhibits Oxidative Stress and Cell Death Induced by Ab Oligomers: Improved Delivery by Solid Lipid Nanoparticles,” Free Radic Res 43, no. 11 (2009): 1133–1145. 19863373 10.1080/10715760903214454

[148] M. Calvo‐Rodriguez and B. J. Bacskai , “High Mitochondrial Calcium Levels Precede Neuronal Death in Vivo in Alzheimer's Disease,” Cell Stress 4, no. 7 (2020): 187–190. 32656500 10.15698/cst2020.07.226 PMC7328672

[149] M. Calvo‐Rodriguez , S. S. Hou , A. C. Snyder , et al., “Increased Mitochondrial Calcium Levels Associated With Neuronal Death in a Mouse Model of Alzheimer's Disease,” Nat Commun 11, no. 1 (2020): 2146. 32358564 10.1038/s41467-020-16074-2 PMC7195480

[150] D. Y. Lee , K. S. Lee , H. J. Lee , et al., “Activation of PERK Signaling Attenuates Abeta‐mediated ER Stress,” PLoS ONE 5, no. 5 (2010): e10489. 20463975 10.1371/journal.pone.0010489 PMC2864758

[151] S. A. Park , G. M. Shaked , D. E. Bredesen , et al., “Mechanism of Cytotoxicity Mediated by the C31 Fragment of the Amyloid Precursor Protein,” Biochemical and Biophysical Research Communications 388, no. 2 (2009): 450–455. 19679105 10.1016/j.bbrc.2009.08.042 PMC2838490

[152] H. S. Nhan , K. Chiang , and E. H. Koo , “The Multifaceted Nature of Amyloid Precursor Protein and Its Proteolytic Fragments: Friends and Foes,” Acta Neuropathologica 129, no. 1 (2015): 1–19. 25287911 10.1007/s00401-014-1347-2 PMC4282969

[153] T. C. GAMBLIN , F. CHEN , and A. ZAMBRANO , “Caspase Cleavage of Tau: Linking Amyloid and Neurofibrillary Tangles in Alzheimer's Disease,” PNAS 100, no. 17 (2003): 10032–10037. 12888622 10.1073/pnas.1630428100 PMC187753

[154] A. NOËL , B. FOVEAU , and A. C. LEBLANC , “Caspase‐6‐Cleaved Tau Fails to Induce Tau Hyperphosphorylation and Aggregation, Neurodegeneration, Glial Inflammation, and Cognitive Deficits,” Cell Death & Disease 12, no. 3 (2021): 227. 33649324 10.1038/s41419-021-03506-0 PMC7921451

[155] C. Conze , M. Rierola , N. I. Trushina , et al., “Caspase‐cleaved Tau Is Senescence‐associated and Induces a Toxic Gain of Function by Putting a Brake on Axonal Transport,” Molecular Psychiatry 27, no. 7 (2022): 3010–3023. 35393558 10.1038/s41380-022-01538-2 PMC9205779

[156] Q. Wang , S. Guo , D. Hu , et al., “Enhanced Gasdermin‐E‐mediated Pyroptosis in Alzheimer's Disease,” Neuroscience 536 (2024): 1–11. 37944579 10.1016/j.neuroscience.2023.11.004

[157] D. Ofengeim , S. Mazzitelli , Y. Ito , et al., “RIPK1 mediates a Disease‐associated Microglial Response in Alzheimer's Disease,” PNAS 114, no. 41 (2017): E8788–e97. 10.1073/pnas.1714175114 PMC5642727 28904096

[158] W. Wang , F. Zhao , X. Ma , et al., “Mitochondria Dysfunction in the Pathogenesis of Alzheimer's Disease: Recent Advances,” Mol Neurodegener 15, no. 1 (2020): 30. 32471464 10.1186/s13024-020-00376-6 PMC7257174

[159] M. C. B. D'alessandro , S. Kanaan , M. Geller , et al., “Mitochondrial Dysfunction in Alzheimer's Disease,” Ageing Research Reviews 107 (2025): 102713. 40023293 10.1016/j.arr.2025.102713

[160] H. K. Anandatheerthavarada , G. Biswas , M. A. Robin , et al., “Mitochondrial Targeting and a Novel Transmembrane Arrest of Alzheimer's Amyloid Precursor Protein Impairs Mitochondrial Function in Neuronal Cells,” Journal of Cell Biology 161, no. 1 (2003): 41–54. 12695498 10.1083/jcb.200207030 PMC2172865

[161] L. Devi , B. M. Prabhu , and D. F. Galati , “Accumulation of Amyloid Precursor Protein in the Mitochondrial Import Channels of human Alzheimer's disease Brain Is Associated With Mitochondrial Dysfunction,” Journal of Neuroscience 26, no. 35 (2006): 9057–9068. 16943564 10.1523/JNEUROSCI.1469-06.2006 PMC6675337

[162] P. C. A. Hansson , N. Alikhani , H. Behbahani , et al., “The Amyloid Beta‐peptide Is Imported Into Mitochondria via the TOM Import Machinery and Localized to Mitochondrial Cristae,” PNAS 105, no. 35 (2008): 13145–13150. 18757748 10.1073/pnas.0806192105 PMC2527349

[163] J. W. Lustbader , M. Cirilli , and C. Lin , “ABAD Directly Links Abeta to Mitochondrial Toxicity in Alzheimer's Disease,” Science 304, no. 5669 (2004): 448–452. 15087549 10.1126/science.1091230

[164] J. Yao , H. Du , and S. Yan , “Inhibition of Amyloid‐beta (Abeta) Peptide‐binding Alcohol Dehydrogenase‐Abeta Interaction Reduces Abeta Accumulation and Improves Mitochondrial Function in a Mouse Model of Alzheimer's Disease,” Journal of Neuroscience 31, no. 6 (2011): 2313–2320. 21307267 10.1523/JNEUROSCI.4717-10.2011 PMC3381884

[165] Y. A. Lim , A. Grimm , and M. Giese , “Inhibition of the Mitochondrial Enzyme ABAD Restores the Amyloid‐β‐mediated Deregulation of Estradiol,” PLoS ONE 6, no. 12 (2011): e28887. 22174920 10.1371/journal.pone.0028887 PMC3236223

[166] M. Manczak , M. J. Calkins , and P. H. Reddy , “Impaired Mitochondrial Dynamics and Abnormal Interaction of Amyloid Beta With Mitochondrial Protein Drp1 in Neurons From Patients With Alzheimer's Disease: Implications for Neuronal Damage,” Human Molecular Genetics 20, no. 13 (2011): 2495–2509. 21459773 10.1093/hmg/ddr139 PMC3109997

[167] L. Zhang , S. Trushin , T. A. Christensen , et al., “Altered Brain Energetics Induces Mitochondrial Fission Arrest in Alzheimer's Disease,” Scientific Reports 6 (2016): 18725. 26729583 10.1038/srep18725 PMC4700525

[168] R. Kandimalla , V. Thirumala , and P. H. Reddy , “Is Alzheimer's Disease a Type 3 Diabetes? A Critical Appraisal,” Biochim Biophys Acta Mol Basis Dis 1863, no. 5 (2017): 1078–1089. 27567931 10.1016/j.bbadis.2016.08.018 PMC5344773

[169] A. Mary , F. Eysert , F. Checler , et al., “Mitophagy in Alzheimer's Disease: Molecular Defects and Therapeutic Approaches,” Molecular Psychiatry 28, no. 1 (2023): 202–216. 35665766 10.1038/s41380-022-01631-6 PMC9812780

[170] R. A. Nixon , J. Wegiel , A. Kumar , et al., “Extensive Involvement of Autophagy in Alzheimer Disease: An Immuno‐electron Microscopy Study,” Journal of Neuropathology and Experimental Neurology 64, no. 2 (2005): 113–122. 15751225 10.1093/jnen/64.2.113

[171] S. Lee , Y. Sato , and R. A. Nixon , “Primary Lysosomal Dysfunction Causes Cargo‐specific Deficits of Axonal Transport Leading to Alzheimer‐Like Neuritic Dystrophy,” Autophagy 7, no. 12 (2011): 1562–1563. 22024748 10.4161/auto.7.12.17956 PMC3327621

[172] J. H. Lee , D. S. Yang , C. N. Goulbourne , et al., “Faulty Autolysosome Acidification in Alzheimer's Disease Mouse Models Induces Autophagic Build‐up of Aβ in Neurons, Yielding Senile Plaques,” Nature Neuroscience 25 (2022): 688–701. 35654956 10.1038/s41593-022-01084-8 PMC9174056

[173] M. Oku , Y. Maeda , Y. Kagohashi , et al., “Evidence for ESCRT‐ and Clathrin‐dependent Microautophagy,” Journal of Cell Biology 216, no. 10 (2017): 3263–3274. 28838958 10.1083/jcb.201611029 PMC5626533

[174] J. Ito , S. Omiya , M. C. Rusu , et al., “Iron Derived From Autophagy‐mediated Ferritin Degradation Induces Cardiomyocyte Death and Heart Failure in Mice,” Elife 10 (2021): e62174. 33526170 10.7554/eLife.62174 PMC7853718

[175] F. wang , R. gómez‐sintes , and P. boya , “Lysosomal Membrane Permeabilization and Cell Death,” Traffic 19, no. 12 (2018): 918–931. 30125440 10.1111/tra.12613

[176] E. Piovesana , C. Magrin , M. Ciccaldo , et al., “Tau Accumulation in Degradative Organelles Is Associated to Lysosomal Stress,” Scientific Reports 13, no. 1 (2023): 18024. 37865674 10.1038/s41598-023-44979-7 PMC10590387

[177] S. Jiang and K. Bhaskar , “Degradation and Transmission of Tau by Autophagic‐Endolysosomal Networks and Potential Therapeutic Targets for Tauopathy,” Front Mol Neurosci 13 (2020): 586731. 33177989 10.3389/fnmol.2020.586731 PMC7596180

[178] B. H. Yoon , J. Kim , S. Sengupta , et al., “Targeted Autophagic Clearance of Tau Protects Against Alzheimer's Disease Through Amelioration of Tau‐mediated Lysosomal Stress,” Theranostics 15, no. 17 (2025): 9240–9260. 40963907 10.7150/thno.118409 PMC12439469

[179] A. Sobue , O. Komine , and K. Yamanaka , “Neuroinflammation in Alzheimer's Disease: Microglial Signature and Their Relevance to Disease,” Inflamm Regen 43, no. 1 (2023): 26. 37165437 10.1186/s41232-023-00277-3 PMC10170691

[180] B. L. Heckmann , B. J. W. Teubner , B. Tummers , et al., “LC3‐Associated Endocytosis Facilitates β‐Amyloid Clearance and Mitigates Neurodegeneration in Murine Alzheimer's Disease,” Cell 178, no. 3 (2019): 536–551.e14. 31257024 10.1016/j.cell.2019.05.056 PMC6689199

[181] C. Peña‐Martinez , A. D. Rickman , and B. L. Heckmann , “Beyond Autophagy: LC3‐associated Phagocytosis and Endocytosis,” Science Advances 8, no. 43 (2022): eabn1702. 36288309 10.1126/sciadv.abn1702 PMC9604515

[182] A. C. Doran , A. Yurdagul JR. , and I. Tabas , “Efferocytosis in Health and Disease,” Nature Reviews Immunology 20, no. 4 (2020): 254–267. 10.1038/s41577-019-0240-6 PMC7667664 31822793

[183] F. Guerra and C. Bucci , “Multiple Roles of the Small GTPase Rab,” Cells 5, no. 3 (2016): 34. 27548222 10.3390/cells5030034 PMC5040976

[184] K. Ghilarducci , V. C. Cabana , A. Harake , et al., “Membrane Targeting and GTPase Activity of Rab7 Are Required for Its Ubiquitination by RNF167,” International Journal of Molecular Sciences 23, no. 14 (2022): 7847. 35887194 10.3390/ijms23147847 PMC9319455

[185] M. Zhang , L. Chen , S. Wang , et al., “Rab7: Roles in Membrane Trafficking and Disease,” Bioscience Reports 29, no. 3 (2009): 193–209. 19392663 10.1042/BSR20090032

[186] J. Jung , J. Baek , K. Tae , et al., “Structural Mechanism for Regulation of Rab7 by Site‐specific Monoubiquitination,” International Journal of Biological Macromolecules 194 (2022): 347–357. 34801583 10.1016/j.ijbiomac.2021.11.074

[187] X. Guo , P. Tang , L. Chen , et al., “Amyloid β‐Induced Redistribution of Transcriptional Factor EB and Lysosomal Dysfunction in Primary Microglial Cells,” Frontiers in aging neuroscience 9 (2017): 228. 28769785 10.3389/fnagi.2017.00228 PMC5515861

[188] M. Hansen , D. C. Rubinsztein , and D. W. Walker , “Autophagy as a Promoter of Longevity: Insights From Model Organisms,” Nature Reviews Molecular Cell Biology 19, no. 9 (2018): 579–593. 30006559 10.1038/s41580-018-0033-y PMC6424591

[189] H. Mathys , J. Davila‐Velderrain , Z. Peng , et al., “Single‐cell Transcriptomic Analysis of Alzheimer's Disease,” Nature 570, no. 7761 (2019): 332–337. 31042697 10.1038/s41586-019-1195-2 PMC6865822

[190] R. Lu , L. Zhang , and X. Yang , “Interaction Between Autophagy and the NLRP3 Inflammasome in Alzheimer's and Parkinson's Disease,” Frontiers in aging neuroscience 14 (2022): 1018848. 36262883 10.3389/fnagi.2022.1018848 PMC9574200

[191] D. Zhang , Y. Zhang , J. Pan , et al., “Degradation of NLRP3 by p62‐dependent‐autophagy Improves Cognitive Function in Alzheimer's Disease by Maintaining the Phagocytic Function of Microglia,” CNS neuroscience & therapeutics 29, no. 10 (2023): 2826–2842. 37072933 10.1111/cns.14219 PMC10493665

[192] J. Marschallinger , T. Iram , M. Zardeneta , et al., “Lipid‐droplet‐accumulating Microglia Represent a Dysfunctional and Proinflammatory state in the Aging Brain,” Nature Neuroscience 23, no. 2 (2020): 194–208. 31959936 10.1038/s41593-019-0566-1 PMC7595134

[193] S. A. Liddelow , K. A. Guttenplan , L. E. Clarke , et al., “Neurotoxic Reactive Astrocytes Are Induced by Activated Microglia,” Nature 541, no. 7638 (2017): 481–487. 28099414 10.1038/nature21029 PMC5404890

[194] V. Chou , R. V. Pearse 2nd , A. J. Aylward , et al., “INPP5D regulates Inflammasome Activation in human Microglia,” Nature Communications 14, no. 1 (2023): 7552. 10.1038/s41467-023-42819-w PMC10684891 38016942

[195] P. Preman , J. Tcw , S. Calafate , et al., “Human iPSC‐derived Astrocytes Transplanted Into the Mouse Brain Undergo Morphological Changes in Response to Amyloid‐β Plaques,” Mol Neurodegener 16, no. 1 (2021): 68. 34563212 10.1186/s13024-021-00487-8 PMC8467145

[196] Z. B. Ding , L. J. Song , Q. Wang , et al., “Astrocytes: A Double‐edged Sword in Neurodegenerative Diseases,” Neural Regen Res 16, no. 9 (2021): 1702–1710. 33510058 10.4103/1673-5374.306064 PMC8328766

[197] Y. Y. Fan and J. Huo , “A1/A2 astrocytes in central Nervous System Injuries and Diseases: Angels or Devils?,” Neurochemistry International 148 (2021): 105080. 34048845 10.1016/j.neuint.2021.105080

[198] A. Herbert , “Complement Controls the Immune Synapse and Tumors Control Complement,” Journal for ImmunoTherapy of Cancer 8, no. 2 (2020): e001712. 33323465 10.1136/jitc-2020-001712 PMC7745530

[199] X. T. Cheng , Y. X. Xie , B. Zhou , et al., “Revisiting LAMP1 as a Marker for Degradative Autophagy‐lysosomal Organelles in the Nervous System,” Autophagy 14, no. 8 (2018): 1472–1474. 29940787 10.1080/15548627.2018.1482147 PMC6103665

[200] J. Park and W. S. Chung , “Astrocyte‐dependent Circuit Remodeling by Synapse Phagocytosis,” Current Opinion in Neurobiology 81 (2023): 102732. 37247606 10.1016/j.conb.2023.102732

[201] L. Li , S. Lu , J. Zhu , et al., “Astrocytes Excessively Engulf Synapses in a Mouse Model of Alzheimer's Disease,” International Journal of Molecular Sciences 25, no. 2 (2024): 1160. 38256233 10.3390/ijms25021160 PMC10816735

[202] T. Mokhtari , S. Mahahakizadeh , H. Aligholi , et al., “Triiodothyronine Improves Morphology and Upregulates Seladin‐1 of Neurospheres Extracted From Subventricular Zone in Streptozotocin‐induced Rat Model of Alzheimer's Disease,” Journal of Contemporary Medical Sciences 6, no. 1 (2020): 875.

[203] K. Zhang , D. Ma , Y. Wu , et al., “Impact of Chronic Intermittent Hypoxia on Cognitive Function and Hippocampal Neurons in Mice: A Study of Inflammatory and Oxidative Stress Pathways,” Nat Sci Sleep 16 (2024): 2029–2043. 39712883 10.2147/NSS.S489232 PMC11660659

[204] D. Lana , F. Ugolini , and M. G. Giovannini , “An Overview on the Differential Interplay among Neurons‐Astrocytes‐Microglia in CA1 and CA3 Hippocampus in Hypoxia/Ischemia,” Front Cell Neurosci 14 (2020): 585833. 33262692 10.3389/fncel.2020.585833 PMC7686560

[205] S. Mahakizadeh , T. Mokhtari , F. Navaee , et al., “Effects of Chronic Hypoxia on the Expression of Seladin‐1/Tuj1 and the Number of Dark Neurons of Hippocampus,” Journal of Chemical Neuroanatomy 104 (2020): 101744. 31926979 10.1016/j.jchemneu.2020.101744

[206] T. Mokhtari , M. Shayan , and R. A. Rezaei , “Wharton's Jelly Mesenchymal Stem Cells Attenuate Global Hypoxia‐induced Learning and Memory Impairment via Preventing Blood‐brain Barrier Breakdown,” Iran J Basic Med Sci 26, no. 9 (2023): 1053–1060. 37605722 10.22038/IJBMS.2023.70137.15250 PMC10440140

[207] J. W. Kinney , S. M. Bemiller , and A. S. Murtishaw , “Inflammation as a central Mechanism in Alzheimer's Disease,” Alzheimers Dement (N Y) 4 (2018): 575–590. 30406177 10.1016/j.trci.2018.06.014 PMC6214864

[208] M. Blevins H , Y. Xu , S. Biby , et al., “The NLRP3 Inflammasome Pathway: A Review of Mechanisms and Inhibitors for the Treatment of Inflammatory Diseases,” Frontiers in aging neuroscience 14 (2022): 879021. 35754962 10.3389/fnagi.2022.879021 PMC9226403

[209] M. F. Gulen , N. Samson , A. Keller , et al., “cGAS‐STING Drives Ageing‐related Inflammation and Neurodegeneration,” Nature 620, no. 7973 (2023): 374–380. 37532932 10.1038/s41586-023-06373-1 PMC10412454

[210] K. E. Campagno and C. H. Mitchell , “The P2X(7) Receptor in Microglial Cells Modulates the Endolysosomal Axis, Autophagy, and Phagocytosis,” Front Cell Neurosci 15 (2021): 645244. 33790743 10.3389/fncel.2021.645244 PMC8005553

[211] L. H. Fairley , J. H. Wong , and A. M. Barron , “Mitochondrial Regulation of Microglial Immunometabolism in Alzheimer's Disease,” Frontiers in immunology 12 (2021): 624538. 33717134 10.3389/fimmu.2021.624538 PMC7947196

[212] P. Calabresi , A. Mechelli , and G. Natale , “Alpha‐synuclein in Parkinson's Disease and Other Synucleinopathies: From Overt Neurodegeneration Back to Early Synaptic Dysfunction,” Cell death & disease 14, no. 3 (2023): 176. 36859484 10.1038/s41419-023-05672-9 PMC9977911

[213] I. Silva , M. F. D. Silva , and T. S. A. Dutra , “Alpha‐synuclein Aggregation in Parkinson's Disease,” Adv Protein Chem Struct Biol 146 (2025): 35–75. 40610077 10.1016/bs.apcsb.2024.11.002

[214] A. Jahabardeen , N. S , N. J , et al., “A Review on the Role of SNCA Gene in Neurodegenerative Diseases,” Cureus 16, no. 9 (2024): e69450. 39411638 10.7759/cureus.69450 PMC11476641

[215] J. Ko C , S. L. Gao , T. K. Lin , et al., “Ferroptosis as a Major Factor and Therapeutic Target for Neuroinflammation in Parkinson's Disease,” Biomedicines 9, no. 11 (2021): 1679. 34829907 10.3390/biomedicines9111679 PMC8615560

[216] Q. Ru , Y. Li , L. Chen , et al., “Iron Homeostasis and Ferroptosis in human Diseases: Mechanisms and Therapeutic Prospects,” Signal Transduct Target Ther 9, no. 1 (2024): 271. 39396974 10.1038/s41392-024-01969-z PMC11486532

[217] X. Guo , R. Wei , X. Yin , et al., “Crosstalk Between Neuroinflammation and Ferroptosis: Implications for Parkinson's Disease Progression,” Frontiers in pharmacology 16 (2025): 1528538. 40183096 10.3389/fphar.2025.1528538 PMC11966490

[218] L. T. N. Nguyen , H. D. Nguyen , Y. J. Kim , et al., “Role of NLRP3 Inflammasome in Parkinson's Disease and Therapeutic Considerations,” J Parkinsons Dis 12, no. 7 (2022): 2117–2133. 35988226 10.3233/JPD-223290 PMC9661339

[219] M. T. Henrich , W. H. Oertel , D. J. Surmeier , et al., “Mitochondrial Dysfunction in Parkinson's Disease—a Key Disease Hallmark With Therapeutic Potential,” Mol Neurodegener 18, no. 1 (2023): 83. 37951933 10.1186/s13024-023-00676-7 PMC10640762

[220] Z. H. Lin , Y. Liu , N. J. Xue , et al., “Quercetin Protects Against MPP(+)/MPTP‐Induced Dopaminergic Neuron Death in Parkinson's Disease by Inhibiting Ferroptosis,” Oxid Med Cell Longev 2022 (2022): 7769355. 36105483 10.1155/2022/7769355 PMC9467739

[221] A. S. Wilkins , R. W. Wrangham , and W. T. Fitch , “The “Domestication Syndrome” in Mammals: A Unified Explanation Based on Neural Crest Cell Behavior and Genetics,” Genetics 197, no. 3 (2014): 795–808. 25024034 10.1534/genetics.114.165423 PMC4096361

[222] A. Iannielli , S. Bido , L. Folladori , et al., “Pharmacological Inhibition of Necroptosis Protects From Dopaminergic Neuronal Cell Death in Parkinson's Disease Models,” Cell reports 22, no. 8 (2018): 2066–2079. 29466734 10.1016/j.celrep.2018.01.089 PMC5842028

[223] Q. S. Lin , P. Chen , W. X. Wang , et al., “RIP1/RIP3/MLKL Mediates Dopaminergic Neuron Necroptosis in a Mouse Model of Parkinson disease,” Laboratory Investigation 100, no. 3 (2020): 503–511. 31506635 10.1038/s41374-019-0319-5

[224] H. Li , J. Zhang , Y. Shen , et al., “Targeting Mitochondrial Complex I Deficiency in MPP(+)/MPTP‐induced Parkinson's Disease Cell Culture and Mouse Models by Transducing Yeast NDI1 Gene,” Biol Proced Online 26, no. 1 (2024): 9. 38594619 10.1186/s12575-024-00236-3 PMC11003148

[225] Y. Chen , C. Chen , D. Song , et al., “Dexmedetomidine Protects SH‐SY5Y Cells Against MPP(+) ‐induced Declining of Mitochondrial Membrane Potential and Cell Cycle Deficits,” European Journal of Neuroscience 54, no. 1 (2021): 4141–4153. 10.1111/ejn.15252 33905578

[226] V. Viswanath , Y. Wu , R. Boonplueang , et al., “Caspase‐9 Activation Results in Downstream Caspase‐8 Activation and Bid Cleavage in 1‐methyl‐4‐phenyl‐1,2,3,6‐tetrahydropyridine‐induced Parkinson's Disease,” Journal of Neuroscience 21, no. 24 (2001): 9519–9528. 11739563 10.1523/JNEUROSCI.21-24-09519.2001 PMC6763046

[227] S. GHOSH , J. WON S , J. WANG , et al., “α‐synuclein Aggregates Induce c‐Abl Activation and Dopaminergic Neuronal Loss by a Feed‐forward Redox Stress Mechanism,” Progress in Neurobiology 202 (2021): 102070. 33951536 10.1016/j.pneurobio.2021.102070 PMC8210833

[228] F. F. Geibl , M. T. Henrich , Z. Xie , et al., “α‐Synuclein Pathology Disrupts Mitochondrial Function in Dopaminergic and Cholinergic Neurons at‐risk in Parkinson's disease,” Mol Neurodegener 19, no. 1 (2024): 69. 39379975 10.1186/s13024-024-00756-2 PMC11462807

[229] M. R. Di , P. J. Barrett , and E. K. Hoffman , “α‐Synuclein Binds to TOM20 and Inhibits Mitochondrial Protein Import in Parkinson's disease,” Science Translational Medicine 8, no. 342 (2016): 342ra78. 10.1126/scitranslmed.aaf3634 PMC5016095 27280685

[230] L. F. Burbulla , P. Song , and J. R. Mazzulli , “Dopamine Oxidation Mediates Mitochondrial and Lysosomal Dysfunction in Parkinson's disease,” Science 357, no. 6357 (2017): 1255–1261. 28882997 10.1126/science.aam9080 PMC6021018

[231] V. Tapias , X. Hu , and K. C. Luk , “Synthetic Alpha‐synuclein Fibrils Cause Mitochondrial Impairment and Selective Dopamine Neurodegeneration in Part via iNOS‐mediated Nitric Oxide Production,” Cellular and Molecular Life Sciences 74, no. 15 (2017): 2851–2874. 28534083 10.1007/s00018-017-2541-x PMC5524146

[232] A. M. Pickrell and R. J. Youle , “The Roles of PINK1, Parkin, and Mitochondrial Fidelity in Parkinson's Disease,” Neuron 85, no. 2 (2015): 257–273. 25611507 10.1016/j.neuron.2014.12.007 PMC4764997

[233] C. Blauwendraat , M. A. Nalls , and A. B. Singleton , “The Genetic Architecture of Parkinson's Disease,” Lancet Neurology 19, no. 2 (2020): 170–178. 31521533 10.1016/S1474-4422(19)30287-X PMC8972299

[234] H. Ariga , K. Takahashi‐Niki , I. Kato , et al., “Neuroprotective Function of DJ‐1 in Parkinson's Disease,” Oxid Med Cell Longev 2013 (2013): 683920. 23766857 10.1155/2013/683920 PMC3671546

[235] L. D. Skou , S. K. Johansen , and J. Okarmus , “Pathogenesis of DJ‐1/PARK7‐Mediated Parkinson's Disease,” Cells 13, no. 4 (2024): 296. 38391909 10.3390/cells13040296 PMC10887164

[236] M. López‐Aguirre , T. Balzano , and M. H. G. Monje , “Nigrostriatal Iron Accumulation in the Progression of Parkinson's Disease,” NPJ Parkinsons Dis 11, no. 1 (2025): 72. 40216790 10.1038/s41531-025-00911-6 PMC11992180

[237] E. Alushaj , A. Kuurstra , and R. S. Menon , “Midbrain and Pallidal Iron Changes Identify Patients With REM Sleep Behaviour Disorder and Parkinson's Disease,” NPJ Parkinsons Dis 11, no. 1 (2025): 84. 40268921 10.1038/s41531-025-00916-1 PMC12019255

[238] J. X. Ren , X. Sun , and X. L. Yan , “Ferroptosis in Neurological Diseases,” Front Cell Neurosci 14 (2020): 218. 32754017 10.3389/fncel.2020.00218 PMC7370841

[239] S. Zhu , Q. Zhang , X. Sun , et al., “HSPA5 Regulates Ferroptotic Cell Death in Cancer Cells,” Cancer Research 77, no. 8 (2017): 2064–2077. 28130223 10.1158/0008-5472.CAN-16-1979 PMC5392369

[240] J. Wu , A. M. Minikes , and M. Gao , “Intercellular Interaction Dictates Cancer Cell Ferroptosis via NF2‐YAP Signalling,” Nature 572, no. 7769 (2019): 402–406. 31341276 10.1038/s41586-019-1426-6 PMC6697195

[241] Y. Sun , Y. Zheng , C. Wang , et al., “Glutathione Depletion Induces Ferroptosis, Autophagy, and Premature Cell Senescence in Retinal Pigment Epithelial Cells,” Cell death & disease 9, no. 7 (2018): 753. 29988039 10.1038/s41419-018-0794-4 PMC6037763

[242] Z. L. Wang , L. Yuan , and W. Li , “Ferroptosis in Parkinson's Disease: Glia‐neuron Crosstalk,” Trends in Molecular Medicine 28, no. 4 (2022): 258–269. 35260343 10.1016/j.molmed.2022.02.003

[243] K. J. Lin , S. D. Chen , and K. L. Lin , “Iron Brain Menace: The Involvement of Ferroptosis in Parkinson Disease,” Cells 11, no. 23 (2022): 3829. 36497089 10.3390/cells11233829 PMC9735800

[244] A. Martin‐Bastida , R. J. Ward , R. Newbould , et al., “Brain Iron Chelation by deferiprone in a Phase 2 Randomised Double‐blinded Placebo Controlled Clinical Trial in Parkinson's Disease,” Scientific Reports 7, no. 1 (2017): 1398. 28469157 10.1038/s41598-017-01402-2 PMC5431100

[245] D. Devos , J. Labreuche , O. Rascol , et al., “Trial of Deferiprone in Parkinson's Disease,” New England Journal of Medicine 387, no. 22 (2022): 2045–2055. 36449420 10.1056/NEJMoa2209254

[246] J. P. Taylor , R. H. Brown Jr. , and D. W. cleveland , “Decoding ALS: From Genes to Mechanism,” Nature 539, no. 7628 (2016): 197–206. 27830784 10.1038/nature20413 PMC5585017

[247] A. M. Blokhuis , E. J. Groen , M. Koppers , et al., “Protein Aggregation in Amyotrophic Lateral Sclerosis,” Acta Neuropathologica 125, no. 6 (2013): 777–794. 23673820 10.1007/s00401-013-1125-6 PMC3661910

[248] C. Hetz and S. Saxena , “ER Stress and the Unfolded Protein Response in Neurodegeneration,” Nature reviews Neurology 13, no. 8 (2017): 477–491. 28731040 10.1038/nrneurol.2017.99

[249] J. D. Malhotra and R. J. Kaufman , “The Endoplasmic Reticulum and the Unfolded Protein Response,” Seminars in cell & developmental biology 18, no. 6 (2007): 716–731. 18023214 10.1016/j.semcdb.2007.09.003 PMC2706143

[250] J. D. Rothstein , K. M. Van , and A. I. Levey , “Selective Loss of Glial Glutamate Transporter GLT‐1 in Amyotrophic Lateral sclerosis,” Annals of Neurology 38, no. 1 (1995): 73–84. 7611729 10.1002/ana.410380114

[251] Y. A. Ojaimi , A. Dangoumau , H. Alarcan , et al., “TAR DNA‐binding Protein of 43 kDa (TDP‐43) and Amyotrophic Lateral Sclerosis (ALS): A Promising Therapeutic Target,” Expert Opinion on Therapeutic Targets 26, no. 6 (2022): 575–592. 35652285 10.1080/14728222.2022.2083958

[252] A. K. Walker , K. Y. Soo , V. Sundaramoorthy , et al., “ALS‐associated TDP‐43 Induces Endoplasmic Reticulum Stress, Which Drives Cytoplasmic TDP‐43 Accumulation and Stress Granule Formation,” PLoS ONE 8, no. 11 (2013): e81170. 24312274 10.1371/journal.pone.0081170 PMC3843686

[253] S. Lépine , A. Nauleau‐Javaudin , E. Deneault , et al., “Homozygous ALS‐linked Mutations in TARDBP/TDP‐43 Lead to Hypoactivity and Synaptic Abnormalities in human iPSC‐derived Motor Neurons,” Iscience 27, no. 3 (2024): 109166. 38433895 10.1016/j.isci.2024.109166 PMC10905001

[254] T. Murakami , S. Qamar , J. Q. Lin , et al., “ALS/FTD Mutation‐Induced Phase Transition of FUS Liquid Droplets and Reversible Hydrogels Into Irreversible Hydrogels Impairs RNP Granule Function,” Neuron 88, no. 4 (2015): 678–690. 26526393 10.1016/j.neuron.2015.10.030 PMC4660210

[255] A. O. Aladesuyi , J. E. Stauffer , S. Saberi , et al., “Antisense RNA Foci Are Associated With Nucleoli and TDP‐43 Mislocalization in C9orf72‐ALS/FTD: A Quantitative Study,” Acta Neuropathologica 137, no. 3 (2019): 527–530. 30666413 10.1007/s00401-018-01955-0 PMC6397670

[256] H. Zhu , “Interference of Nuclear Speckles: A Nexus of RNA Foci, Dipeptide Repeats, and Mis‐splicing in C9ORF72 ALS/FTD,” Neuron 112, no. 20 (2024): 3375–3377. 39447539 10.1016/j.neuron.2024.10.001

[257] S. Ryan , S. Rollinson , and E. Hobbs , “C9orf72 dipeptides Disrupt the Nucleocytoplasmic Transport Machinery and Cause TDP‐43 Mislocalisation to the Cytoplasm,” Scientific Reports 12, no. 1 (2022): 4799. 35314728 10.1038/s41598-022-08724-w PMC8938440

[258] S. Saberi , J. E. Stauffer , J. Jiang , et al., “Sense‐encoded Poly‐GR Dipeptide Repeat Proteins Correlate to Neurodegeneration and Uniquely co‐localize With TDP‐43 in Dendrites of Repeat‐expanded C9orf72 Amyotrophic Lateral Sclerosis,” Acta Neuropathologica 135, no. 3 (2018): 459–474. 29196813 10.1007/s00401-017-1793-8 PMC5935138

[259] S. Leskelä , N. Huber , D. Hoffmann , et al., “Expression of C9orf72 Hexanucleotide Repeat Expansion Leads to Formation of RNA Foci and Dipeptide Repeat Proteins but Does Not Influence Autophagy or Proteasomal Function in Neuronal Cells,” Biochimica et Biophysica Acta ‐ Molecular Cell Research 1868, no. 7 (2021): 119021. 33775797 10.1016/j.bbamcr.2021.119021

[260] X. Wen , W. Tan , T. Westergard , et al., “Antisense Proline‐arginine RAN Dipeptides Linked to C9ORF72‐ALS/FTD Form Toxic Nuclear Aggregates That Initiate in Vitro and in Vivo Neuronal Death,” Neuron 84, no. 6 (2014): 1213–1225. 25521377 10.1016/j.neuron.2014.12.010 PMC4632245

[261] W. Scheper and J. J. Hoozemans , “The Unfolded Protein Response in Neurodegenerative Diseases: A Neuropathological Perspective,” Acta Neuropathologica 130, no. 3 (2015): 315–331. 26210990 10.1007/s00401-015-1462-8 PMC4541706

[262] D. Hughes and G. R. Mallucci , “The Unfolded Protein Response in Neurodegenerative Disorders—therapeutic Modulation of the PERK Pathway,” Febs Journal 286, no. 2 (2019): 342–355. 29476642 10.1111/febs.14422

[263] C. Hetz and F. R. Papa , “The Unfolded Protein Response and Cell Fate Control,” Molecular Cell 69, no. 2 (2018): 169–181. 29107536 10.1016/j.molcel.2017.06.017

[264] W. Luan , A. L. Wright , H. Brown‐Wright , et al., “Early Activation of Cellular Stress and Death Pathways Caused by Cytoplasmic TDP‐43 in the rNLS8 Mouse Model of ALS and FTD,” Molecular Psychiatry 28, no. 6 (2023): 2445–2461. 37012334 10.1038/s41380-023-02036-9 PMC10611572

[265] K. Watabe , Y. Kato , M. Sakuma , et al., “Praja1 RING‐finger E3 Ubiquitin Ligase Suppresses Neuronal Cytoplasmic TDP‐43 Aggregate Formation,” Neuropathology 40, no. 6 (2020): 570–586. 32686212 10.1111/neup.12694 PMC7818255

[266] J. H. Qiu , A. Asai , S. Chi , et al., “Proteasome Inhibitors Induce Cytochrome c‐caspase‐3‐Like Protease‐mediated Apoptosis in Cultured Cortical Neurons,” Journal of Neuroscience 20, no. 1 (2000): 259–265. 10627603 10.1523/JNEUROSCI.20-01-00259.2000 PMC6774120

[267] J. Van Eersel , Y. D. Ke , A. Gladbach , et al., “Cytoplasmic Accumulation and Aggregation of TDP‐43 Upon Proteasome Inhibition in Cultured Neurons,” PLoS ONE 6, no. 7 (2011): e22850. 21829535 10.1371/journal.pone.0022850 PMC3146516

[268] B. Khosravi , H. Hartmann , S. May , et al., “Cytoplasmic Poly‐GA Aggregates Impair Nuclear Import of TDP‐43 in C9orf72 ALS/FTLD,” Human Molecular Genetics 26, no. 4 (2017): 790–800. 28040728 10.1093/hmg/ddw432 PMC5409121

[269] E. Foran and D. Trotti , “Glutamate Transporters and the Excitotoxic Path to Motor Neuron Degeneration in Amyotrophic Lateral sclerosis,” Antioxid Redox Signaling 11, no. 7 (2009): 1587–1602. 10.1089/ars.2009.2444 PMC2842587 19413484

[270] F. J. Arnold , A. F. Putka , U. Raychaudhuri , et al., “Revisiting Glutamate Excitotoxicity in Amyotrophic Lateral Sclerosis and Age‐Related Neurodegeneration,” International Journal of Molecular Sciences 25, no. 11 (2024): 5587. 38891774 10.3390/ijms25115587 PMC11171854

[271] W. Zhou and R. Xu , “Current Insights in the Molecular Genetic Pathogenesis of Amyotrophic Lateral Sclerosis,” Frontiers in neuroscience 17 (2023): 1189470. 37638324 10.3389/fnins.2023.1189470 PMC10448825

[272] Y. Zhang , W. Liu , T. Huang , et al., “Neuroprotective Role of GLT‐1/EAAT2 in Glutamate‐induced Excitotoxicity,” Behavioural Brain Research 495 (2025): 115804. 40914332 10.1016/j.bbr.2025.115804

[273] M. Tischbein , D. M. Baron , Y. C. Lin , et al., “The RNA‐binding Protein FUS/TLS Undergoes Calcium‐mediated Nuclear Egress During Excitotoxic Stress and Is Required for GRIA2 mRNA Processing,” Journal of Biological Chemistry 294, no. 26 (2019): 10194–10210. 31092554 10.1074/jbc.RA118.005933 PMC6664169

[274] E. Abgueguen , M. Tortarolo , L. Rouviere , et al., “Sephin1 reduces TDP‐43 Cytoplasmic Mislocalization and Improves Motor Neuron Survival in ALS Models,” Life Sci Alliance 8, no. 9 (2025): e202403195. 40602832 10.26508/lsa.202403195 PMC12225689

[275] C. A. Ross and S. J. Tabrizi , “Huntington's Disease: From Molecular Pathogenesis to Clinical Treatment,” Lancet Neurology 10, no. 1 (2011): 83–98. 21163446 10.1016/S1474-4422(10)70245-3

[276] C. Zuccato , M. Valenza , and E. Cattaneo , “Molecular Mechanisms and Potential Therapeutical Targets in Huntington's Disease,” Physiological Reviews 90, no. 3 (2010): 905–981. 20664076 10.1152/physrev.00041.2009

[277] V. Costa and L. Scorrano , “Shaping the Role of Mitochondria in the Pathogenesis of Huntington's Disease,” Embo Journal 31, no. 8 (2012): 1853–1864. 22446390 10.1038/emboj.2012.65 PMC3343341

[278] P. Guedes‐Dias , B. R. Pinho , T. R. Soares , et al., “Mitochondrial Dynamics and Quality Control in Huntington's disease,” Neurobiology of Disease 90 (2016): 51–57. 26388396 10.1016/j.nbd.2015.09.008

[279] J. Y. Shin , Z. H. Fang , Z. X. Yu , et al., “Expression of Mutant Huntingtin in Glial Cells Contributes to Neuronal Excitotoxicity,” Journal of Cell Biology 171, no. 6 (2005): 1001–1012. 16365166 10.1083/jcb.200508072 PMC2171327

[280] B. Diaz‐Castro , M. R. Gangwani , X. Yu , et al., “Astrocyte Molecular Signatures in Huntington's disease,” Science Translational Medicine 11, no. 514 (2019): eaaw8546. 31619545 10.1126/scitranslmed.aaw8546

[281] T. H. Palpagama , M. Sagniez , S. Kim , et al., “GABA(A) Receptors Are Well Preserved in the Hippocampus of Aged Mice,” eNeuro 6, no. 4 (2019): ENEURO.0496‐18.2019. 10.1523/ENEURO.0496-18.2019 PMC6709233 31340951

[282] K. Pircs , J. Drouin‐Ouellet , V. Horváth , et al., “Distinct Subcellular Autophagy Impairments in Induced Neurons From Patients With Huntington's Disease,” Brain 145, no. 9 (2022): 3035–3057. 34936701 10.1093/brain/awab473 PMC9473361

[283] M. Martinez‐Vicente , Z. Talloczy , E. Wong , et al., “Cargo Recognition Failure Is Responsible for Inefficient Autophagy in Huntington's disease,” Nature Neuroscience 13, no. 5 (2010): 567–576. 20383138 10.1038/nn.2528 PMC2860687

[284] J. Ochaba , T. Lukacsovich , G. Csikos , et al., “Potential Function for the Huntingtin Protein as a Scaffold for Selective Autophagy,” PNAS 111, no. 47 (2014): 16889–16894. 25385587 10.1073/pnas.1420103111 PMC4250109

[285] F. Saudou and S. Humbert , “The Biology of Huntingtin,” Neuron 89, no. 5 (2016): 910–926. 26938440 10.1016/j.neuron.2016.02.003

[286] P. Rusmini , F. Mina , and M. Valenza , “Impairment of Lysosomal Quality Control in Huntington disease,” Cell death & disease 16, no. 1 (2025): 762. 41145409 10.1038/s41419-025-08103-z PMC12559425

[287] J. Ojalvo‐Pacheco , S. M. S. Yakhine‐Diop , J. M. Fuentes , et al., “Role of TFEB in Huntington's Disease,” Biology (Basel) 13, no. 4 (2024): 238. 38666850 10.3390/biology13040238 PMC11048341

[288] Y. C. Wong and E. L. Holzbaur , “Optineurin Is an Autophagy Receptor for Damaged Mitochondria in Parkin‐mediated Mitophagy That Is Disrupted by an ALS‐linked Mutation,” PNAS 111, no. 42 (2014): E4439–48. 10.1073/pnas.1405752111 PMC4210283 25294927

[289] J. Bradford , J. Y. Shin , M. Roberts , et al., “Expression of Mutant Huntingtin in Mouse Brain Astrocytes Causes Age‐dependent Neurological Symptoms,” PNAS 106, no. 52 (2009): 22480–22485. 20018729 10.1073/pnas.0911503106 PMC2799722

[290] M. Faideau , J. Kim , K. Cormier , et al., “In Vivo Expression of Polyglutamine‐expanded Huntingtin by Mouse Striatal Astrocytes Impairs Glutamate Transport: A Correlation With Huntington's Disease Subjects,” Human Molecular Genetics 19, no. 15 (2010): 3053–3067. 20494921 10.1093/hmg/ddq212 PMC2901144

[291] E. Paldino , V. D'angelo , G. Sancesario , et al., “Pyroptotic Cell Death in the R6/2 Mouse Model of Huntington's disease: New Insight on the Inflammasome,” Cell Death Discov 6 (2020): 69. 32821438 10.1038/s41420-020-00293-z PMC7395807

[292] E. Paldino , V. D'angelo , D. Laurenti , et al., “Modulation of Inflammasome and Pyroptosis by Olaparib, a PARP‐1 Inhibitor, in the R6/2 Mouse Model of Huntington's Disease,” Cells 9, no. 10 (2020): 2286. 33066292 10.3390/cells9102286 PMC7602058

[293] X. Guo , M. H. Disatnik , M. Monbureau , et al., “Inhibition of Mitochondrial Fragmentation Diminishes Huntington's Disease‐associated Neurodegeneration,” Journal of Clinical Investigation 123, no. 12 (2013): 5371–5388. 24231356 10.1172/JCI70911 PMC3859413

[294] U. Shirendeb , A. P. Reddy , M. Manczak , et al., “Abnormal Mitochondrial Dynamics, Mitochondrial Loss and Mutant Huntingtin Oligomers in Huntington's disease: Implications for Selective Neuronal Damage,” Human Molecular Genetics 20, no. 7 (2011): 1438–1455. 21257639 10.1093/hmg/ddr024 PMC3049363

[295] T. Milakovic and G. V. Johnson , “Mitochondrial Respiration and ATP Production Are Significantly Impaired in Striatal Cells Expressing Mutant Huntingtin,” Journal of Biological Chemistry 280, no. 35 (2005): 30773–30782. 15983033 10.1074/jbc.M504749200

[296] S. Ayala‐Peña , “Role of Oxidative DNA Damage in Mitochondrial Dysfunction and Huntington's Disease Pathogenesis,” Free Radic Biol Med 62 (2013): 102–110. 23602907 10.1016/j.freeradbiomed.2013.04.017 PMC3722255

[297] Q. Tang , H. Liu , and X. J. Shi , “Blood Oxidative Stress Marker Aberrations in Patients With Huntington's Disease: A Meta‐Analysis Study,” Oxid Med Cell Longev 2020 (2020): 9187195. 32963705 10.1155/2020/9187195 PMC7499314

[298] L. López‐Molina , A. Pereda‐Velarde , and F. N. Di , “Mitochondria From huntington´s Disease Striatal Astrocytes Are Hypermetabolic and Compromise Neuronal Branching,” Cell Communication and Signaling 23, no. 1 (2025): 341. 40671084 10.1186/s12964-025-02341-6 PMC12265250

[299] A. A. Polyzos , D. Y. Lee , R. Datta , et al., “Metabolic Reprogramming in Astrocytes Distinguishes Region‐Specific Neuronal Susceptibility in Huntington Mice,” Cell metabolism 29, no. 6 (2019): 1258–1273.e11. 30930170 10.1016/j.cmet.2019.03.004 PMC6583797

[300] S. Song , Z. Su , N. Kon , et al., “ALOX5‐mediated Ferroptosis Acts as a Distinct Cell Death Pathway Upon Oxidative Stress in Huntington's Disease,” Genes & development 37, no. 5‐6 (2023): 204–217. 36921996 10.1101/gad.350211.122 PMC10111862

[301] X. Liu , F. Wang , and X. Fan , “CHCHD2 up‐regulation in Huntington Disease Mediates a Compensatory Protective Response Against Oxidative Stress,” Cell death & disease 15, no. 2 (2024): 126. 38341417 10.1038/s41419-024-06523-x PMC10858906

[302] S. Upadhayay and P. Kumar , “Mitochondrial Targeted Antioxidants as Potential Therapy for huntington's Disease,” Pharmacol Rep 76, no. 4 (2024): 693–713. 38982016 10.1007/s43440-024-00619-z

[303] A. Hincelin‐Mery , X. Nicolas , C. Cantalloube , et al., “Safety, Pharmacokinetics, and Target Engagement of a Brain Penetrant RIPK1 Inhibitor, SAR443820 (DNL788), in Healthy Adult Participants,” Clin Transl Sci 17, no. 1 (2024): e13690. 38010108 10.1111/cts.13690 PMC10772668

[304] L. Wu , M. Liu , and J. Liang , “Ferroptosis as a New Mechanism in Parkinson's Disease Therapy Using Traditional Chinese Medicine,” Frontiers in pharmacology 12 (2021): 659584. 34163356 10.3389/fphar.2021.659584 PMC8215498

[305] K. Yang , Y. Liu , and W. Deng , “Astrocytes Contribute to Motor Neuron Degeneration in ALS via the TRAIL‐DR5 Signaling Pathway,” Journal of Neurochemistry 169, no. 7 (2025): e70146. 40641248 10.1111/jnc.70146 PMC12246772

[306] B. Mallika , K. Sudha , A. Massand , et al., “Evaluation of Neuroprotective Role of Benfotiamine in Alzheimer's disease Model: A Randomized Control Study,” Clinica Terapeutica 176, no. 2 (2025): 127–135. 40176579 10.7417/CT.2025.5195

[307] R. Li , X. Han , Q. Wang , et al., “Network Pharmacology Analysis and Clinical Efficacy of the Traditional Chinese Medicine Bu‐Shen‐Jian‐Pi. Part 3: Alleviation of Hypoxia, Muscle‐wasting, and Modulation of Redox Functions in Amyotrophic Lateral Sclerosis,” International Journal of Clinical Pharmacology and Therapeutics 62, no. 4 (2024): 169–177. 38431830 10.5414/CP204520

[308] J. García‐Revilla , R. Ruiz , A. M. Espinosa‐Oliva , et al., “Dopaminergic Neurons Lacking Caspase‐3 Avoid Apoptosis but Undergo Necrosis After MPTP Treatment Inducing a Galectin‐3‐dependent Selective Microglial Phagocytic Response,” Cell death & disease 15, no. 8 (2024): 625. 39223107 10.1038/s41419-024-07014-9 PMC11369297

[309] D. Zheng , Y. Lai , K. Huang , et al., “Pyroptosis Mediated by Parkin‐NLRP3 Negative Feedback Loop Contributed to Parkinson's Disease Induced by Rotenone,” International Immunopharmacology 143, no. Pt 3 (2024): 113608. 39549548 10.1016/j.intimp.2024.113608

[310] E. Gatlik , B. Mehes , E. Voltz , et al., “First‐in‐human Safety, Tolerability, and Pharmacokinetic Results of DFV890, an Oral Low‐molecular‐weight NLRP3 Inhibitor,” Clin Transl Sci 17, no. 5 (2024): e13789. 38761014 10.1111/cts.13789 PMC11101992

[311] Y. Yong , L. Yan , and J. Wei , “A Novel Ferroptosis Inhibitor, Thonningianin A, Improves Alzheimer's Disease by Activating GPX4,” Theranostics 14, no. 16 (2024): 6161–6184. 39431016 10.7150/thno.98172 PMC11488096

[312] L. Tan , J. Xie , C. Liao , et al., “Tetrahedral Framework Nucleic Acids Inhibit Aβ‐mediated Ferroptosis and Ameliorate Cognitive and Synaptic Impairments in Alzheimer's Disease,” J Nanobiotechnology 22, no. 1 (2024): 682. 39506721 10.1186/s12951-024-02963-x PMC11542373

[313] X. Zha , X. Liu , and M. Wei , “Microbiota‐derived Lysophosphatidylcholine Alleviates Alzheimer's Disease Pathology via Suppressing Ferroptosis,” Cell metabolism 37, no. 1 (2025): 169–186.e9. 39510074 10.1016/j.cmet.2024.10.006

[314] N. Majerníková , M. J. Caiado , and R. I. Seinstra , “Ferroptosis Inhibition Protects Against α‐synuclein‐related Neuronal Cell Death,” Cell death & disease 17, no. 1 (2025): 78. 41390672 10.1038/s41419-025-08319-z PMC12827279

[315] X. DONG , T. YANG , and Z. JIN , “Formononetin Derived From Parabacteroides merdae Alleviates MPTP‐induced Parkinson's Disease in Mice by Inhibiting Ferroptosis via the PI3K‐AKT‐ferritinophagy Axis,” Communications Biology 8, no. 1 (2025): 1672. 41291228 10.1038/s42003-025-09067-8 PMC12647768

[316] Q. Ma , J. Wu , H. Li , et al., “The Role of TRPV4 in Ferroptosis in MPP(+)/MPTP‐induced Parkinson's Disease Models,” Tissue & Cell 96 (2025): 103019. 40554139 10.1016/j.tice.2025.103019

[317] N. Okada , S. Nakamura , and M. Shimazawa , “3‐Nitropropionic Acid Enhances Ferroptotic Cell Death via NOX2‐Mediated ROS Generation in STHdhQ111 Striatal Cells Carrying Mutant Huntingtin,” Biological & pharmaceutical bulletin 46, no. 2 (2023): 177–186. 36724946 10.1248/bpb.b22-00529

[318] J. Yan , L. Bao , and H. Liang , “A Druglike Ferrostatin‐1 Analogue as a Ferroptosis Inhibitor and Photoluminescent Indicator,” Angewandte Chemie 64, no. 20 (2025): e202502195. 39984310 10.1002/anie.202502195

[319] J. Koper M , S. Moonen , A. Ronisz , et al., “Inhibition of an Alzheimer's Disease‐associated Form of Necroptosis Rescues Neuronal Death in Mouse Models,” Science Translational Medicine 16, no. 771 (2024): eadf5128. 39475569 10.1126/scitranslmed.adf5128

[320] Z. Song , X. Xie , and Y. Chen , “Innate Immune Sensing of Z‐nucleic Acids by ZBP1‐RIPK1 Axis Drives Neuroinflammation in Alzheimer's Disease,” Immunity 58, no. 10 (2025): 2574–2592.e9. 40902587 10.1016/j.immuni.2025.07.024

[321] M. Zelic , A. Blazier , F. Pontarelli , et al., “Single‐cell Transcriptomic and Functional Studies Identify Glial state Changes and a Role for Inflammatory RIPK1 Signaling in ALS Pathogenesis,” Immunity 58, no. 4 (2025): 961–979.e8. 40132594 10.1016/j.immuni.2025.02.024

[322] L. Geng , W. Gao , H. Saiyin , et al., “MLKL Deficiency Alleviates Neuroinflammation and Motor Deficits in the α‐synuclein Transgenic Mouse Model of Parkinson's disease,” Mol Neurodegener 18, no. 1 (2023): 94. 38041169 10.1186/s13024-023-00686-5 PMC10693130

[323] M. Vissers , J. Heuberger , and G. J. Groeneveld , “Safety, Pharmacokinetics and Target Engagement of Novel RIPK1 Inhibitor SAR443060 (DNL747) for Neurodegenerative Disorders: Randomized, Placebo‐controlled, Double‐blind Phase I/Ib Studies in Healthy Subjects and Patients,” Clin Transl Sci 15, no. 8 (2022): 2010–2023. 35649245 10.1111/cts.13317 PMC9372423

[324] O. M. Gonen , T. Porter , and B. Wang , “Safety, Pharmacokinetics and Target Engagement of a Novel Brain Penetrant RIPK1 Inhibitor (SIR9900) in Healthy Adults and Elderly Participants,” Clin Transl Sci 18, no. 2 (2025): e70151. 39960838 10.1111/cts.70151 PMC11832029

[325] N. S. Jones , S. Kshirsagar , and V. Mohanan , “A Phase I, Randomized, Ascending‐dose Study to Assess Safety, Pharmacokinetics, and Activity of GDC‐8264, a RIP1 Inhibitor, in Healthy Volunteers,” Clin Transl Sci 16, no. 10 (2023): 1997–2009. 37596814 10.1111/cts.13607 PMC10582670

[326] J. Lickliter , S. Wang , and W. Zhang , “A Phase I Randomized, Double‐blinded, Placebo‐controlled Study Assessing the Safety and Pharmacokinetics of RIPK1 Inhibitor GFH312 in Healthy Subjects,” Clin Transl Sci 16, no. 9 (2023): 1691–1703. 37345561 10.1111/cts.13580 PMC10499419

[327] M. Han , D. H. Seo , and M. S. Kwak , “Apomorphine Is a Novel Necroptosis Inhibitor Targeting Mixed Lineage Kinase Domain‐Like Protein Oligomerization,” Cell Death Discov 11, no. 1 (2025): 457. 41083456 10.1038/s41420-025-02763-8 PMC12518639

[328] Y. Hou , X. Chu , and J. H. Park , “Urolithin A Improves Alzheimer's Disease Cognition and Restores Mitophagy and Lysosomal Functions,” Alzheimers Dement 20, no. 6 (2024): 4212–4233. 38753870 10.1002/alz.13847 PMC11180933

[329] W. Q. Qiu , L. Yu , and C. L. He , “Two 18‐norspirostane Steroidal Saponins as Novel Mitophagy Enhancers Improve Alzheimer's Disease,” Clinical and translational medicine 13, no. 9 (2023): e1390. 37658612 10.1002/ctm2.1390 PMC10474309

[330] S. Macfarlane , T. Grimmer , and K. Teo , “Blarcamesine for the Treatment of Early Alzheimer's Disease: Results From the ANAVEX2‐73‐AD‐004 Phase IIB/III Trial,” J Prev Alzheimers Dis 12, no. 1 (2025): 100016. 39800452 10.1016/j.tjpad.2024.100016 PMC12184016

[331] Y. Zhuo , S. Li W , W. Lu , et al., “TGF‐β1 Mediates Hypoxia‐preconditioned Olfactory Mucosa Mesenchymal Stem Cells Improved Neural Functional Recovery in Parkinson's disease Models and Patients,” Mil Med Res 11, no. 1 (2024): 48. 39034405 10.1186/s40779-024-00550-7 PMC11265117

[332] S. Salomon‐Zimri , A. Pushett , N. Russek‐Blum , et al., “Combination of Ciprofloxacin/celecoxib as a Novel Therapeutic Strategy for ALS,” Amyotroph Lateral Scler Frontotemporal Degener 24, no. 3‐4 (2023): 263–271. 36106817 10.1080/21678421.2022.2119868

[333] J. Mandrioli , R. D'amico , E. Zucchi , et al., “Randomized, Double‐blind, Placebo‐controlled Trial of Rapamycin in Amyotrophic Lateral Sclerosis,” Nature Communications 14, no. 1 (2023): 4970. 10.1038/s41467-023-40734-8 PMC10435464 37591957

[334] I. Lenoel , M. Ribon , F. Lorenc , et al., “ALS/FTD‐linked TBK1 Deficiency in Microglia Induces an Aged‐Like Microglial Signature and Drives Social Recognition Deficits in Mice,” Nature Communications 16, no. 1 (2025): 7951. 10.1038/s41467-025-63211-w PMC12381211 40858618

[335] J. Beckers , A. K. Tharkeshwar , and D. P. Van , “C9orf72 ALS‐FTD: Recent Evidence for Dysregulation of the Autophagy‐lysosome Pathway at Multiple Levels,” Autophagy 17, no. 11 (2021): 3306–3322. 33632058 10.1080/15548627.2021.1872189 PMC8632097

[336] Safety and Efficacy of Trehalose in Amyotrophic Lateral Sclerosis (HEALEY ALS Platform Trial): An Adaptive, Phase 2/3, Double‐blind, Randomised, Placebo‐controlled Trial. *Lancet Neurology* 2025, 24(6): 500–511. 10.1016/S1474-4422(25)00173-5 40409314

[337] Y. Li , M. Sun , F. Cao , et al., “The Ferroptosis Inhibitor Liproxstatin‐1 Ameliorates LPS‐Induced Cognitive Impairment in Mice,” Nutrients 14, no. 21 (2022): 4599. 36364859 10.3390/nu14214599 PMC9656387

[338] L. Chen , R. Na , M. K. Danae , et al., “Overexpression of Ferroptosis Defense Enzyme Gpx4 Retards Motor Neuron Disease of SOD1G93A Mice,” Scientific Reports 11, no. 1 (2021): 12890. 34145375 10.1038/s41598-021-92369-8 PMC8213805

[339] C. Guo , Y. X. Zhang , T. Wang , et al., “Intranasal Deferoxamine Attenuates Synapse Loss via Up‐regulating the P38/HIF‐1α Pathway on the Brain of APP/PS1 Transgenic Mice,” Frontiers in aging neuroscience 7 (2015): 104. 26082716 10.3389/fnagi.2015.00104 PMC4451419

[340] S. Huang , Z. Chen , B. Fan , et al., “A Selective NLRP3 Inflammasome Inhibitor Attenuates Behavioral Deficits and Neuroinflammation in a Mouse Model of Parkinson's Disease,” Journal of Neuroimmunology 354 (2021): 577543. 33714750 10.1016/j.jneuroim.2021.577543

[341] V. Deora , J. D. Lee , E. A. Albornoz , et al., “The Microglial NLRP3 Inflammasome Is Activated by Amyotrophic Lateral Sclerosis Proteins,” Glia 68, no. 2 (2020): 407–421. 31596526 10.1002/glia.23728

[342] C. W. Olanow , A. H. Schapira , P. A. Lewitt , et al., “TCH346 as a Neuroprotective Drug in Parkinson's disease: A Double‐blind, Randomised, Controlled Trial,” Lancet Neurology 5, no. 12 (2006): 1013–1020. 17110281 10.1016/S1474-4422(06)70602-0

[343] P. H. Gordon , D. H. Moore , R. G. Miller , et al., “Efficacy of Minocycline in Patients With Amyotrophic Lateral Sclerosis: A Phase III Randomised Trial,” Lancet Neurology 6, no. 12 (2007): 1045–1053. 17980667 10.1016/S1474-4422(07)70270-3

[344] S. P. Aggarwal , L. Zinman , E. Simpson , et al., “Safety and Efficacy of Lithium in Combination With riluzole for Treatment of Amyotrophic Lateral Sclerosis: A Randomised, Double‐blind, Placebo‐controlled Trial,” Lancet Neurology 9, no. 5 (2010): 481–488. 20363190 10.1016/S1474-4422(10)70068-5 PMC3071495

[345] Y. Li and L. Q. Zhou , “dCas9 techniques for Transcriptional Repression in Mammalian Cells: Progress, Applications and Challenges,” BioEssays 43, no. 9 (2021): e2100086. 34327721 10.1002/bies.202100086

[346] Y. A. Chen , M. W. Kankel , S. Hana , et al., “In Vivo Genome Editing Using Novel AAV‐PHP Variants Rescues Motor Function Deficits and Extends Survival in a SOD1‐ALS Mouse Model,” Gene Therapy 30, no. 5 (2023): 443–454. 36450833 10.1038/s41434-022-00375-w PMC9713118

[347] H. X. Deng , H. Zhai , Y. Shi , et al., “Efficacy and Long‐term Safety of CRISPR/Cas9 Genome Editing in the SOD1‐linked Mouse Models of ALS,” Communications Biology 4, no. 1 (2021): 396. 33767386 10.1038/s42003-021-01942-4 PMC7994668

[348] R. Martier and P. Konstantinova , “Gene Therapy for Neurodegenerative Diseases: Slowing down the Ticking Clock,” Frontiers in neuroscience 14 (2020): 580179. 33071748 10.3389/fnins.2020.580179 PMC7530328

[349] T. M. Dawson , T. E. Golde , and C. Lagier‐Tourenne , “Animal Models of Neurodegenerative Diseases,” Nature neuroscience 21, no. 10 (2018): 1370–1379. 30250265 10.1038/s41593-018-0236-8 PMC6615039

[350] A. L. Young , R. V. Marinescu , N. P. Oxtoby , et al., “Uncovering the Heterogeneity and Temporal Complexity of Neurodegenerative Diseases With Subtype and Stage Inference,” Nature communications 9, no. 1 (2018): 4273. 10.1038/s41467-018-05892-0 PMC6189176 30323170

[351] Y. Cao and J. Yang , “Mechanisms of Pathological Axonal Degeneration,” T. S. Tran , A. Yaron , eds. Wiring the Nervous System: Mechanisms of Axonal and Dendritic Remodelling in Health and Disease Abingdon: River Publishers (2024): 215–250. 40036397

[352] A. M. Grabrucker , B. Ruozi , D. Belletti , et al., “Nanoparticle Transport Across the Blood Brain Barrier,” Tissue Barriers 4, no. 1 (2016): e1153568. 27141426 10.1080/21688370.2016.1153568 PMC4836460

[353] C. Johnson , D. L. Burkhart , and K. M. Haigis , “Classification of KRAS‐Activating Mutations and the Implications for Therapeutic Intervention,” Cancer discovery 12, no. 4 (2022): 913–923. 35373279 10.1158/2159-8290.CD-22-0035 PMC8988514

[354] A. Ayub and S. Wettig , “An Overview of Nanotechnologies for Drug Delivery to the Brain,” Pharmaceutics 14, no. 2 (2022): 224. 35213957 10.3390/pharmaceutics14020224 PMC8875260

[355] L. Alvarez‐Erviti , Y. Seow , H. Yin , et al., “Delivery of siRNA to the Mouse Brain by Systemic Injection of Targeted Exosomes,” Nature Biotechnology 29, no. 4 (2011): 341–345. 10.1038/nbt.1807 21423189

[356] M. R. P. Elmore , L. A. Hohsfield , E. A. Kramár , et al., “Replacement of Microglia in the Aged Brain Reverses Cognitive, Synaptic, and Neuronal Deficits in Mice,” Aging Cell 17, no. 6 (2018): e12832. 30276955 10.1111/acel.12832 PMC6260908

[357] X. Li , X. Gao , W. Zhang , et al., “Microglial Replacement in the Aged Brain Restricts Neuroinflammation Following Intracerebral Hemorrhage,” Cell death & disease 13, no. 1 (2022): 33. 35013119 10.1038/s41419-021-04424-x PMC8748975

[358] J. P. Chadarevian , S. I. Lombroso , G. C. Peet , et al., “Engineering an Inhibitor‐resistant human CSF1R Variant for Microglia Replacement,” Journal of Experimental Medicine 220, no. 3 (2023): e20220857. 36584406 10.1084/jem.20220857 PMC9814156

[359] D. S. Svoboda , M. I. Barrasa , J. Shu , et al., “Human iPSC‐derived Microglia Assume a Primary Microglia‐Like state After Transplantation Into the Neonatal Mouse Brain,” PNAS 116, no. 50 (2019): 25293–25303. 31772018 10.1073/pnas.1913541116 PMC6911218

[360] A. Ogaki , Y. Ikegaya , and R. Koyama , “Replacement of Mouse Microglia with Human Induced Pluripotent Stem Cell (hiPSC)‐Derived Microglia in Mouse Organotypic Slice Cultures,” Front Cell Neurosci 16 (2022): 918442. 35910250 10.3389/fncel.2022.918442 PMC9325970

[361] J. L. Cummings , C. E. Teunissen , and B. K. Fiske , “Biomarker‐guided Decision Making in Clinical Drug Development for Neurodegenerative Disorders,” Nature Reviews Drug Discovery 24, no. 8 (2025): 589–609. 40185982 10.1038/s41573-025-01165-w

[362] N. Vijiaratnam and T. Foltynie , “How Should We be Using Biomarkers in Trials of Disease Modification in Parkinson's Disease?,” Brain 146, no. 12 (2023): 4845–4869. 37536279 10.1093/brain/awad265 PMC10690028

[363] M. F. Vissers , J. A. Heuberger , and G. J. Groeneveld , “Targeting for Success: Demonstrating Proof‐of‐concept With Mechanistic Early Phase Clinical Pharmacology Studies for Disease‐modification in Neurodegenerative Disorders,” International Journal of Molecular Sciences 22, no. 4 (2021): 1615. 33562713 10.3390/ijms22041615 PMC7915613

[364] S. E. Arnold , B. T. Hyman , and R. A. Betensky , “Pathways to Personalized Medicine—Embracing Heterogeneity for Progress in Clinical Therapeutics Research in Alzheimer's Disease,” Alzheimer's & Dementia 20, no. 10 (2024): 7384–7394. 10.1002/alz.14063 PMC11485305 39240044

[365] Y. Fei and Y. Ding , “The Role of Ferroptosis in Neurodegenerative Diseases,” Frontiers in cellular neuroscience 18 (2024): 1475934. 39473490 10.3389/fncel.2024.1475934 PMC11518764

[366] J. S. Tauskela , M. Bourourou , and N. Blondeau , “Tackling Issues in the Path Toward Clinical Translation in Brain Conditioning: Potential Offered by Nutraceuticals,” Brain circulation 3, no. 2 (2017): 78–86. 30276308 10.4103/bc.bc_8_17 PMC6126266

[367] Y. Liao , X. Wang , L. Huang , et al., “Mechanism of Pyroptosis in Neurodegenerative Diseases and Its Therapeutic Potential by Traditional Chinese Medicine,” Frontiers in Pharmacology 14 (2023): 1122104. 36713841 10.3389/fphar.2023.1122104 PMC9880437

[368] D. J. Klionsky , A. K. Abdel‐Aziz , S. Abdelfatah , et al., “Guidelines for the Use and Interpretation of Assays for Monitoring Autophagy,” autophagy 17, no. 1 (2021): 1–382. 33634751 10.1080/15548627.2020.1797280 PMC7996087

[369] D. M. Wilson , M. R. Cookson , D. B. L. Van , et al., “Hallmarks of Neurodegenerative Diseases,” Cell 186, no. 4 (2023): 693–714. 36803602 10.1016/j.cell.2022.12.032

[370] H. F. YAN , T. ZOU , and Q. Z. TUO , “Ferroptosis: Mechanisms and Links With Diseases,” Signal Transduct Target Ther 6, no. 1 (2021): 49. 33536413 10.1038/s41392-020-00428-9 PMC7858612

[371] S. Masaldan , A. I. Bush , D. Devos , et al., “Striking While the Iron Is Hot: Iron Metabolism and Ferroptosis in Neurodegeneration,” Free Radic Biol Med 133 (2019): 221–233. 30266679 10.1016/j.freeradbiomed.2018.09.033

[372] F. M. Menzies , A. Fleming , and A. Caricasole , “Autophagy and Neurodegeneration: Pathogenic Mechanisms and Therapeutic Opportunities,” Neuron 93, no. 5 (2017): 1015–1034. 28279350 10.1016/j.neuron.2017.01.022

[373] S. Herculano‐Houzel , “The human Brain in Numbers: A Linearly Scaled‐up Primate Brain,” Front Hum Neurosci 3 (2009): 31. 19915731 10.3389/neuro.09.031.2009 PMC2776484

[374] T. Masuda , R. Sankowski , and O. Staszewski , “Spatial and Temporal Heterogeneity of Mouse and human Microglia at Single‐cell Resolution,” Nature 566, no. 7744 (2019): 388–392. 30760929 10.1038/s41586-019-0924-x

[375] E. Drummond and T. Wisniewski , “Alzheimer's disease: Experimental Models and Reality,” Acta Neuropathologica 133, no. 2 (2017): 155–175. 28025715 10.1007/s00401-016-1662-x PMC5253109

[376] M. Khalil , C. E. Teunissen , M. Otto , et al., “Neurofilaments as Biomarkers in Neurological Disorders,” Nature reviews Neurology 14, no. 10 (2018): 577–589. 30171200 10.1038/s41582-018-0058-z

[377] Y. Kim K , K. Y. Shin , and K. A. Chang , “GFAP as a Potential Biomarker for Alzheimer's Disease: A Systematic Review and Meta‐Analysis,” Cells 12, no. 9 (2023): 1309. 37174709 10.3390/cells12091309 PMC10177296

[378] O. Y. Glushakova , A. A. Glushakov , and D. S. Wijesinghe , “Prospective Clinical Biomarkers of Caspase‐mediated Apoptosis Associated With Neuronal and Neurovascular Damage Following Stroke and Other Severe Brain Injuries: Implications for Chronic Neurodegeneration,” Brain Circ 3, no. 2 (2017): 87–108. 30276309 10.4103/bc.bc_27_16 PMC6126261

[379] O. V. Taso , A. Philippou , A. Moustogiannis , et al., “Lipid Peroxidation Products and Their Role in Neurodegenerative Diseases,” Annals of research hospitals 3 (2019): 2.

[380] J. Cui , S. Zhao , and Y. Li , “Regulated Cell Death: Discovery, Features and Implications for Neurodegenerative Diseases,” Cell Communication and Signaling 19, no. 1 (2021): 120. 34922574 10.1186/s12964-021-00799-8 PMC8684172

[381] E. Nutma , N. Fancy , and M. Weinert , “Translocator Protein Is a Marker of Activated Microglia in Rodent Models but Not human Neurodegenerative Diseases,” Nature Communications 14, no. 1 (2023): 5247. 10.1038/s41467-023-40937-z PMC10462763 37640701

[382] P. M. Cogswell and A. P. Fan , “Multimodal Comparisons of QSM and PET in Neurodegeneration and Aging,” Neuroimage 273 (2023): 120068. 37003447 10.1016/j.neuroimage.2023.120068 PMC10947478

[383] J. L. Cummings , T. Morstorf , and K. Zhong , “Alzheimer's disease Drug‐development Pipeline: Few Candidates, Frequent Failures,” Alzheimer's research & therapy 6, no. 4 (2014): 37. 10.1186/alzrt269 PMC4095696 25024750

[384] C. K. Kim , Y. R. Lee , and L. Ong , “Alzheimer's Disease: Key Insights From Two Decades of Clinical Trial Failures,” Journal of Alzheimer's Disease 87, no. 1 (2022): 83–100. 10.3233/JAD-215699 PMC9198803 35342092

[385] R. M. Ransohoff , “All (animal) Models (of neurodegeneration) Are Wrong. Are They Also Useful?,” Journal of Experimental Medicine 215, no. 12 (2018): 2955–2958. 30459159 10.1084/jem.20182042 PMC6279414

[386] W. M. Pardridge , “Blood‐brain Barrier Delivery,” Drug Discov Today 12, no. 1‐2 (2007): 54–61. 17198973 10.1016/j.drudis.2006.10.013

[387] R. L. Prentice , “Surrogate Endpoints in Clinical Trials: Definition and Operational Criteria,” Statistics in Medicine 8, no. 4 (1989): 431–440. 2727467 10.1002/sim.4780080407

[388] M. D. R. Crapper , A. J. Dalton , T. P. Kruck , et al., “Intramuscular Desferrioxamine in Patients With Alzheimer's Disease,” Lancet 337, no. 8753 (1991): 1304–1308. 1674295 10.1016/0140-6736(91)92978-b

[389] S. Ayton , D. Barton , and B. Brew , “Deferiprone in Alzheimer Disease: A Randomized Clinical Trial,” JAMA neurology 82, no. 1 (2025): 11–18. 39495531 10.1001/jamaneurol.2024.3733 PMC11536302

[390] E. Miyoshi , S. Morabito , and C. M. Henningfield , “Spatial and Single‐nucleus Transcriptomic Analysis of Genetic and Sporadic Forms of Alzheimer's Disease,” Nature Genetics 56, no. 12 (2024): 2704–2717. 39578645 10.1038/s41588-024-01961-x PMC11631771

[391] W. Li , C. Chen , and X. Zhu , “Single‐Cell and Spatial Multiomics: Applications for Diseases,” MedComm 6, no. 12 (2025): e70553. 41403916 10.1002/mco2.70553 PMC12703058

[392] C. Gao , J. Jiang , Y. Tan , et al., “Microglia in Neurodegenerative Diseases: Mechanism and Potential Therapeutic Targets,” Signal transduction and targeted therapy 8, no. 1 (2023): 359. 37735487 10.1038/s41392-023-01588-0 PMC10514343

[393] N. Urrestizala‐Arenaza , S. Cerchio , and F. Cavaliere , “Limitations of human Brain Organoids to Study Neurodegenerative Diseases: A Manual to Survive,” Frontiers in Cellular Neuroscience 18 (2024): 1419526. 39049825 10.3389/fncel.2024.1419526 PMC11267621

[394] S. Amartumur , H. Nguyen , T. Huynh , et al., “Neuropathogenesis‐on‐chips for Neurodegenerative Diseases,” Nature communications 15, no. 1 (2024): 2219. 10.1038/s41467-024-46554-8 PMC10933492 38472255

[395] A. Helm , P. Shaffer , G. Gonzalez , et al., “Protocol of a Randomized Controlled Trial Examining Psychosocial Enhancement and Standard Medication Treatment for co‐occurring Opioid Use and Mental Health Disorders: A Half Fractional Factorial Randomized Controlled Trial,” Contemporary clinical trials 145 (2024): 107668. 39163904 10.1016/j.cct.2024.107668 PMC11392628

[396] T. M. Dawson , T. E. Golde , and C. Lagier‐Tourenne , “Animal Models of Neurodegenerative Diseases,” Nature Neuroscience 21, no. 10 (2018): 1370–1379. 30250265 10.1038/s41593-018-0236-8 PMC6615039

